# Single‐Cell RNA Sequencing Delineates Renal Anti‐Fibrotic Mechanisms Mediated by TRPC6 Inhibition

**DOI:** 10.1002/advs.202501175

**Published:** 2025-06-17

**Authors:** Yao Xu, Zhihuang Zheng, Marleen Silke Oswald, Guozhe Cheng, Jun Liu, Qidi Zhai, Ute Kruegel, Michael Schaefer, Holger Gerhardt, Nicole Endlich, Maik Gollasch, Stefan Simm, Dmitry Tsvetkov

**Affiliations:** ^1^ Department of Internal Medicine and Geriatrics University Medicine Greifswald 17487 Greifswald Germany; ^2^ Department of Nephrology Shanghai General Hospital Shanghai Jiaotong University School of Medicine Shanghai 200080 China; ^3^ Laboratory of Nephropathy Translational Medicine Center Shanghai General Hospital Shanghai Jiao Tong University School of Medicine Shanghai 201620 China; ^4^ Rudolf Boehm Institute for Pharmacology and Toxicology Leipzig University 04107 Leipzig Germany; ^5^ Max‐Delbrück‐Centrum für Molekulare Medizin 13092 Berlin Germany; ^6^ Department of Anatomy and Cell Biology University Medicine Greifswald 17487 Greifswald Germany; ^7^ Institute of Bioinformatics University Medicine Greifswald 17487 Greifswald Germany; ^8^ Faculty of Applied Natural Sciences and Health Coburg University of Applied Sciences and Art 09561 Coburg Germany

**Keywords:** chronic kidney disease, renal fibrosis, single‐cell rna sequencing, spatial transcriptomics

## Abstract

Chronic kidney disease (CKD) is characterized by persistent inflammation and tubulointerstitial fibrosis leading to end‐stage renal disease. Transient receptor potential canonical 6 (TRPC6) channel inhibition mitigates tubular injury and renal fibrosis in murine models of unilateral ureteral obstruction (UUO) and 2‐month chronic post–ischemia‐reperfusion injury (2m post‐I/R). Through integrated analysis of single‐cell‐RNA‐sequencing (scRNA‐Seq) data from UUO mice treated with the selective TRPC6 inhibitor SH045, here the renoprotective cell composition and cell type‐specific transcriptional programs are defined. We explored translational aspects by conducting an in‐depth scRNA‐Seq analysis of kidney samples from patients with CKD. These results reveal global transcriptional shifts with a dramatic diversification of inflammatory cells, endothelial cells and fibroblasts. Notably, a distinct subpopulation of novel endothelial cells is delineated, which is termed ECRIN, that regulate inflammatory networks implicating VEGF and GAS signaling pathways. The data also indicates that inhibition of TRPC6 channels triggers a *Prnp* transcription factor regulatory network, which contributes to the alleviation of renal fibrosis. The key findings are supported at the protein level by immunofluorescence and western blot analysis. We observed similar patterns in the chronic 2m postI/R injury model. These findings provide novel insights into the potential therapeutic benefits of TRPC6 inhibition in CKD.

## Graphical abstract

Single‐cell transcriptomics reveals how TRPC6 inhibition alters renal cell composition and gene expression in CKD. The study uncovers a novel endothelial subpopulation (ECRIN), highlights key inflammatory and fibrotic pathways, and identifies a Prnp‐driven network linked to fibrosis resolution, offering mechanistic insight into TRPC6 as a potential therapeutic target in CKD.

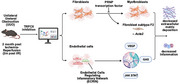

## 1 Introduction

Chronic kidney disease (CKD) represents a gradual decline in kidney function, characterized by elevated morbidity and mortality rates. CKD affects a substantial proportion of the adult and aged human population, particularly those with diabetes and hypertension.^[^ ^1^ ^]^ The main pathological features of CKD include persistent low‐grade renal inflammation and tubulointerstitial fibrosis. The complex interplay of myofibroblasts, lymphocytes, tubular and other cell types in the kidney leads to excessive deposition of extracellular matrix (ECM) and further deterioration of kidney function. The main type and origin of ECM producing cells is controversial.^[^ ^2^ ^]^ Myofibroblasts and non‐mesenchymal cells such as macrophages, monocytes, have been identified to contribute to fibrosis.^[^ ^3^ ^]^ Possible sources of myofibroblasts include interstitial fibroblasts of the kidney, vascular pericytes, mesenchymal stem cells (MSCs) derived from bone marrow, or epithelial and endothelial cells undergoing mesenchymal transition (EMT or EndMT).^[^ ^4^, ^5^, ^6^, ^7^ ^]^ Despite the availability of general treatment options, including blood pressure‐lowering medications, renin‐angiotensin system inhibitors, and sodium‐glucose transporter 2 (SGLT2) inhibitors, there is still a lack of targeted pharmacological interventions that can effectively halt the progression of CKD.

Transient receptor potential cation channels, subfamily C, member 6 (TRPC6), have recently garnered significant attention due to evidence indicating their involvement in fibrogenesis.^[^ ^8^, ^9^, ^10^ ^]^ Notably, TRPC6 blockade protects against fibrosis in murine hearts and kidneys.^[^ ^11^, ^12^ ^]^ Two distinct TRPC6 antagonists have been shown to ameliorate renal fibrosis caused by unilateral ureteral obstruction (UUO).^[^ ^13^, ^14^, ^15^ ^]^ SH045 (larixyl N‐methylcarbamate) is a potent TRPC6 blocker (EC_50_, 6 nmol/L) with an excellent selectivity profile toward TRPC6 versus TRPC3/TRPC7 channels (12‐ and 5‐fold, respectively). Its synthesis in bulk quantities is straightforward and cost‐effective, owing to the high natural abundance of the precursor (+)‐larixol.^[^ ^15^ ^]^ Moreover, pharmacokinetic studies demonstrated that the compound reaches the kidney at therapeutically relevant concentrations following in vivo administration, without evidence of toxicity ^[^ ^16^, ^17^ ^]^ However, the exact renoprotective mechanisms are unknown. In this study, we utilized single‐cell RNA sequencing (scRNA‐Seq) to define the cellular and transcriptional landscape associated with renoprotection through in vivo TRPC6 inhibition, specifically using SH045, in the UUO model. To substantiate our findings, we employed the 2‐month chronic post–ischemia‐reperfusion (2m post I/R) model, which closely mirrors the fibrotic pathology observed in chronic kidney disease (CKD).^[^ ^18^ ^]^ Our hypothesis is that SH045 specifically acts on the renal endothelium to reduce inflammation and fibrosis in the kidney. We explored human translational aspects through in‐depth scRNA‐Seq analysis of kidney samples from patients with CKD.

## 2 Results

### 2.1 Renoprotection of SH045 is Associated with Changes in Cell Composition in the Kidneys

**Figure 1:**
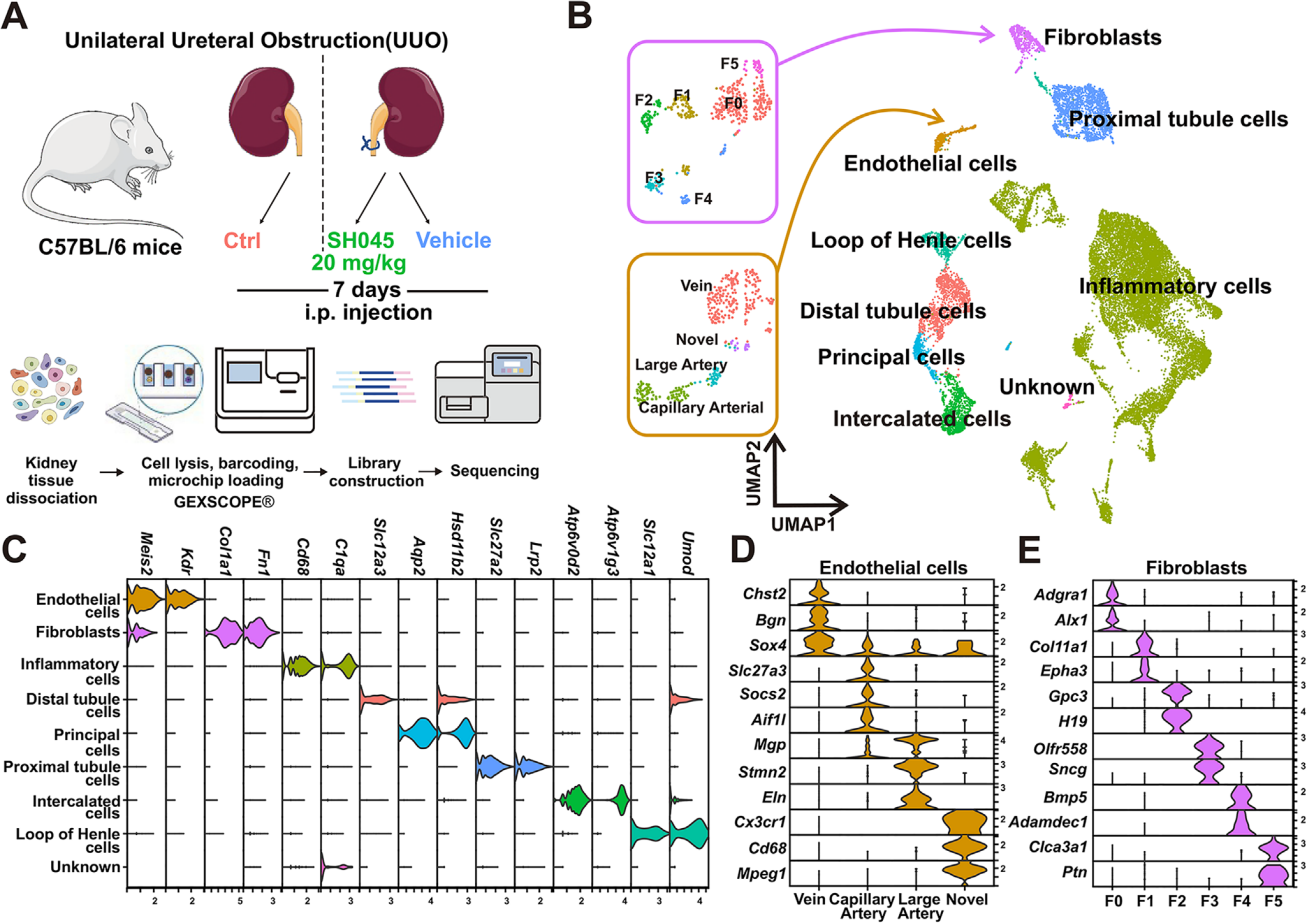
Impact of SH045 on cell diversity in mouse unilateral ureteral obstruction (UUO) model characterized by single‐cell transcriptomic analysis (scRNA‐Seq). A) Experimental design of UUO and scRNA‐Seq workflow. Mice were subjected to UUO and then injected with SH045 (n = 3) or vehicle (n = 3) once every 24 h between day 0 and day 7. Ctrl group includes kidneys that were not subjected to the UUO (n = 3). B) Uniform manifold approximation and projection (UMAP) plot showing different cell types and clusters of endothelial cells and fibroblasts from UUO and Vehicle kidneys. C) Violin plots showing the expression levels of representative marker genes across major cell types. The x axis shows the log‐scale normalized read count. Violin plots showing representative marker genes across endothelial cells D) and fibroblast E). The y axis shows the log‐scale normalized read count.

**Figure 2:**
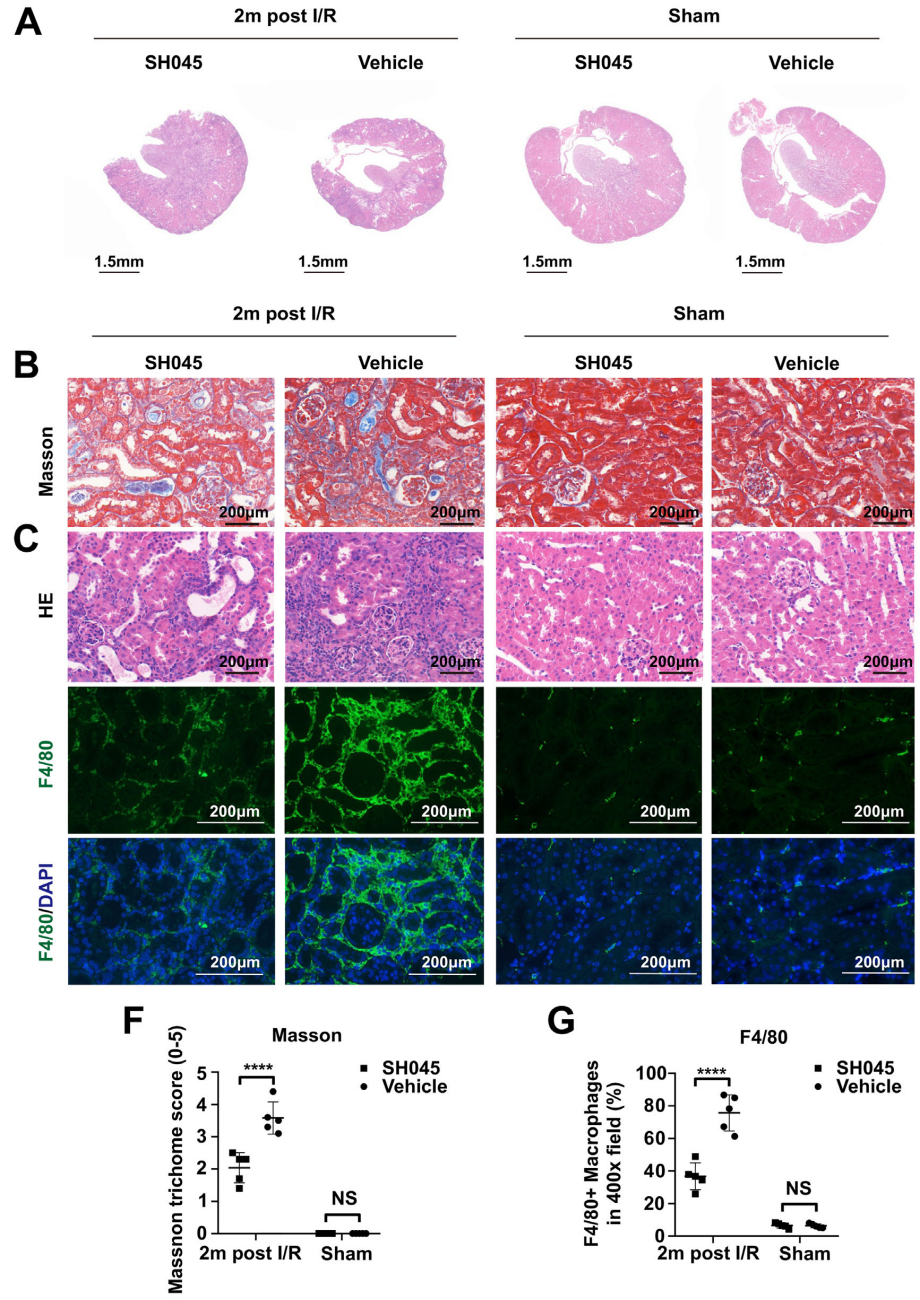
SH045 impact on kidney in 2‐month post ischemia‐reperfusion (2m post I/R) model. A) Representative images of kidney sections 2m post I/R (left panel) and Sham (right panel) groups (scale bar: 1.5 mm). Representative images of I/R and Sham kidneys stained with Masson’ trichrome B), hematoxylin‐eosin (HE) C) and F4/80+ cells D,E) (magnification: 400x, scale bar: 200 µm). Quantification of Masson's trichome score F) and renal F4/80+cell infiltration G). (2m post I/R n = 5, Sham n = 5). Data expressed as mean ± SEM. Two‐way ANOVA followed by Sidak's multiple comparisons post hoc test was used. **** *p* < 0.0001, NS, not statistically significant.

To define changes in the cell composition involved in renoprotective effects of TRPC6 inhibition in the UUO model, we conducted scRNA‐Seq using microfluidic chip technology. During the one‐week period, we administrated SH045 or Vehicle once daily (**Figure** **1**A). Consistent with our previous finding,^[^ ^14^ ^]^ SH045 reduced fibrosis, and decreased inflammatory cell infiltration in both UUO and 2m post I/R models (**Figure** **2**; Figure S1, Supporting Information). Expression of colocalized TRPC6 and α‐SMA was elevated in both UUO kidneys and kidneys of patients with CKD (Figure S2A,B, Supporting Information). Interestingly, SH045 treatment decreased the colocalization between TRPC6 and α‐SMA in UUO and 2m post I/R models (Figures S2C,D and S3, Supporting Information). We isolated and sequenced a total of 24626 cells from a kidney part derived from UUO (Vehicle and SH045) kidneys. Following quality control measures (Figure S4, Supporting Information), we acquired harmonization of 19788 cells from kidney samples with SH045 treatment (8954 cells, three mice) and Vehicle (10834 cells, three mice) for further downstream analysis.

By utilizing joint unbiased clustering, established marker genes^[^ ^19^, ^20^ ^]^ and automatic cell assignment using the ScType platform,^[^ ^21^ ^]^ we identified nine major cell types (Figure 1B). The unbiased clustering of cell populations in combination with the known cell type–specific marker genes like *Kdr* (encoding vascular endothelial growth factor receptor 2) for endothelial cells, *Col1a1* (collagen I) and *Fn1* (fibronectin 1) for fibroblasts, *Slc12a1* (Na‐K‐2Cl cotransporter) for the loop of Henle cells, and *Slc12a3* (thiazide‐sensitive sodium chloride cotransporter) for the distal convoluted tubule cells (Figure 1C–E) (Figure S5, Supporting Information) led to a clear cell‐type assignment. In addition, we describe four and six subtypes of endothelial cells and fibroblasts, respectively. SH045 treatment augmented the number of endothelial cells (relative change on log2 scale: 0.48) and fibroblasts (0.76) and reduced the number of inflammatory cells (relative change ‐0.37) (Figure S6, Supporting Information). The number of other cell types did not change upon the SH045 treatment. The inflammatory cell cluster included seven immune cell types identified by manual annotation (Figure S7A, Supporting Information). SH045 led to less renal immune cell infiltration predominantly due to Dendritic cells (log‐fold change ‐1.06) and T cells (log‐fold change 0.73) (Figure S7B, Supporting Information).

### 2.2 SH045 Leads to Transcriptomic Changes in Endothelial Cells and Fibroblasts

To gain further insights into the transcriptomic changes of endothelial cells and fibroblasts caused by SH045, we performed differential gene expression analysis using a pseudobulk RNA‐Seq approach.^[^ ^22^ ^]^ In endothelial cells, our analysis revealed 11 up‐ and 760 downregulated differentially expressed genes (DEGs) between SH045 and Vehicle group (Table S1 and Figure S8A, Supporting Information). Gene set overrepresentation analysis of upregulated genes in endothelial cells revealed no significantly overrepresented pathways (Figure S8B, Supporting Information). The 760 downregulated DEGs were grouped into 20 umbrella terms of Biological Processes (BP) based on the Gene Ontology (GO). Overall, we identified 36 significantly overrepresented pathways, whereas seven of them could be assigned to a wider umbrella term (mitochondrial ATP synthesis coupled proton transport, ATP synthesis coupled proton transport, mRNA splicing via spliceosome, hydrogen peroxide catabolic process, antigen processing and presentation, positive regulation of lamellipodium assembly, microtubule‐based process, Table S2, Supporting Information). The top10 overrepresented pathways are cytoplasmic translation, mitochondrial ATP synthesis coupled proton transport, translation, ribosomal small subunit assembly, ATP synthesis coupled proton transport, cellular respiration, mitochondrial electron transport, apoptotic process, mitochondrial electron transport, response to oxidative stress (Figure S8C, Supporting Information).

In contrast to endothelial cells, fibroblasts had more upregulated DEGs (125 genes) but less downregulated DEGs in the SH045 group (71 genes) (Table S1 and Figure S8D, Supporting Information). Overrepresentation analysis of upregulated genes in fibroblasts revealed 30 significantly overrepresented pathways, whereas four of them were assigned to the response to mechanical stimulus, regulation of the MAPK cascade, canonical Wnt signaling pathway, and regulation of fibroblast proliferation (Table S2, Supporting Information). The top 10 upregulated BP pathways and GO umbrella terms (Figure S8E, Supporting Information) are extracellular matrix organization, collagen fibril organization, lung development, cell adhesion, negative regulation of cell proliferation, blood vessel development, endodermal cell differentiation, cellular response to transforming growth factor beta, cell migration, osteoblast differentiation. For the downregulated DEGs within the fibroblasts three pathways have been identified as significantly overrepresented including immune system process, antigen processing and presentation, and inner ear development (Table S2 and Figure S8F, Supporting Information). Genes involved in the inner ear development pathway included *C1qb*, *Tgfb1*, *H2‐k1*, *Igfbp7*, and *Cxcl14*, all of which are associated with the regulation of inflammatory response, fibrosis, cell proliferation, and apoptosis.

Apart from endothelial cells and fibroblasts, various cell types in the SH045 group exhibited DEGs. Tubule and inflammatory cells exhibited the greatest number of DEGs among the major cell types analyzed. Similar to endothelial and fibroblast populations, proximal tubular cells exhibited downregulation of genes associated with reactive oxygen species (ROS) metabolism, suggesting a protective response against tubular injury (Figure S9, Supporting Information). Among the inflammatory cell populations, macrophages emerged as the most significantly impacted subset (Figure S10 and Table S1, Supporting Information).

### 2.3 SH045 Changes the Relative Abundance of Endothelial Cells and Fibroblasts

**Figure 3:**
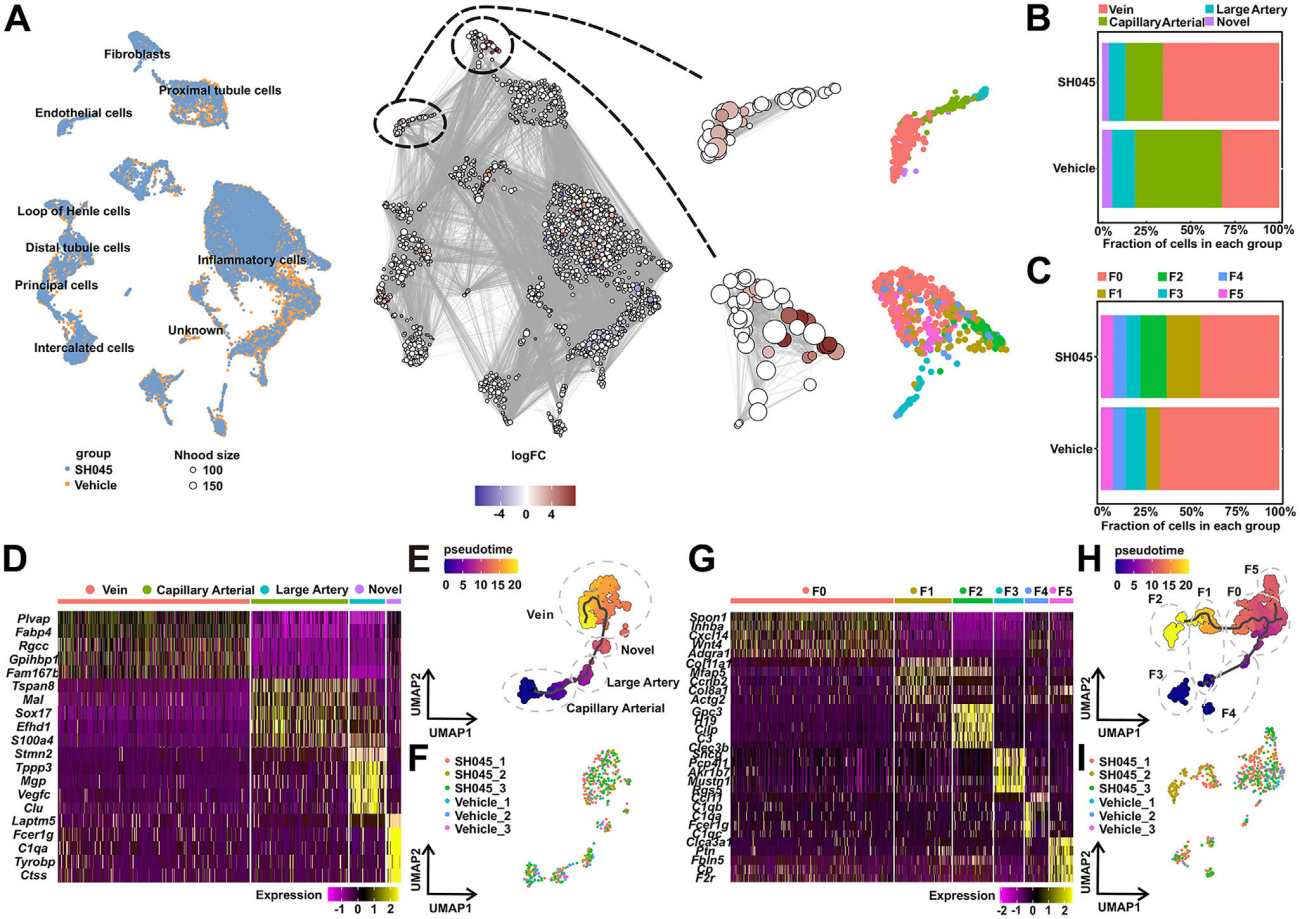
Characterization of renal endothelial cells and fibroblasts in kidneys isolated from SH045 and vehicle treated mice. A) A neighborhood graph of the results from Milo differential abundance testing. Nodes are neighborhoods, colored by their log fold change between SH045 and Vehicle group. Non‐differential abundance neighborhoods (FDR 10%) are colored white. Size of circles represents the number of cells in each neighborhood. The region encircled by the dashed line denotes neighborhood groups that correspond to the endothelial cell (upper panel) and the fibroblast (lower panel) subpopulations. UMAP and stacked bar plots showing different cell types and clusters of endothelial cells B) and fibroblasts C) from SH045 and Vehicle. D) Heatmap of top 5 marker genes in subtypes of endothelial cells. E) Ordering renal endothelial cells along Monocle3 pseudotime trajectory. F) UMAP of endothelial cells representing individual samples. G) Heatmap of top 5 marker genes in subtypes of renal fibroblasts. H) Ordering renal fibroblasts along Monocle3 pseudotime trajectory. I) UMAP of fibroblasts representing individual samples.

To better understand the role of endothelial cells in the immune cell infiltration, we performed a separate sub‐clustering for endothelial cells to get finer‐grained sub‐clusters based on the scRNA‐Seq atlas of endothelial cells.^[^ ^23^ ^]^ This led to the identification of four different sub‐types of endothelial cells in SH045 and Vehicle groups (**Figure** **3**). Upon analysis of the top 50 marker genes in each cluster, we found that 13 (26%) genes in cluster 0 corresponded to marker genes of vein endothelial cells. In clusters 1 and 2, 19 (38%) and 12 (24%) genes, respectively, the data were consistent with marker genes of capillary artery endothelial cells. Cluster 3 exhibited 12 (24%) genes consistent with marker genes of large artery endothelial cells. However, in cluster 4 (17 cells), no marker genes matched any endothelial cell type (Table S3, Supporting Information). Furthermore, in cluster 4 endothelial cells appeared only in UUO subjected kidneys and were consequently designated as a novel category. To investigate the existence of this cell subpopulation in human kidneys, we reexamined single‐cell RNA sequencing data from the CKD atlas as reported by Kuppe et al.^[^ ^24^ ^]^ Our analysis identified a unique cluster of endothelial cells, characterized by marker genes typical of newly discovered endothelial cells. This cluster showed a robust correlation with the identified novel group of endothelial cells, indicating the presence of this endothelial cell subtype in human kidneys from patients with CKD, particularly in diabetic kidney disease (DKD) (Figures S11 and S12, Supporting Information). By assigning cells to partially overlapping neighborhoods on the k‐nearest neighbor graph using Milo algorithm, we found that SH045 increased abundance of vein endothelial cells (**Figure** **3**A,B).

Next, we used the same approach to identify sub‐clusters of fibroblasts. We used F0 to F5 to name different fibroblast clusters (Figure 1E). Based on a comparison with a kidney fibroblast atlas,^[^ ^25^ ^]^ the identified subpopulations F0, F1, F4, and F5 likely represent myofibroblasts, while F2 appears to correspond to fibroblasts, and F3 includes vascular smooth muscle cells or pericytes (Figure S13, Supporting Information). Interestingly, F2 subcluster (61 cells) emerged exclusively in the SH045 group, and F0 abundance declined in the SH045 group (Figure 3A,C). The top five genes separating the endothelial sub‐types confirmed these results by demonstrating a high degree of similar expression for *Tspan8*, *Sox17*, and *S100a4* between capillary and large artery cells (Figure 3D). The pseudotime trajectory analysis of the endothelial sub‐clusters based on their expression profile showed a dynamic transition between capillary arterial and vein endothelial cells. The novel endothelial cell population is located between the clusters of large artery and vein endothelial cells and connected to them (Figure 3E,F). To understand the transcriptome differences between novel endothelial cells and other three identified sub‐types, we extracted the marker genes and performed overrepresentation analysis. In total, 151 marker genes in the novel endothelial cells were predominantly assigned to BP pathways related to immune system process, innate immune response, immune response, inflammatory response, positive regulation of tumor necrosis factor production, positive regulation of interleukin−6 production, chemotaxis, antigen processing and presentation of exogenous peptide antigen, positive regulation of phagocytosis (Figure S14 and Table S4, Supporting Information). Although endothelial cells exhibited reduced expression of genes related to mitochondrial ATP synthesis, the novel endothelial subpopulation maintained glycolytic activity and fatty acid oxidation (FAO) at levels comparable to other endothelial subtype (Figure S15A–C, Supporting Information). Furthermore, our analysis demonstrated a weak positive correlation between glycolysis and FAO (r = 0.2) in endothelial cells. (Figure S15D, Supporting Information). This suggests presence of slightly more metabolically active cells unitizing both FAO and glycolysis. Among the top five gene expression markers in fibroblast subpopulations, F1 and F2 exhibit common expression of *Mfap5*, *Col11a1*, and *Col8a1*, suggesting that F2 subtype might have been evolved from F1 cells (Figure 3F). Pseudotime analysis on the six fibroblast clusters showed that this SH045 specific F2 cluster is connected to F1, thereby suggesting an endpoint in the fibroblast trajectory (Figure 3G). Furthermore, F5 and F0 exhibit a close relationship, with F5 appearing as an additional branch point of F0. F0 is connected to F3 and F4 without branching toward these two subtypes (Figure 3H,I). Similarly, to endothelial cell we extracted all marker genes and performed overrepresentation analysis. In total, 109 marker genes in the F2 fibroblasts were predominantly assigned to BP pathways related to cell adhesion, extracellular matrix organization, canonical Wnt signaling pathway (Figure S16A and Table S4, Supporting Information).

### 2.4 Molecular Mapping of Cell Types Resolved in Space

**Figure 4:**
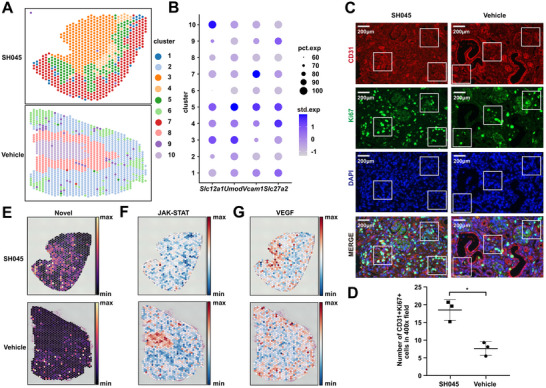
Resolving spatial distribution of novel endothelial cells and pathways activity in a mouse UUO model. A) Clustering assignment for SH045 (left panel) and Vehicle (right panel) group tissue sections. B) Dot plot showing the expression of marker genes representing typical kidney structures in clusters identified by PRECAST algorithm. The size of the dot indicates the percentage of positive spots, and the color indicates the average expression. *Slc27a2* (solute carrier family 27 member 2) and *Vcam1* (vascular cell adhesion molecule 1) represent renal medulla, *Slc12a1* (solute carrier family 12 member 1) and *Umod* (uromodulin) represent renal cortex. pct.exp, percentage expression; std.exp, standardized expression. C) Representative immunofluorescence staining images of CD31 and Ki67 colocalization in SH045 (left panel) and Vehicle (right panel) kidneys (magnification: 400x, scale bar: 200 µm). D) Quantification of CD31+Ki67+ cells in kidney sections from SH045 and Vehicle treated mice. (SH045 n = 3, Vehicle n = 3). Data expressed as mean ± SEM. Mann‐Whitney U‐test was used. ** *p* < 0.01. E) Distribution of novel endothelial cells in samples from SH045 (upper panel) and Vehicle (lower panel) group. Activity of JAK‐STAT F) and VEGF G) pathways in SH045 (upper panel) and Vehicle (lower panel) group.

Next, we performed spatial transcriptome analysis to study tubulointerstitial mechanisms of fibrosis, cell‐specific information, and cell‐specific interactions in the spatial context. The spatial transcriptomics datasets contained a total of 2000 spots (1210 spots for Vehicle and 790 spots for SH045 group, and on average 5395 genes per spot). Following quality control (Figure S17, Supporting Information), unsupervised spatial clustering revealed 10 distinct clusters (**Figure** **4**A). Through the identification of specific marker genes for key kidney structures, we determined that clusters 3 and 10 correspond to the renal medulla, while clusters 7 and 2 are associated with the renal cortex (Figure 4B). Other clusters may represent an overlapping zone. Overall, the spatial data clearly demonstrate typical zones of biological processes in the kidney (cortex, medulla regions). We deconvoluted each spot on the basis of the annotated scRNA‐seq data from the same sample (Figure S18, Supporting Information). In line with scRNA‐seq data, the SH045 group displayed more spots containing higher proportion of endothelial cells (Figure S18A, Supporting Information). Next, we validated the results at the protein level by using immunofluorescence staining. Data revealed higher expression levels of CD31 and Ki67 in SH045 (Figure 4C,D) group, indicating increased endothelial cell proliferation. The 2m post I/R model recapitulated similar outcomes, supporting the consistency of the observed effects (Figure S19, Supporting Information).

Novel endothelial cells were localized in the medulla and cortex of SH045 treated kidney and in predominantly in the cortex of Vehicle treated kidney (Figure 4E). Immunofluorescence analysis revealed this endothelial subpopulation in both UUO and 2‐month post–I/R kidneys, supporting its relevance across models of renal fibrosis (Figure S20, Supporting Information). Next, we determined signaling pathway activities using PROGENy database for each spot from the spatial gene expression data. Spatially localized pathway activities with the estimated cellular abundance per spot linked the information on spatial cell composition to cellular function for each slide. The Janus family tyrosine kinase‐signal transducer and activator of transcription (JAK‐STAT) pathway exhibited relatively lower activity in medulla of SH045 sample, which was enriched in novel endothelial cells (Figure 4F). Of note, decreased inflammatory cell abundance occurred in this area. The presence of novel endothelial cells was associated with the activity of vascular endothelial growth factor (VEGF) pathway, displaying higher activity in regions enriched with novel endothelial cells in SH045 group (Figure 4G). In contrast, the Vehicle group lacked the VEGF pathway activity in the medulla and was associated with higher abundance of inflammatory cells. The presence of ECRIN correlated with lower *Fn1* expression in spatially resolved analyses, indicative of reduced ECM accumulation (Figure S21, Supporting Information).

### 2.5 Molecular Characteristics of F2 Fibroblast Subtype

**Figure 5:**
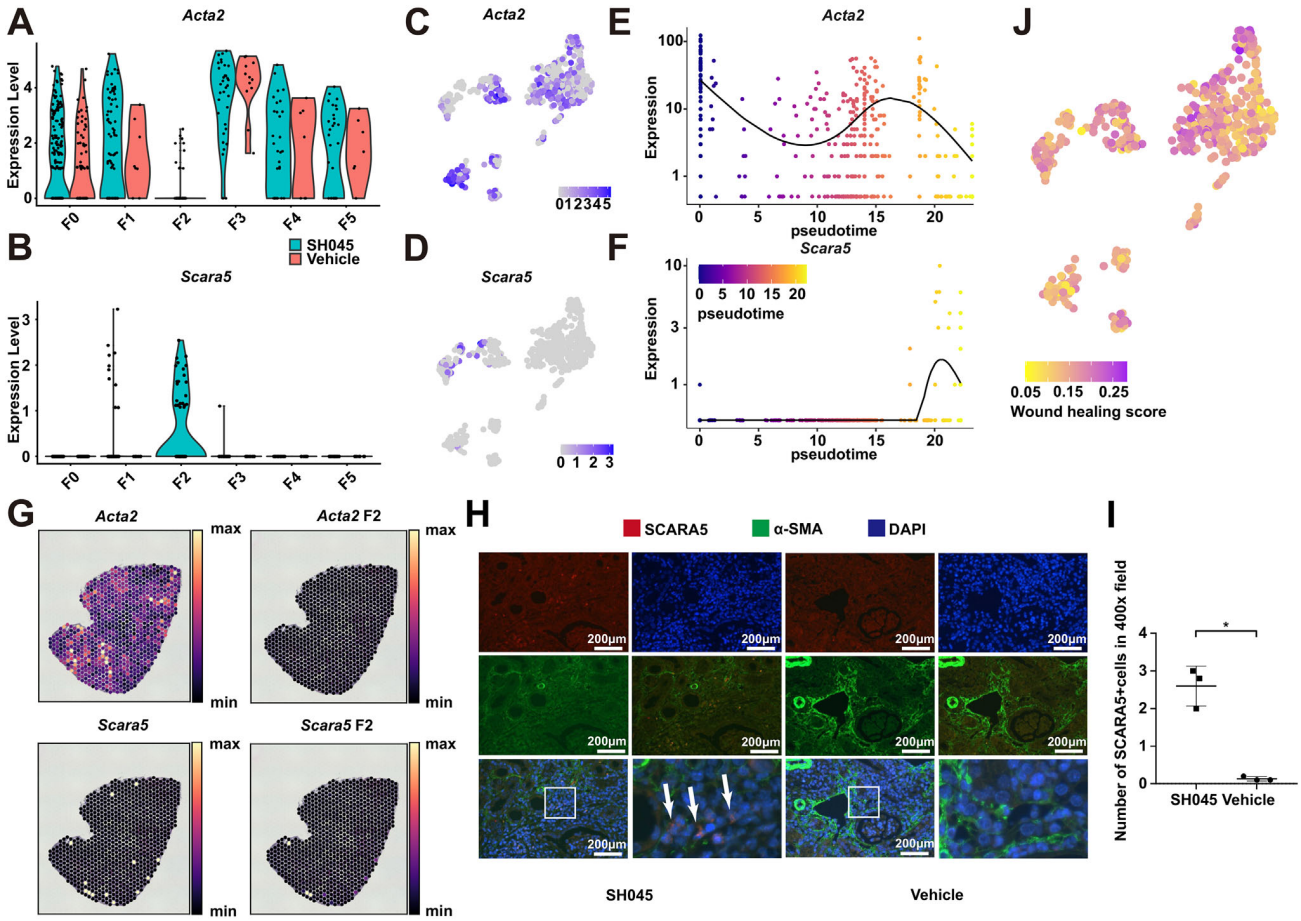
*Acta2* and *Scara5* expression in the fibroblasts using scRNA‐Seq, spatial transcriptomics, and immunofluorescence. Violin plots A,B) and UMAP plots C,D) showing expression of *Acta2* and *Scara5* in fibroblast subpopulation. Pseudotime trajectory analysis display expression changes of *Acta2* E) and *Scara5* F). G) Spatial expression of *Acta2* and *Scara5* in SH045. H) Representative immunofluorescence staining images of SCARA5 and α‐SMA colocalization in kidney sections from SH045 (left panel) and Vehicle (right panel) treated mice (magnification: 400x, scale bar: 200 µm). I) Quantification of SCARA5+ cells in kidney sections from SH045 and Vehicle treated mice. (SH045 n = 3, Vehicle n = 3). Data expressed as mean ± SEM. Mann‐Whitney U‐test was used. ** *p* < 0.01. J) UMAP plots showing relative level of wound healing score in each fibroblast subpopulation.

*Acta2* is one of the markers for the myofibroblasts that play a crucial role in the kidney fibrosis.^[^ ^24^ ^]^ Myofibroblast‐proliferation is associated with further deterioration of kidney function. Pseudo‐bulk RNA‐Seq showed that *Acta2* expression was similar between SH045 and Vehicle groups in F0, F1, F3, F4, F5 subtypes. However, in F2 *Acta2* expression was much lower compared to other fibroblasts subpopulations (**Figure** **5**A,C). Therefore, we hypothesized that F2 represent a distinct subtype from myofibroblast cell type. *Scara5* was reported as a marker for myofibroblast progenitors in the human kidney.^[^ ^24^ ^]^ Interestingly, *Scara5* is almost exclusively expressed in F2 (Figure 5B,D; Figure S22, Supporting Information). The expression patterns of these two genes displayed divergent trajectories along the pseudotime continuum, supporting the idea that F2 likely represents an inactive myofibroblast phenotype (Figure 5E,F). In line with this evidence, spatial expression patterns showed no expression *Acta2* in F2, while *Scara5* was expressed (Figure 5G). Immunofluorescence confirmed SCARA5 expression in the SH045 samples without α‐SMA co‐localization (Figure 5H,I). Of note, the diminished number of SCARA5‐positive cells was associated with advanced CKD stage (Figure S23A,B, Supporting Information). In the 2m post I/R model, SH045 administration enhanced the population of Scara5‐positive cells in the kidney to levels higher than those seen in the UUO model (Figure S23C,D, Supporting Information).

Next, using Molecular Signatures Database (MSigDB) we calculated wound healing score for identified fibroblast subpopulations (Figure 5J). Similar to F0, F2 wound healing score was high, suggesting that F2 mediate reparative processes in the kidney upon SH045 treatment.

**Figure 6:**
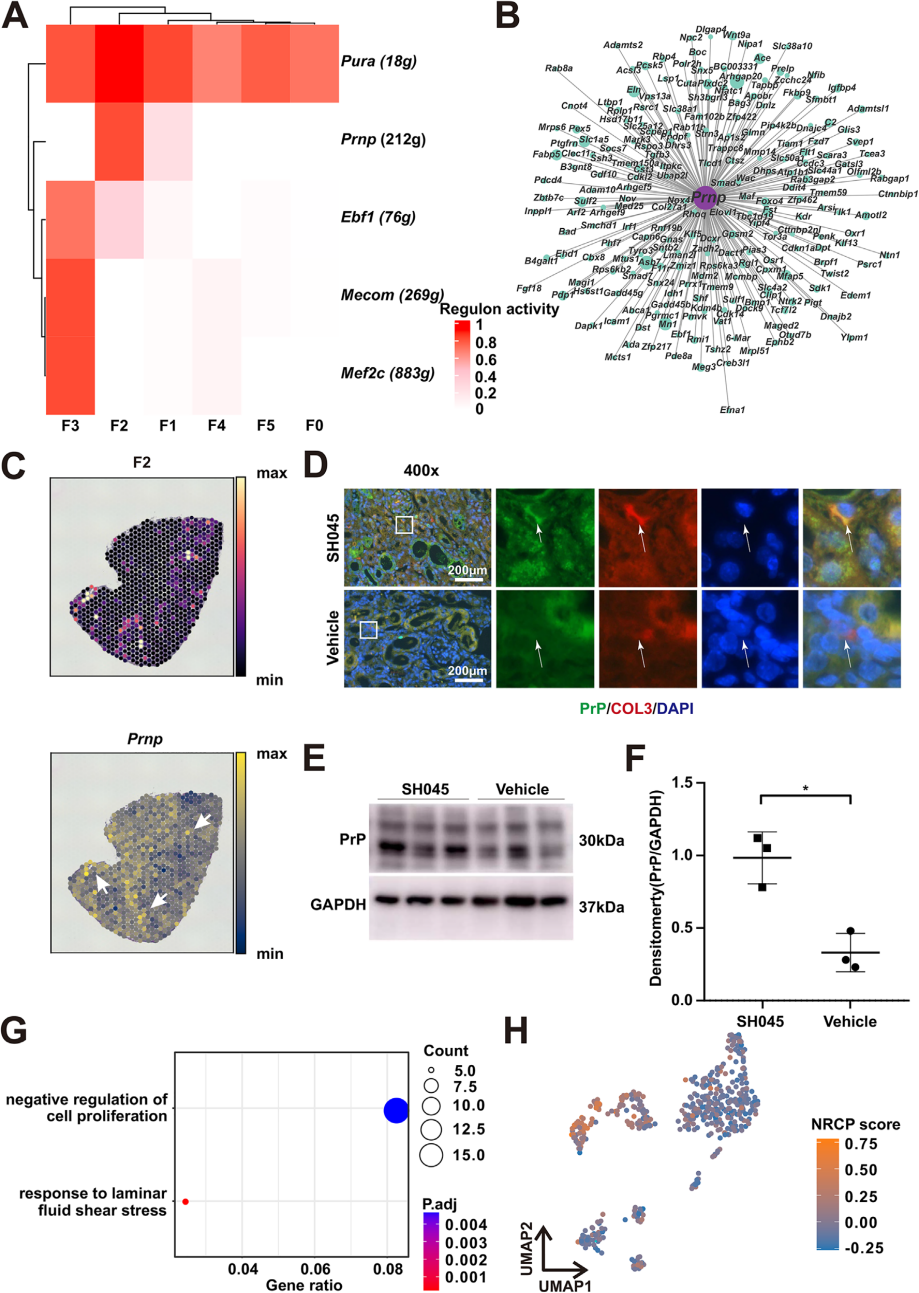
Transcriptional regulatory network in F2 fibroblasts. A) Heatmap of top transcription factors activities in each fibroblast subpopulation. B) Gene network showing target genes of *Prnp*. The dot size represents relative weight values in the regulatory network. C) Spatial distribution of F2 fibroblasts (left panel) and spatial activity of *Prnp* transcription factor (right panel) in SH045 group, white arrows represent areas where spots of high *Prnp* activity overlap with the distribution of F2 fibroblasts. D) Representative immunofluorescence staining images of PrP and Collagen III colocalization of kidneys from SH045 (upper panel) and Vehicle (lower panel) treated mice (magnification: 400x, scale bar: 200 µm). E) Representative Western blot of and relative densitometric graphs of GAPDH, PrP in SH045 and Vehicle treated mice. F) Quantification of PrP expression normalized to GAPDH levels. (SH045 n = 3, Vehicle n = 3). Mann‐Whitney U‐test was used. ** *p* < 0.01. G) Enriched Gene ontology (GO) terms of *Prnp* target genes. UMAP plots H) showing relative level of negative regulation of cell proliferation (NRCP) score in each fibroblast subpopulation. *Pura*: purine‐rich element binding protein alpha, *Prnp*/PrP: prion protein (Kanno blood group), *Ebf1*: early B‐cell factor 1, *Mecom*: MDS1 and EVI1 complex locus, *Mef2c*: myocyte‐specific enhancer factor 2C, Col3: Collagen III, GAPDH: glyceraldehyde‐3‐phosphate dehydrogenase 1.

To detect possible transcriptional regulatory network responsible for the SH045 induced fibroblast transformation, we used single‐cell regulatory network inference and clustering analysis. Six different transcriptional factors turned to be activated in the fibroblast (**Figure** **6**A). Notably, the *Prnp (*prion protein PrP) transcription factor regulon, an assemblage comprising 212 gene targets, was enriched within the F2 subtype (Figure 6B; Figure S16B–D, Supporting Information). The spatial distribution of F2 fibroblasts matched the activity of the *Prnp* transcription factor (Figure 6C). PrP protein expression in cells from SH045 treated animals was colocalized with collagen III (Figure 6D). Similarly, quantitative Western blot analysis revealed elevated levels of PrP protein in the kidneys of 2m post I/R mice treated with SH045 (Figure 6E,F; Figure S24, Supporting Information). Functional annotation of this target gene set revealed enriched negative regulation of cellular proliferation (NRCP) (Figure 6G). NRCP score was high in F2 (Figure 6H). This suggests a universal mechanism in which the TRPC6 blocker SH045 orchestrates fibroblast differentiation through the *Prnp* transcription factor. TRPC6 downstream target genes, e.g., (Calcineurin/Nuclear factor of activated T cells (NFAT)) were expressed in fibroblast including F2 (Figure S25, Supporting Information). Our results imply that the conventional TRPC6 signaling cascade remains functionally intact in the F2 cells.

### 2.6 SH045 Stimulates Cell‐Cell Interactions

**Figure 7:**
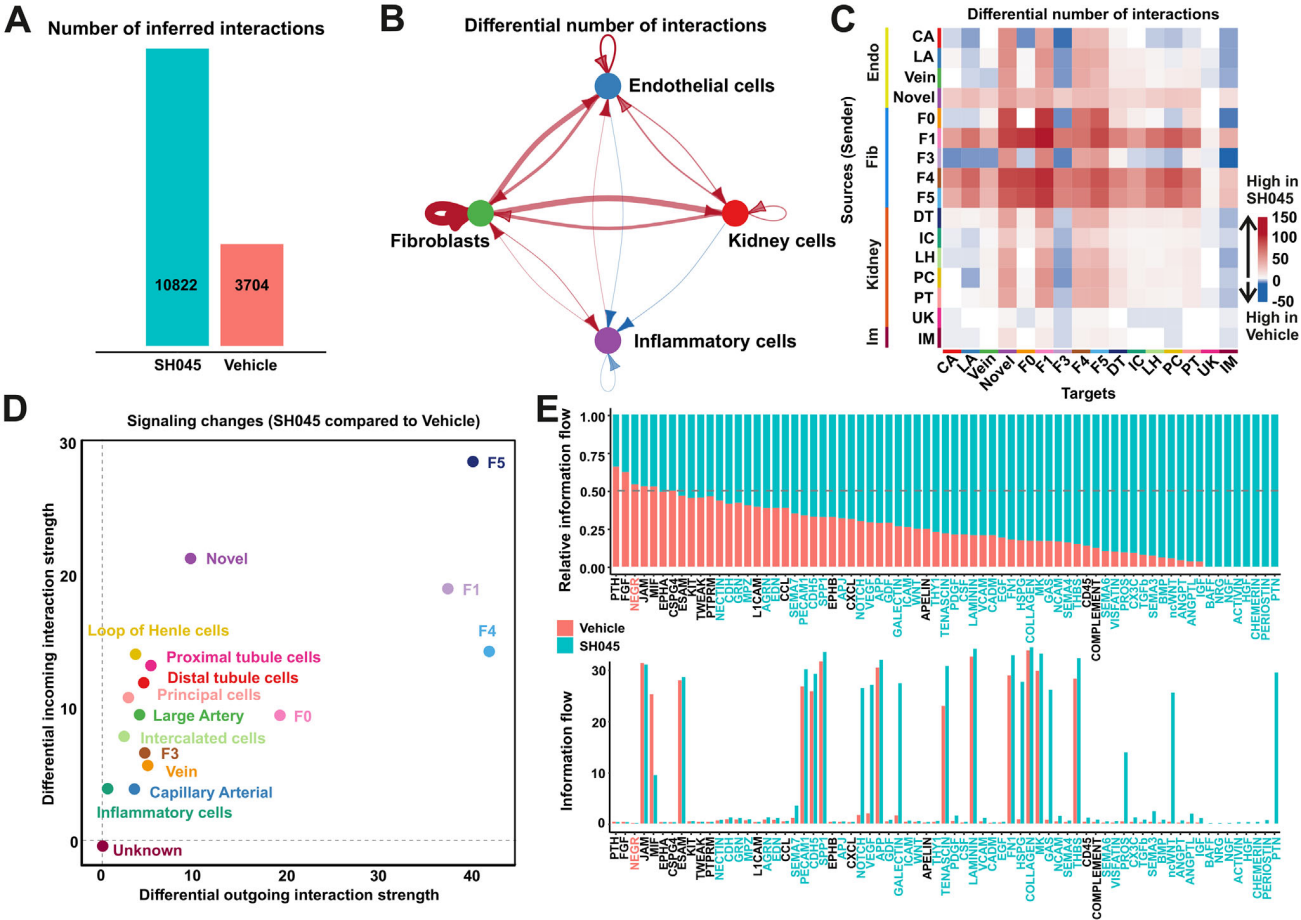
Alterations in network structure and signaling strength of putative cell‐cell communications in kidneys isolated from SH045 and Vehicle treated mice. A) Total number of possible interactions. B) Differential number of possible interactions between the four major kidney cell types. Red and blue lines indicate higher or lower number of predicted interactions in SH045 and vehicle group, respectively. C) Differential number of possible interactions between any two cell populations. Red (positive values) and blue (negative values) in the color bar indicate higher number of predicted interactions in SH045 versus Vehicle group, respectively. D) Differential interaction analysis identifying prominently altered signaling sources and targets. E) Significant signaling pathways were ranked based on their differences in relative information flow (upper panel) and absolute information flow (lower panel). Differences were calculated by summarizing all communication probabilities in each inferred network. Those colored red and green are more enriched in SH045 and Vehicle groups, respectively. LA, Large Artery; CA, Capillary Artery; DT, Distal tubule cells; IC, Intercalated cells; LH, Loop of Henle cells; PC, Principal cells; PT, Proximal tubule cells; UK, Unknown; IM, Inflammatory cells, F0‐F5 subpopulations of renal fibroblasts.

To assess cell‐cell communication changes related to the gene expression changes between SH045 and Vehicle groups, we employed CellChat based on a database of experimentally proved ligand‐receptor interactions ^[^ ^26^, ^27^ ^]^ Our analysis revealed a dramatic increase in the total number of intercellular interactions in the SH045 group (**Figure** **7**A). There was a marked increase in putative signaling within and between the endothelial cells, fibroblasts, and kidney cell populations (Figure 7B). The number of communications from endothelial cells to fibroblasts increased by 195.4% (from 260 to 768), while the number of communications from fibroblasts to endothelial cells increased by 297.3% (from 296 to 1176) compared to the Vehicle cell types. Moreover, there was an increase in the number of communications from fibroblasts to kidney cells (219.2%, from 478 to 1526) and from kidney cells to fibroblasts (150.4%, from 367 to 919). Additionally, the number of communications from endothelial cells to kidney cells increased by 35.2% (from 429 to 580), and the number of communications from kidney cells to endothelial cells increased by 56.6% (from 401 to 628). In contrast, we observed a decrease in interactions between the endothelial and inflammatory cells upon SH045 treatment (17.6% (from 51 to 42) from endothelial to inflammatory cells) and 30.6% (from 85 to 59) from kidney to inflammatory cells.

Besides investigating communication of major cell types, we took a closer look at the subpopulations of endothelial cells and fibroblasts. Our analysis suggests that the novel subpopulation of endothelial cells, as well as F1, F4, and F5 fibroblasts, had more interactions with other subpopulations after SH045 application (Figure 7C). In contrast, F3 displayed less interactions. Notably, the novel endothelial cells, F1, F4, F5 fibroblasts represent both signals’ sources and receivers. To incorporate spatial information into the cell communication analysis, we used node‐centric expression models (NCEM).^[^ ^28^ ^]^ Following the administration of SH045, the majority of endothelial and fibroblast subpopulations demonstrated an increased propensity to both receive and transmit a broader spectrum of cell signals, particularly noticeable in the novel endothelial cell population (Figure S26, Supporting Information). In addition, various inflammatory cell subpopulations showed more interactions with novel endothelial cells and F2 fibroblasts. However, these inflammatory cells showed less communication with F3 fibroblasts (Figure S27, Supporting Information).

We then utilized network centrality analysis. This approach calculates the outgoing and incoming signaling strength of each cell subpopulation to determine their interaction likelihood as signaling sources and targets. Our analysis shows that after the application of SH045, the interaction strength of most cell types increased, with novel endothelial cells and fibroblasts belonging to F1, F4, and F5 exhibiting most striking changes. In these subpopulations, both outgoing and incoming signaling strength drastically increases (Figure 7D).

Moreover, we investigated the information flow for specific signaling network pathways in the SH045 versus Vehicle treated mice. The information flow was defined as the sum of communication probabilities between all cell population pairs in the inferred network. Both relative and absolute information flow through certain pathways, e.g., non‐canonical (nc)WNT, GAS and VEGF, significantly increased in the SH045 group (Figure 7E).

We observed a marked increase in intercellular communication in the Vehicle group compared to the control group (Figure S28A, Supporting Information). Fibroblasts were the prominent cell type mostly affected by UUO exhibiting the highest communication intensity, both in terms of incoming and outgoing signals (Figure S28B,C, Supporting Information). Information flow chart reflects the ongoing fibrosis and inflammation after UUO through different signaling pathways, e.g., FN1, Laminin, SPP1 and collagen (Figure S28D, Supporting Information).

**Figure 8:**
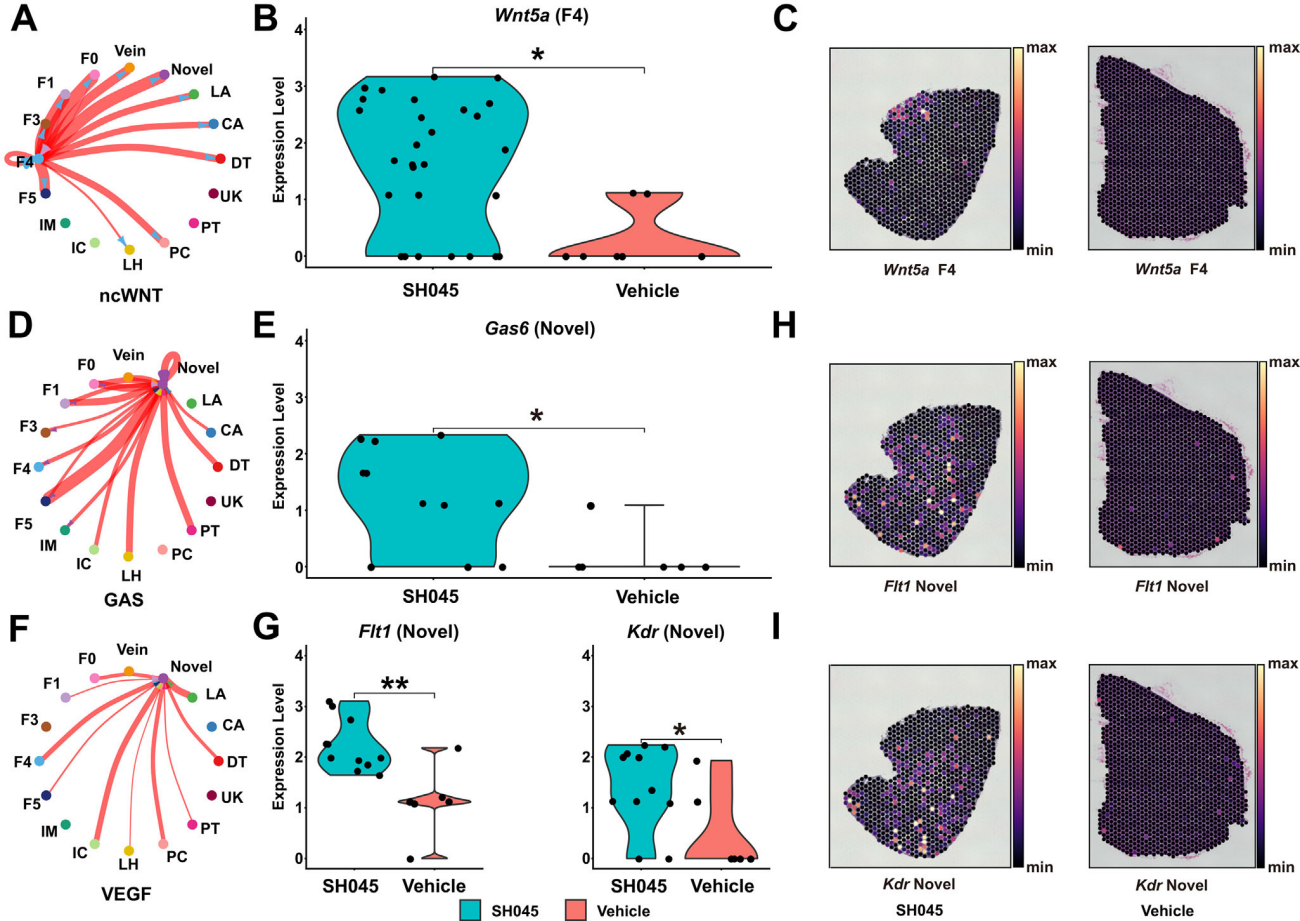
Signaling pathways and key genes enriched by SH045 treatment. A) Circle diagram of signals sent from and received by F4 fibroblasts in noncanonical (nc) WNT signaling pathway network. Differential number of possible interactions between the four major kidney cell types. B) Expression of key gene *Wnt5a* of ncWNT signaling network in F4 renal fibroblasts. C) Spatial expression level of *Wnt5a* in F4 fibroblasts from SH045 (left panel) and Vehicle (right panel) group. D) Circle diagram of signals sent from and received by Novel renal endothelial cells in GAS signaling pathway network. E) Expression of key gene gamma‐carboxyglutamic acid (Gla)‐containing protein gene (*Gas6*) of GAS signaling network in Novel renal endothelial cells. F) Circle diagram of signals sent from and received by Novel endothelial cells in Vascular endothelial growth factor (VEGF) signaling pathway network. G) Expression differences of key genes (*Flt1* and *Kdr*) of VEGF signaling network in Novel renal endothelial cells. The thickness of the lines indicates the relative number of cell interactions. Red lines indicate higher number of predicted interactions in SH045 group. Spatial expression level of *Flt1* H) and *Kdr* I) in Novel endothelial cells from SH045 (left panel) and Vehicle (right panel) group. LA, Large Artery; CA, Capillary Artery; DT, Distal tubule cells; IC, Intercalated cells; LH, Loop of Henle cells; PC, Principal cells; PT, Proximal tubule cells; UK, Unknown; IM, Inflammatory cells, F0‐F5 subpopulations of renal fibroblasts. * *p* < 0.05, ** *p* < 0.01.

To identify cell populations responsible for increase in the information flow due to SH045 application, we closer examined corresponding signaling pathway networks. In the Vehicle group, only F0 fibroblasts initially transmitted some signals through the ncWNT pathway (Figure S29A, Supporting Information). However, after SH045 treatment, both F1 and F4 fibroblasts gained substantial ncWNT signaling activity (Figure S29A, Supporting Information). F4 fibroblasts exhibited strong signaling capability to all endothelial cell subtypes and fibroblasts, but all of these signals were absent in Vehicle group (**Figure** **8**A). The activation of ncWNT network by SH045 mainly occurs due to elevated *Wnt5a* expression in F4 fibroblasts (number of cells in the SH045 group and the Vehicle group were n = 29 and n = 7, respectively) (Figure 8B). The high expression of *Wnt5a* in SH045 sample F4 fibroblasts was confirmed at spatial resolution (Figure 8C).

Although the communication probability trough GAS pathway increased in a vast number of cell types, the novel endothelial cells were predominantly affected (Figure 8D; Figure S29B, Supporting Information). Of note, the novel endothelial cells transmit signals to all fibroblast subpopulations and receive signals from most cell types. In contrast, the novel endothelial cells neither send nor receive any signals in Vehicle group (Figure 8D). Furthermore, the expression level of *Gas6* gene is substantially upregulated in these cells in the SH045 group (n = 11) compared to the Vehicle group (n = 6) (Figure 8E).

In the VEGF pathway, signals originate primarily from kidney cells and F3 fibroblasts in the Vehicle group. However, after SH045 treatment VEGF signaling from other cells to the novel endothelial cells became prominent (Figure 8F; Figure S29C; Supporting Information). As the primary recipients of signals, the expressions of *Flt1* and *Kdr* genes as receptors were significantly upregulated in the novel endothelial cells of the SH045 group (n = 11) compared with the Vehicle group (n = 6) (Figure 8G). In line with this evidence, both genes were expressed in novel endothelial cells from the SH045 group in spatial transcriptomic experiment. However, no expression of these genes was detected in the novel endothelial cells of Vehicle group. (Figure 8H,I).

## 3 Discussion

**Figure 9:**
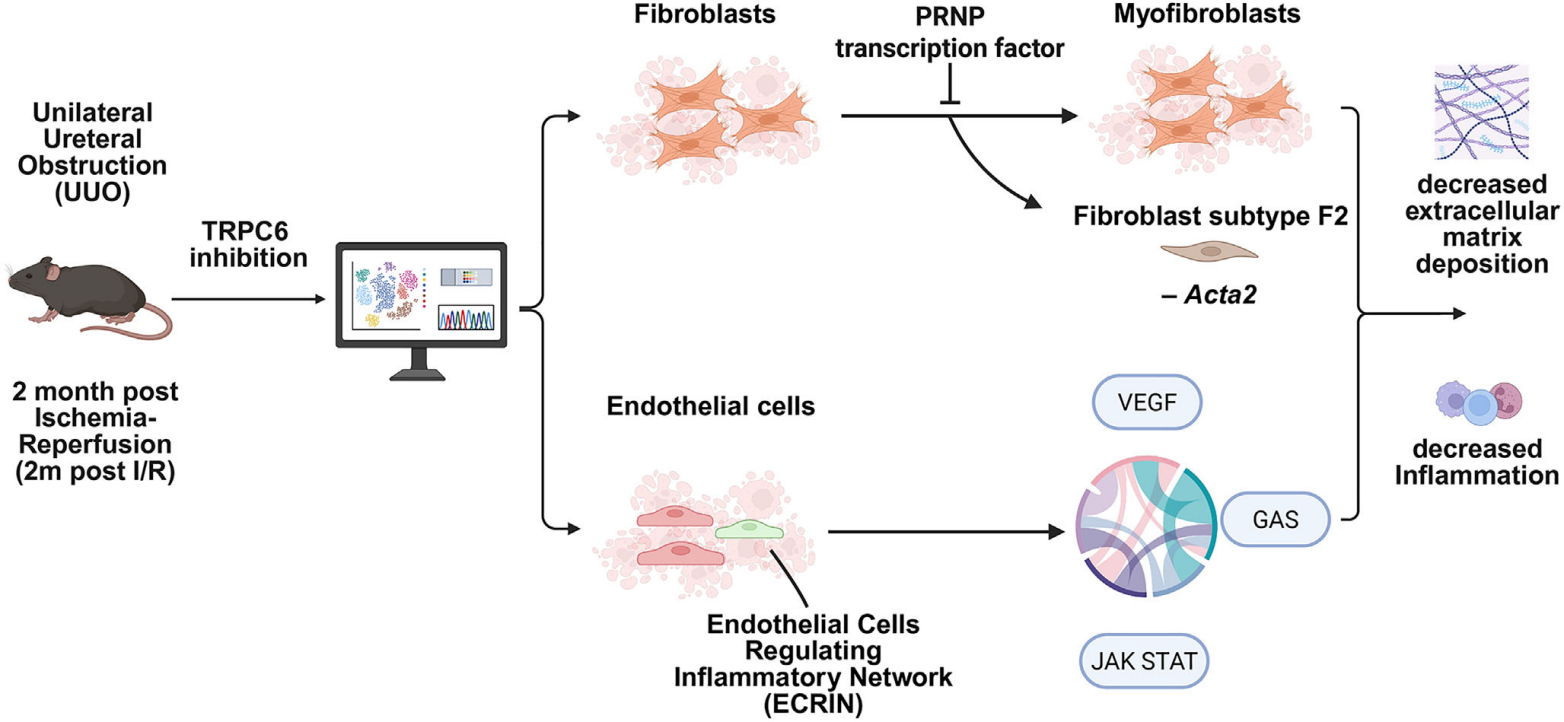
Schematic illustration of the mechanism by which TRPC6 blockade improves renal fibrosis.

Recent advancements in scRNA‐Seq technology have made it a comprehensive tool for uncovering the mechanisms of various diseases, including kidney disease.^[^ ^7^, ^29^, ^30^, ^31^ ^]^ Our previous studies showed that, TRPC6 inhibition (by SH045 and genetic ablation) it ameliorates renal fibrosis and immune cell infiltration in the UUO model^[^ ^12^, ^13^ ^]^ and does not appear to play a causal role in human focal segmental glomerulosclerosis (FSGS).^[^ ^32^ ^]^ Nevertheless, the underlying mechanisms through which TRPC6 may mitigate renal fibrosis remain unknown. SH045 altered differentiation trajectory of endothelial cells and fibroblasts. Our data showed a significant increase in both endothelial and fibroblast numbers (63.3% and 75.2%, respectively). We discovered a new endothelial cell subtype, named ECRIN, in the UUO group that was responsive to SH045 treatment. These effects were associated with decreased inflammatory cell infiltration. Moreover, a distinct subtype of fibroblasts (F2) emerged in SH045 treated kidney. These factors could potentially elucidate the anti‐fibrotic effects of SH045 that we previously observed in the UUO model of accelerated fibrosis.^[^ ^12^ ^]^ Moreover, the 2m post I/R model, which more closely recapitulates kidney fibrosis characteristic of CKD, yielded consistent results, reinforcing the robustness and translational relevance of the findings (**Figure** **9**).

The rationale for focusing on endothelial cells and fibroblasts is biologically driven. Endothelial cells are involved in fibroblast activation and inflammation response, while myofibroblasts are directly responsible for producing the ECM excess ^[^ ^33^, ^34^ ^]^ In addition, cell‐cell communication analyses showed that fibroblasts were the prominent cell‐type affected by UUO itself and SH045 application. Following kidney cell types, endothelial cells exhibited the second highest number of differentially expressed genes (DEGs). In in the accelerated fibrosis model (UUO), ≈95% of the total myofibroblast population originates from local resident fibroblasts, differentiated bone marrow cells, and the endothelial‐to‐mesenchymal transition program.^[^ ^2^ ^]^ Given that epithelial‐to‐mesenchymal transition contributes less significantly to fibrosis (5%), we focused primarily on fibroblasts and endothelial cells, which may play key roles in regulating inflammation. The high number of DEGs in kidney cell types, such as proximal tubule cells, likely reflects the impact of SH045 in reducing interstitial fibrosis and inflammation.

Given the kidney's high metabolic demand, kidney fibrosis is believed to be closely linked to hypoxia.^[^ ^35^ ^]^ Indeed, a lower vessel density in aged mice is associated with accelerated renal fibrosis.^[^ ^36^ ^]^ Increased VEGF signaling has been shown to prevent age‐associated capillary loss, improve organ perfusion and function, and extend life span.^[^ ^37^ ^]^ Notably, we found an increase in vascular endothelial cells after TRPC6 blockade. This implies that SH045, by controlling endothelial function, might modulate the renal oxygen supply, potentially contributing to its anti‐fibrotic effects. Consistently, oxidative stress and cellular senescence were reduced following TRPC6 treatment.

Consistent with this observation, genes involved in ROS metabolism were downregulated in proximal tubules of the SH045‐treated group. A recent study indicated that tubular damage and atrophy can actively promote a pro‐inflammatory state and sustained myofibroblast recruitment during the AKI‐to‐CKD transition, mediated via VCAM‐1.^[^ ^38^ ^]^ However, our findings may challenge this mechanism in the context of SH045‐induced nephroprotection, as VCAM‐1 expression was paradoxically elevated in the SH045‐treated group (fold change: 0.36) (Table S1, Supporting Information). Thus, the observed changes in the tubular system are most likely driven by SH045‐mediated suppression of inflammation, oxidative stress, and fibrotic processes.

Additionally, we discovered a unique, novel subtype of endothelial cells (ECRIN) that are solely found in kidney fibrosis. SH045 markedly increased cell interactions between novel and different other cell types. Moreover, our analysis showed that the novel endothelial cells (ECRIN) act as signal receivers of the VEGF pathway, with significantly increased expression levels of its both receptors (*Kdr* and *Flt‐1*). We also found that VEGF activity resolved in space was correlated with the distribution of the novel endothelial cells (ECRIN) in SH045 group. Through binding to Kdr and Flt‐1 on endothelial cells, VEGF stimulates their proliferation, migration, and formation of a lumen‐like structure, while also activating downstream pathways such as PI3K/Akt and MAPK/ERK.^[^ ^39^, ^40^ ^]^ A recent study also confirmed the role of promoting angiogenesis in improving recovery from acute kidney injury (AKI) and limiting the progression of CKD.^[^ ^41^ ^]^ In the novel endothelial cells, we noted a high prevalence of GAS pathway signals and elevated Gas6 expression. Previous studies have demonstrated that the GAS pathway exerts anti‐inflammatory effects in multiple organs, such as the lungs, liver, and heart.^[^ ^42^ ^]^ Particularly in the heart, the Gas6/Axl axis is crucial for cardiovascular remodeling, as it promotes vascular smooth muscle cell proliferation, migration, and protection against apoptosis.^[^ ^43^ ^]^ However, it also exhibits pro‐fibrotic properties both in vitro and in vivo.^[^ ^44^, ^45^ ^]^ Its dual impact on both acute and chronic injuries results in beneficial effects on inflammation and fibrosis, characterized by ECM deposition, progressive tissue disorganization, and functional loss.^[^ ^42^ ^]^ The role of the GAS pathway in kidney disease remains debated; some research suggests it protects against ischemia‐reperfusion injury,^[^ ^46^ ^]^ whereas other studies propose that inhibiting the GAS pathway receptor AXL could alleviate renal dysfunction in glomerulonephritis by preventing the EMT of renal tubules.^[^ ^47^ ^]^ In our samples, elevated Gas6 expression in the SH045 group reinforces the renoprotective role of this pathway. Nevertheless, the GAS pathway's function in renal fibrosis is still ambiguous, necessitating further research to ascertain its therapeutic potential.

It is widely accepted that the JAK‐STAT signaling cascade plays a pivotal role in the regulation of diverse biological processes, notably in immune response and inflammation.^[^ ^48^ ^]^ Recent studies have highlighted the role of the JAK‐STAT pathway in diabetic nephropathy, demonstrating its ability to trigger inflammation and oxidative stress, which in turn contributes to kidney damage.^[^ ^49^ ^]^ Our spatial transcriptomic analyses showed an activated JAK‐STAT pathway in the Vehicle group. Notably, this activity was associated with the low abundance of novel endothelial cells. In contrast, the samples from the SH045 group exhibited low and uniform activity levels of the JAK‐STAT pathway. Previous studies have thoroughly identified all elements of the JAK‐STAT pathway in damaged kidneys.^[^ ^50^ ^]^ Particularly, overexpressing JAK2 in podocytes intensified several pathological features in Akita diabetic mice, such as proteinuria, mesangial expansion, glomerular basement membrane thickening, and tubulointerstitial fibrosis.^[^ ^51^ ^]^ The JAK2/3 inhibitor AG490 diminishes inflammation, tubular apoptosis, and interstitial fibrosis in UUO mice, and also reduces proteinuria in diabetic mice.^[^ ^52^ ^]^ These results emphasize the complex role of the JAK‐STAT pathway in kidney damage and underscore the therapeutic potential of targeting this pathway. These findings further support the notion that ECRIN, a novel endothelial subpopulation, modulates inflammatory pathways to attenuate kidney fibrosis, as evidenced by its spatial association with reduced *Fn1* expression and diminished ECM deposition.

Our research reveals the existence of a previously unidentified endothelial cell population in human CKD specimens. This discovery is significant as it highlights the crucial role these cells play in the development of renal inflammation and fibrosis in humans. Importantly, this underscores the dual potential of these cells as both a diagnostic marker and a therapeutic target in the treatment and management of CKD. A recent study has shown the protective effects of an angiopoietin‐2 inhibitor in mice, mirroring our findings.^[^ ^53^ ^]^ The inhibitor decreased apoptosis in endothelial cells and curbed endothelial‐mediated macrophage infiltration. Additionally, disruptions in angiopoietin levels have been linked to kidney failure in humans.^[^ ^53^ ^]^ Consequently, these results position these endothelial cells as a highly promising new target for CKD treatment.

Upon injury or cytokine stimulation, fibroblasts undergo differentiation into myofibroblasts. These cells play a crucial role in tissue repair by secreting ECM. Studies have demonstrated that TRPC6 is instrumental in this process, facilitating the transformation of fibroblasts into myofibroblasts and subsequently promoting scar formation.^[^ ^54^ ^]^ The proposed mechanisms may hinge on the interplay between the influx of calcium ions via TRPC6 channels and the activation of calcineurin.^[^ ^54^ ^]^ Our previous studies have substantiated this evidence in vivo, showing that either genetic deletion or pharmacological inhibition of TRPC6 can ameliorate kidney fibrosis ^[^ ^12^, ^14^ ^]^ Interestingly, our scRNA‐Seq analysis identified an increase in fibroblasts in the SH045 kidney. Fibroblasts are known to exhibit marked interlineage plasticity and phenotypic switching during fibrosis.^[^ ^55^ ^]^ Therefore, it is possible that SH045 inactivates fibroblasts actively producing ECM. In line, we observed switching by our pseudotime analysis demonstrating that newly emerged F2 in the SH045 group have very similar transcriptomic characteristics compared to F1. The immunofluorescence staining validated the existence of F2 subcluster. The overrepresentation analysis of F2 fibroblasts was consistent with known fibroblast functions related to the ECM organization. However, among other subpopulations (e.g., F3) F2 exhibits extremely low *Acta2* (αSMA) expression compared to F3. This suggests that F2 may represent a non‐myofibroblast subtype that regulates ECM secretion. Moreover, SH045 suppressed F3 (myofibroblasts) interactions, which can also underlie the antifibrotic effects of TRPC6 blockade. Interestingly, renal biopsy from CKD patients showed reduced number of SCARA5‐positive cells. This state‐of‐affairs was associated with more severe kidney injury, which suggest that SCARA5 activation by SH045 may have therapeutic benefits in humans.

*Prnp*, the gene encoding the prion protein (PrP), is predominantly expressed in the nervous system. Initially, PrP was considered to be associated with various neurodegenerative diseases.^[^ ^56^ ^]^ Nevertheless, recent research has suggested a role for this gene in the pathogenesis of AKI and CKD.^[^ ^57^ ^]^ PrP has been shown to display an iron reductase activity in the proximal tubule epithelial cells^[^ ^58^ ^]^ to potentially influence iron metabolism – a recently recognized novel target in chronic kidney injury and renal fibrosis.^[^ ^59^ ^]^ However, PrP is implicated in the growth and proliferation of MSCs within the kidney, potentially influencing renal fibrosis.^[^ ^60^, ^61^ ^]^ Our findings indicate that targeting *Prnp* inhibits fibroblast proliferation, thereby mitigating kidney fibrosis. Inhibition of TRPC6 channels triggers a *Prnp* transcription factor regulatory network, which contributes to the alleviation of renal fibrosis. Future research should prioritize the individual roles of these genes, highlighting their potential as novel therapeutic targets for renal fibrosis treatment.

Importantly, administration of SH045 markedly stimulated the ncWNT pathway in F4 fibroblasts. Following the treatment with SH045, we observed enhanced interactions between F4 fibroblasts and various endothelial cells, indicating a potential role of this cell subset in angiogenesis‐related activities. Prior research has identified both canonical and non‐canonical WNT pathways as critical contributors to the development of kidney fibrosis.^[^ ^62^, ^63^ ^]^ *Wnt5a*, a pivotal component of the ncWNT pathway, has the capacity to modulate downstream signaling mechanisms, thereby enhancing angiogenesis. For instance, inhibition of the ncWNT pathway through siRNA technology has demonstrated a reduction in endothelial cell proliferation and angiogenesis.^[^ ^64^ ^]^ These findings underscore the significance of endothelial cells and the intricate interplay among diverse cell types.

TRPC6 blockade (e.g., by SH045) has been suggested to exhibit potential negative effects in humans. However, very recent data show that one defective TRPC6 gene copy is not sufficient to cause FSGS, which underscores the importance of increased rather than reduced calcium influx through TRPC6 for podocyte cell death.^[^ ^32^ ^]^ In this context, pharmacological inhibition of TRPC6 channels (e.g., with SH045) may offer a promising therapeutic approach for chronic kidney disease, potentially mitigating maladaptive responses.^[^ ^65^ ^]^

Future studies should focus on the development of isolation techniques for ECRIN and fibroblast subpopulation F2, to enable detailed characterization of their molecular profiles. This will be essential for elucidating the precise mechanisms underlying TRPC6‐mediated nephroprotection, including the potential release of anti‐fibrotic factors. Additionally, the generation and analysis of endothelial‐specific TRPC6 knockout mice will be critical to directly assess the role of endothelial TRPC6 signaling in modulating kidney fibrosis. These models will also preserve the tissue‐specific microenvironment that is critical for proper ion channel function.^[^ ^66^ ^]^ Clinical trials and subgroup analyses may clarify the efficacy of combining TRPC6 blockade with therapies like SGLT2 inhibitors, potentially offering added benefit for CKD patients.^[^ ^67^ ^]^ Moreover, we propose that future studies combine SH045 with SGLT2 inhibitors to evaluate potential synergistic effects. In addition, single‐cell metabolomics could provide insights into the detailed mechanistic links between these approaches.

Although single‐cell sequencing provides extensive cellular‐level insights, it may not fully capture inter‐individual variability observed in disease models such as fibrosis progression. A key limitation of our study is the relatively small sample size, underscoring the need for future investigations involving larger cohorts to robustly validate these findings. Additionally, future studies should examine the heterogeneity of cellular responses in CKD, including models of diabetic and hypertensive nephropathies, where the efficacy of SH045 remains unexplored. To directly assess ECRIN's role in these contexts, it is crucial to prioritize the development of endothelial‐specific *Trpc6* knockout mice, enabling targeted mechanistic validation in relevant pathophysiological settings.

In conclusion, the present study indicates that inhibiting TRPC6 channels may effectively mitigate fibrosis through the modulation of function of both endothelial cells and fibroblasts. This discovery sheds light on potential signaling pathways and transcriptional regulatory networks at both single‐cell and spatial levels, offering promising new avenues for CKD treatment strategies.

## 4 Experimental Section

### UUO Model

A cohort of nine male C57BL/6J mice was purchased from the Jackson Laboratory. These mice were reared in specific‐pathogen‐free (SPF) conditions. 12‐h light‐dark cycle was maintained. Animals had free access to food (E15430‐047, Ssniff, Soest, Germany) and water. All experiments were carried out in strict accordance with the ARRIVE guidelines.^[^ ^68^ ^]^ Experiments were approved by the Berlin Animal Review Board, Berlin, Germany and followed the restrictions in the Berlin State Office for Health and Social Affairs (LaGeSo, No. G0175/18).

The UUO mouse model was performed as described.^[^ ^12^, ^14^ ^]^ In brief, C57BL/6 mice were anesthetized using 2.2% isoflurane with an air flow rate of ≈350 mL min^−1^. Preemptive analgesia was administered subcutaneously using carprofen (5–10 mg kg^−1^). Throughout the surgery, body temperature was sustained at 37.5 °C and monitored using a temperature controller along with a heating pad (TCAT‐2, Physitemp Instruments, Clifton, NJ, USA). After achieving a state of deep anesthesia, the anterior abdominal skin was shaved, and a midline laparotomy was performed via avascular *linea alba* incision to expose the left ureter. The ureter was then ligated twice close to the renal pelvis using a 5‐0 polyglycolic acid (PGA) suture wire (Resorba, Nürnberg, Germany). The *linea alba* and skin were separately closed, and the wound was sanitized with a silver aluminium spray (Henry Schein, Berlin, Germany). Then, 0.5 mL warm (37 °C) isotonic sodium chloride solution was intraperitoneally injected. Each mouse was subsequently placed in a cage, positioned in front of an infrared (IR) lamp, and monitored until they regained consciousness. For the following 48 h, carprofen (2.5 mg mL^−1^) was added to drinking water (1:50) with a final concentration of 0.05 mg mL^−1^. After the surgery, the mice had unrestricted access to food and water. Seven days after UUO surgery, the mice were euthanized through an overdose of isoflurane and cervical dislocation and perfused with PBS before organ collection. The kidneys from Vehicle (n = 3) and SH045‐treated (n = 3) animals were removed. The control group refers to the healthy kidney on the contralateral side of the UUO subjected mice that were treated with Vehicle (n = 3). A part of kidney (80 mg) was removed and placed to sCelLiVETissue Preservation Solution (Singleron Biotechnologies). Subsequent cell dissociation and scRNA‐Seq analysis were performed. The rest of kidney tissue was placed into Tissue‐Tek (O.C.T.) and was frozen at ‐80 using liquid nitrogen and isopentane.

### 2m Post I/R Model

Renal I/R was induced as previously described.^[^ ^69^ ^]^ In brief, male mice (14–18 weeks of age) were anaesthetized with 2.3% isoflurane in air at a flow rate of 350 ml min^−1^, and received preemptive analgesia with buprenorphine (0.2 mg per 100 g body weight). Surgeries were performed individually to ensure consistent isoflurane exposure. Body temperature was maintained at 37 °C and continuously monitored throughout the procedure. Ischemia was induced by clamping the left renal pedicle with a non‐traumatic aneurysm clip (FE690K, Aesculap, Germany) for 30 min. Reperfusion was confirmed visually, after which the abdominal muscle and skin were closed separately using 5‐0 braided silk sutures. Postoperative care included free access to food and water, as well as subcutaneous administration of 1 ml of warm sterile physiological saline. Sham‐operated mice underwent the same surgical procedure without clamping of the renal pedicle. Two months after reperfusion, mice were euthanized by isoflurane overdose followed by cervical dislocation. Kidney samples were collected for downstream analyses. The animal study protocol was approved by the Ethics Committee of Shanghai General Hospital (IACUC: 2023AW034).

### TRPC6 Inhibitor

The SH045 (Larixyl‐6‐N‐methylcarbamate) was dissolved in DMSO to achieve a final concentration of 0.5%.^[^ ^15^ ^]^ Then, it was further dissolved in a 5% CremophorEL solution along with 0.9% NaCl for intraperitoneal injection (i.p.) as previously described.^[^ ^14^ ^]^ Mice undergoing UUO surgery were administered SH045 (20 mg kg^−1^, i.p.) or Vehicle once daily until day 7 after surgery.^[^ ^14^ ^]^

### Renal Biopsies

Renal biopsy samples were collected by the Department of Pathology, School of Basic Medical Sciences at Fudan University, as described previously.^[^ ^70^ ^]^ The diagnosis was established by immunofluorescence, H&E, PAS, PAS–methenamine silver stainingd, and electron microscopy. The para‐carcinoma kidney tissues were used as control. The Ethical Committees of the School of Basic Medical Sciences, Fudan University (2017‐Y009) approved all protocols. An informed consent was obtained from all patients.

### Kidney Histopathology

Histological assessment was performed as previously reported.^[^ ^71^ ^]^ The kidney tissue was fixed in 10% neutral buffered formalin and then embedded in paraffin. Hematoxylin and eosin (H&E) and Masson trichrome staining was performed using 2 µm paraffin‐embedded sections to quantify the percentage of fibrotic area in kidneys. The slides were digitally scanned at 40x resolution using the 3DHISTECH PANNORAMIC SCAN II (Budapest, Hungary). Images were acquired by the 3DHISTECH software CaseViewer (Budapest, Hungary). In each group, 10 fields of view were randomly selected from each murine kidney sample section under a 400× magnification. Semi‐quantitative renal fibrotic scoring was performed in a blinded manner. The findings in Masson trichrome staining were graded from 0 to 5 according to following scheme: 0, no lesion; 1, less than 10%; 2, 10 – 20%; 3, 20 – 30%; 4, 30 – 40%; 5, more than 50%. All measurements were repeated three times.

### Immunofluorescence Staining

Immunostaining was performed as previously described.^[^ ^12^ ^]^ Briefly, sections of 2 µm thickness of paraffin‐embedded kidneys were fixed with 10% neutral buffered formalin and processed using Leica (Leica Microsystems GmbH, Germany) automatic tissue processor. Following dewaxing and rehydration, sections were heated in citrate buffer for antigen retrieval for further antibody incubation. TRPC6‐, SCARA5‐, α‐SMA‐, F4/80‐, Prion‐, CD31‐, Ki67‐positive staining were detected by immunofluorescence using monoclonal mouse anti‐TRPC6 (Cat# ab105845, Abcam, UK), monoclonal rabbit anti‐α‐Smooth Muscle Actin (Cat#19 245, CST, USA), monoclonal rabbit anti‐F4/80 (Cat# 70 076 T, CST, USA), polyclonal rabbit anti‐SCARA5 (Cat# bs‐17271R, Bioss, China), monoclonal rabbit anti‐Prion Protein (Cat# A18058, ABclonal, China), monoclonal mouse anti‐CD31/PECAM‐1 (Cat# sc‐376764, Santa Cruz, USA) and polyclonal rabbit anti‐Ki67 (Cat# ab 15 580, Abcam, UK). Fluorescent signals were then detected using a fluorescence microscope (Zeiss LSM 800; Carl Zeiss Meditec AG, Jena, Germany). Quantitative analysis was conducted in 10 non‐overlapping randomly chosen fields per kidney section under a 400× magnification.

### Western Blotting

Kidney tissue was harvested and lysed in the RIPA Lysis and Extraction Buffer in the presence of protease inhibitor followed by centrifugation. The lysates were analyzed by immunoblotting as described previously.^[^ ^72^ ^]^ Briefly, 25 µg protein of each sample underwent 10% SDS‐PAGE electrophoresis and was then transferred to a PVDF membrane. Nonspecific binging sites of the membrane were blocked with 5% bovine serum albumin (BSA) in Tris‐buffered saline containing 0.1% Tween. Next, the membrane was incubated with the following primary antibodies: monoclonal rabbit anti‐Prion Protein (Cat# A18058, ABclonal, China) and monoclonal mouse anti‐GAPDH (Cat# 60004‐1‐1 g, Proteintech). ImageJ (V1.52, National Institutes of Health, Bethesda, MD, USA) was used to quantify western blot images. Expression level of target proteins was normalized to that of GAPDH expression level.

### Statistics

Statistical analysis was performed using GraphPad 8.01 software. Mann‐Whitney‐U‐Test used for comparison of two groups, one‐way ANOVA followed by Tukey's multiple comparisons post hoc test or two‐way ANOVA followed by Sidak's multiple comparisons post hoc test were used in case of more than two groups. Data were presented as mean ± standard error of the mean (SEM). *P* values < 0.05 were considered statistically significant.

### Tissue Dissociation and Single Cell Isolation

Tissue was washed with 1x PBS (phosphate‐buffered saline, Gibco, cat. nr. 10010–23) and dissociated with ophthalmic scissors to pieces of 1–2 mm. The pieces were digested in 2 ml sCellLive Tissue Dissociation Solution (Singleron Biotechnologies, cat. nr. 1 190 062) at 37 °C for 15 min in a 15‐ml centrifuge tube (Sarstedt, cat. nr. 62.5544.003) with continuous agitation on a thermal shaker. The state of dissociation was checked at regular intervals under a light microscope. Following digestion, the suspension was filtered using a 40‐µm sterile strainer (Greiner, cat nr. 542 040). The cells were centrifuged at 350xg for 5 min at 4 °C and the cell pellets were resuspended in 1 ml PBS. Cells were stained with a 0.4% w/v solution of Trypan Blue (Gibco, cat nr. 15250‐061) and the cell number and viability were calculated in a hemacytometer under a light microscope.

### Single Cell RNA Sequencing Library Preparation

The single cell RNA‐seq libraries were constructed using (GEXSCOPE Single Cell RNAseq Library Kit, Singleron Biotechnologies, cat nr. 4 161 031) according to manufacturer´s instructions. Briefly, for each library, the concentration of the single‐cell suspension was adjusted to 3 × 105 cells ml^−1^ with PBS and the suspension was loaded onto an SD microfluidic chip to capture 6000 cells. Paramagnetic beads conjugated to oligodT probes that carry a unique molecular identifier (UMI) and a barcode unique to each bead (from the same kit) were loaded, after which the cells were lysed. The beads bound to polyadenylated mRNA were extracted from the chip and reverse transcribed into cDNA at 42 °C for 1.5 h, and the cDNA amplified by PCR. The cDNA was then fragmented and ligated to indexed Illumina adapters. The fragment size distribution of the final amplified library was obtained on an Agilent Fragment Analyzer.

### Library Sequencing

The library concentration was calculated using the Qubit 4.0 fluorometer and the libraries were pooled in an equimolar fashion. The single cell libraries were sequenced on an Illumina NovaSeq 6000 using a 2 × 150‐bp approach to a final depth of 90 GB per library. The reads were demultiplexed according to the multiplexing index sequencing on Illumina's BaseCloud platform.

### Spatial Gene Expression Assay

Frozen kidney samples were imbedded in Optimal Cutting Temperature compound (OCT) and frozen in pre‐cooled isopentane. Embedded tissues were stored at ‐80 °C until cryo‐sectioning. RNA of samples was isolated using QIAGEN RNeasy Kit according to manufacturer's specifications (Purification of total RNA from animal tissues). To determine RNA quality and integrity number (RIN value), Agilent RNA 6000 nano/pico kit and Agilent 2100 Bioanalyzer l were used according to the manufacturer (Agilent RNA 6000 nano/pico assay). Samples were sectioned (10 µm) and mounted on Visium tissue optimization slides or Visium gene expression slides. To fix and preserve the tissue morphology methanol fixation was used followed by Hematoxylin‐Eosin (HE) staining according to standard 10x Genomics protocol (CG000160, tissue fixation & H&E staining). The Keyence BZ‐X810 microscope with 2x, 10x, and 20x magnification was used light microscopy. To determine optimal permeabilization time tissue optimization was performed according to 10x Genomics protocol (CG000238, Visium spatial gene expression teagent Kits – tissue optimization user guide). For tissue permeabilization, tissue sections were incubated with permeabilization enzyme for six different times between 3 and 30 min. cDNA was visualized using fluorescence imaging (Keyence BZ‐X810) with TRITC filter cube (excitation 542/20, emission 620/52) at 2x magnification. For kidney samples, 12 min was the optimal permeabilization time point. To measure total mRNA in tissue sections and map gene activity, gene expression was performed according to 10x Genomics protocol (CG000239, Visium spatial gene expression reagent kits – user guide). Tissue sections were incubated with permeabilization enzyme for 12 min. mRNA was released and captured by poly(dT) primers on slides. cDNA was generated by reverse transcription reaction. Full‐length, barcoded double‐strand cDNA is amplified via PCR and the number of cycles was determined by qPCR using KAPA SYBR FAST qPCR Kit. For cDNA quality control and quantification Agilent high sensitivity DNA kit and Agilent 2100 Bioanalyzer was used according to specifications of the manufacturer (Agilent high sensitivity DNA kit guide). Twenty five percentage of total cDNA was used for spatial gene Expression Library Construction according to 10x Genomics protocol (CG000239, Visium spatial gene expression reagent kits – user guide). For post library construction quantification KAPA library quantification kit for Illumina platforms was used to according to specifications of the manufacturer. The spatial gene expression library construction samples were sequenced by Illumina using PE150‐NovaSeq (Novogene, UK). For this, 30 µL library construct were used. The Space Ranger (10x Genomics) workflow mkfastq was used for demultiplexing the Illumina sequencer's base call files (BCLs) for each flow cell directory into FASTQ files.

### Transcriptome Data Pre‐Processing

The BCL files generated by the sequencer were demultiplexed and converted to fastq files according to their 8‐bp multiplexing index sequences using the bcl2fastq algorithm (v.2.20; Illumina). Fastq files were preprocessed using the CeleScope tools (v1.6.1; www.github.com/singleron‐RD/CeleScope; Singleron Biotechnologies GmbH), using the default parameters except that the poly‐A filter was switched off. Briefly, the R2 reads were demultiplexing using the barcode information from read1 fastq data. Removal of low quality and adapter sequences was performed with cutadapt (https://cutadapt.readthedocs.io/en/stable/installation.html). The mapping was done using STAR (https://github.com/alexdobin/STAR) against the murine reference draft (mm10) and Ensembl 92 annoations. The reads were assigned to genes using the featureCount tool (https://subread.sourceforge.net) and the cell calling was performed by fitting a negative bimodal distribution and determining the threshold between empty wells and cell‐associated wells. The gene count matrix was then generated, providing the number of unique molecular identifier (UMI) for each gene and cell.

### scRNA‐Seq Data Analysis

Gene count matrices were then used for downstream analysis employing the Seurat package.^[^ ^73^ ^]^ Cells expressing fewer than 200 or more than 5000 genes were excluded from the analysis. Furthermore, cells with over 10% mitochondrial gene expression were also removed.

The Seurat v4.2.0 package^[^ ^73^ ^]^ was used to preprocess scRNA‐Seq data from SH045, Vehicle and ctrl independently. Log‐Normalization and SCT‐scaling were performed using the NormalizeData() and ScaleData() functions. The FindVariableGenes() function was used to select the main variable genes (in our dataset 1500) for further downstream analysis like principal component analysis (PCA) and clustering. After testing several cutoffs for FindNeighbors() the 15 first dimensions were used and for FindClusters() a resolution of 0.5 was set. To remove doublets DoubletFinder was used.^[^ ^74^ ^]^ After this adaptation of the datasets the Seurat pipeline was run again independently on the single datasets and ended with the harmonization of the different samples of SH045, Vehicle and Ctrl by Harmony.^[^ ^75^ ^]^ For visualization via UMAP and extraction of marker genes the Seurat implemented functions were used.

### Cell Type Assignment

Cell type identification was performed using ScType^[^ ^21^ ^]^ on the defined clusters from Seurat. In addition to the marker genes for cell types based on ScType the average expression values of canonical markers (Figure 1C) were analyzed. The visualized marker were selected based on literature and CellMarker 2.0 database^[^ ^19^, ^20^, ^30^ ^]^ for endothelial cells (*Pecam1* and *Meis2)*, fibroblasts (*Col1a1* and *Fn1)*, inflammatory cells (*Cd68* and *C1qa)*, distal tubule cells (*Slc12a3*), principal cells (*Aqp2* and *Hsd11b2*), proximal tubule cells (*Slc27a2* and *Lrp2*), intercalated cells (*Atp6v0d2* and *Atp6v1g3*), and Loop of Henle cells (*Slc12a1* and *Umod*). Seven immune cell types were identified by manual annotation including Macrophages, Dendritic cells, T cells, Myeloid cells, B cells, Neutrophils, and Unknown inflammatory cells. Cell markes were obtained from CellMarker 2.0 database.^[^ ^20^ ^]^ They were manually combined them into inflammatory cells cluster.

Many sub‐types of endothelial cells and fibroblasts exist, which are not specifically represented in ScType.^[^ ^21^, ^76^ ^]^ Therefore, endothelial cells were subsetted from the Seurat object and performed Log‐Normalization and SCT‐scaling using raw counts (as described above in scRNA‐Seq data analysis part). The PCA was performed with the 15 first dimensions and the resolution was set to 0.3 for FindClusters() function to allow looser neighbor interactions. Based on this approach it end up by detecting five clusters within the endothelial cells and six clusters (F0 – F5) of fibroblasts. The FindVariableGenes() function was used to select the main variable genes. To assign subpopulations of endothelial cells, first all marker genes were detected using FindAllMarkers() function with default settings. Next, top50 marker genes of 0–4 clusters were compared with the top 50 marker genes of each cluster of multi‐organ endothelial cell atlas including eight classes (large artery, capillary arterial 2, capillary arterial 1, capillary 1, capillary 2, angiogenic, vein, glomeruli).^[^ ^23^ ^]^ The number of genes matched were determined to corresponding atlas and their fraction in % using following formula (n genes matched/ 50 marker genes of the atlas) x 100%)). The majority of overlapping genes were used as assignment criteria to corresponding subpopulation of endothelial cells from the atlas. In total, four distinct subpopulations of endothelial cells were identified, namely vein endothelial cells (cluster 0), capillary arterial endothelial cells (cluster 1, 2), large artery endothelial cells (cluster 3), and novel endothelial cells (cluster 4). Since no marker genes in the cluster 4 matched any endothelial cell type, this cluster was named as novel endothelial cell. Because of the unknown biological significance, the capillary arterial 1 and capillary arterial 2, as well as capillary 1 and capillary 2 were assigned as capillary arterial and capillary, respectively.

scCODA model, rooted in the Bayesian framework was used for cellular composition analysis.^[^ ^77^ ^]^ False discovery rate (FDR) threshold was 0.3. Cell composition analysis in endothelial cell and fibroblast subpopulations was performed using miloR, a tool based on the k‐nearest neighbor (KNN) algorithm.^[^ ^78^ ^]^

### Pseudobulk RNA‐Seq and Gene Set Overrepresentation Analysis

To detect differentially expressed genes (DEGs) between SH045 and Vehicle groups, a pseudobulk approach was used.^[^ ^22^ ^]^ Normalization was performed using SCRAN (V 1.20.1)^[^ ^79^ ^]^ by deconvolving size factors for cell pools. All genes with zero expression in all cells of a cell type cluster were removed to perform dispersion and shrinkage by DESeq2 (v1.32.0).^[^ ^80^ ^]^ DESEq2 and SCRAN were used with default settings and Wald test was used to perform multiple hypothesis correction. Genes with adj. p‐value <0.05 were defined as DEGs. DEGs with positive log2 fold change were considered as upregulated for SH045 and with negative log2 fold change as downregulated.

To identify enriched pathways with up‐ or downregulated DEGs for the single cell types an gene set overrepresentation analysis was performed using DAVID.^[^ ^81^ ^]^ The DAVID settings were set to default (similarity term overlap: 3, similarity threshold: 0.5, initial group membership: 3, multiple linkage threshold: 0.5, EASE: 1.0) to functionally annotate the DEGs based on the GeneOntology (GO). The annotation was restricted to the Biological Process (BP) and used the clustering to define umbrella terms of functional hierarchy levels with overlapping gene sets. The overrepresented pathways were called significantly enriched below an adj. p value < 0.05 calculated with Benjamini Hochberg (BH) False Discovery Rate (FDR). For graphical representation, top10 BP were selected based on adj. p‐values, reordered based on gene ratio and displayed using ggplot2. Similar strategy was employed to characterize novel endothelial cells and F2 fibroblasts, based on their respective marker genes.

### Pseudotime Analysis

Monocle3 was used for pseudotime analysis (https://github.com/cole‐trapnell‐lab/monocle3). The expression matrix was imported into Monocle to create a CellDataSet (CDS) object. Then PCA linear dimensionality reduction and UMAP nonlinear dimensionality reduction was performed sequentially and integrated them into the UMAP coordinates in Seurat. Next, cell grouping was performed by the cluster_cells() function, with the resolution set to 1e‐14 so that all cells were considered as a whole cluster. Cell trajectories were constructed using the learn_graph() function.

### Transcriptional Regulatory Network Analysis

A SCENIC pipeline (v1.3.1) with default parameters was applied to identify the key transcriptomic regulators in the fibroblasts under TRPC6 blockade.^[^ ^82^ ^]^ Normalized expression data was used as input to construct a co‐expression regulatory network. Clustering of fibroblasts was based on the binary activity matrix of transcription factor regulons. The network of transcription factor and its target genes was visualized with Cytoscape software (v3.9.1).^[^ ^83^ ^]^ For the spatial sample, the transcriptional regulatory network information obtained from SCENIC calculations was imported into decoupler package to infer transcription factor activity via the Univariate Linear Model (ULM) model.^[^ ^84^ ^]^

### Gene Set Score Analysis

The AddModuleScore() function from Seurat was used to calculate the wound healing score among all fibroblast subpopulations.^[^ ^73^ ^]^ The wound healing, FAO and glycolysis gene set were obtained from the Molecular Signatures Database (MSigDB).^[^ ^85^ ^]^ The negative regulation of cell proliferation (NRCP) gene set was obtained from the overrepresentation analysis results of the target genes of *Prnp* transcription factor. The novel endothelial cell signature (novel score) gene set was obtained from the top 50 marker genes of novel endothelial cells.

### Human scRNA‐seq Data Processing

CKD patients scRNA‐seq data were downloaded from https://zenodo.org/records/4059315. The endothelial cells in the CD10^–^ samples in the data were extracted and analyzed. Dimensionality reduction and clustering of data adopted Seurat standard process as mentioned above (see scRNA‐Seq data analysis section), with a resolution of 0.5. Cell annotation followed the original author's strategy.^[^ ^24^ ^]^ Novel score was calculated using the AddModuleScore() function as described above (see Gene set score analysis section). For identifying correlated cell types between mouse and human datasets, firstthe cell type specific UMI counts were aggregated, normalized by the total count, multiplied by 100000, and log transformed after adding a pseudo‐count. Then non‐negative least squares (NNLS) regression was applied to predict the gene expression of target cell type (*T_a_*) in dataset A with the gene expression of all cell types (*M_b_*) in dataset B: *T_a_* = *β*_0_ *_a_* + *β*_1_ *_a_ M_b_*, where *T_a_* and *M_b_* represent filtered gene expression for target cell type from data set A and all cell types from data set B, respectively.^[^ ^86^ ^]^

### Spatial Transcriptome Data Processing

Filtered feature‐barcode expression matrices obtained from SpaceRanger (v2.0.1) served as the initial input for spatial transcriptomics analysis using Seurat (v4.2.0)^[^ ^73^ ^]^ and Scanpy (v1.9.3).^[^ ^87^ ^]^ Mitochondrial genes were excluded from the analysis. The raw count matrix for two samples (one SH045 and one Vehicle group) underwent multi‐sample integration, batch effect removal, and spatial clustering facilitated by the PRECAST package.^[^ ^88^ ^]^ Exploring various values of K to align with the optimal histological structural division, K = 10 was ultimately selected. Spatially variable genes were calculated with “SPARKX” method. Default options were utilized for other parameters in the analysis.

Cell‐type compositions for each spatial spot were computed using cell2location.^[^ ^89^ ^]^ Reference expression signatures of major cell types and subtypes were estimated through regularized negative binomial regressions employing our scRNA‐seq data for each group. Subsequently, each slide underwent deconvolution using hierarchical Bayesian models, implemented in run_cell2location(). Key hyperparameters included 10 cells per spot and a detection alpha of 200.

### Intercellular Communication Analysis

CellChat was employed to analyze intercellular communication as described.^[^ ^26^, ^27^ ^]^ The ligand receptor database CellChatDB.mouse was imported, and the probability of intercellular communication and the predicted communication network were subsequently calculated. Briefly, the signaling roles (senders or receivers) of cell populations were identified by computing network centrality metrics for each population. The F2 fibroblasts was removed from differential analysis since it appeared only in SH045 group. To access proximal tubule cell, distal tubule cell, intercalated cells, loop of Henle cells and principal cells were combined into kidney cells.

To evaluate alterations in intercellular interactions among distinct cell subpopulations in spatial transcriptomic experiments, a pseudo‐single‐cell file incorporating spatial information was generated for each spatial sample. This file was based on the deconvolution results obtained from cell2location. Subsequently, an analysis of intercellular communication was conducted using node‐centric expression model (NCEM).^[^ ^28^ ^]^ The NCEM model was configured with “radius = None”, as the deconvoluted Visium data would aggregate interactions within individual spots.

For each spatial spot, signaling pathway activities were determined utilizing PROGENy's model matrix using the top 500 genes from each transcriptional footprint.^[^ ^84^, ^90^ ^]^

## Conflict of Interest

The authors declare no conflict of interest.

## Author Contributions

Y.X., and Z.Z. contributed equally to this work. M.G. and D.T. performed conceptualization. Y.X. performed data curation. Y.X. and Z.Z. performed formal analysis. M.G. and D.T. performed funding acquisition. Y.X., Z.Z., M.O., G.C., and Q.Z. performed investigation. M.G. and D.T. performed project administration. J.L., U.K., M.S., H.G., N.E., M.G., S.S., and D.T. provided resources. Y.X. was responsible for software implementation. M.G. and D.T. performed supervision. D.T., Y.X., Z.Z performed validation. Y.X. drafted the original manuscript. M.G., S.S., and D.T. reviewed, and edited the draft. All authors agree to be accountable for all aspects of the work in ensuring that questions related to the accuracy or integrity of any part of the work are appropriately investigated and resolved. All authors made substantial contributions to conception, design, drafting and completion of the article. All authors have read and agreed to the published version of the manuscript.

## Supporting information



Supporting Information

Supporting Information

Supplemental Table 1

Supplemental Table 2

Supplemental Table 3

Supplemental Table 4

## Data Availability

The data that support the findings of this study are openly available in Gene Expression Omnibus (GEO) at https://www.ncbi.nlm.nih.gov/geo/, reference number 269062.
