# Supplementary material for: Single‐Cell RNA Sequencing Delineates Renal Anti‐Fibrotic Mechanisms Mediated by TRPC6 Inhibition
Source: Adv Sci (Weinh). 2025 Jun 17;12(33):e01175. doi: 10.1002/advs.202501175 (PMC12412467; doi:10.1002/advs.202501175)
Supplement: Supplementary file 1 — Supporting Information [file ADVS-12-e01175-s006.pdf]

ADVANCED SCIENCE
Open Access

Supporting Information

for *Adv. Sci.*, DOI 10.1002/advs.202501175

Single-Cell RNA Sequencing Delineates Renal Anti-Fibrotic Mechanisms Mediated by TRPC6 Inhibition

*Yao Xu, Zhihuang Zheng, Marleen Silke Oswald, Guozhe Cheng, Jun Liu, Qidi Zhai, Ute Kruegel, Michael Schaefer, Holger Gerhardt, Nicole Endlich, Maik Gollasch, Stefan Simm and Dmitry Tsvetkov**

SH045 impact on kidney after UUO

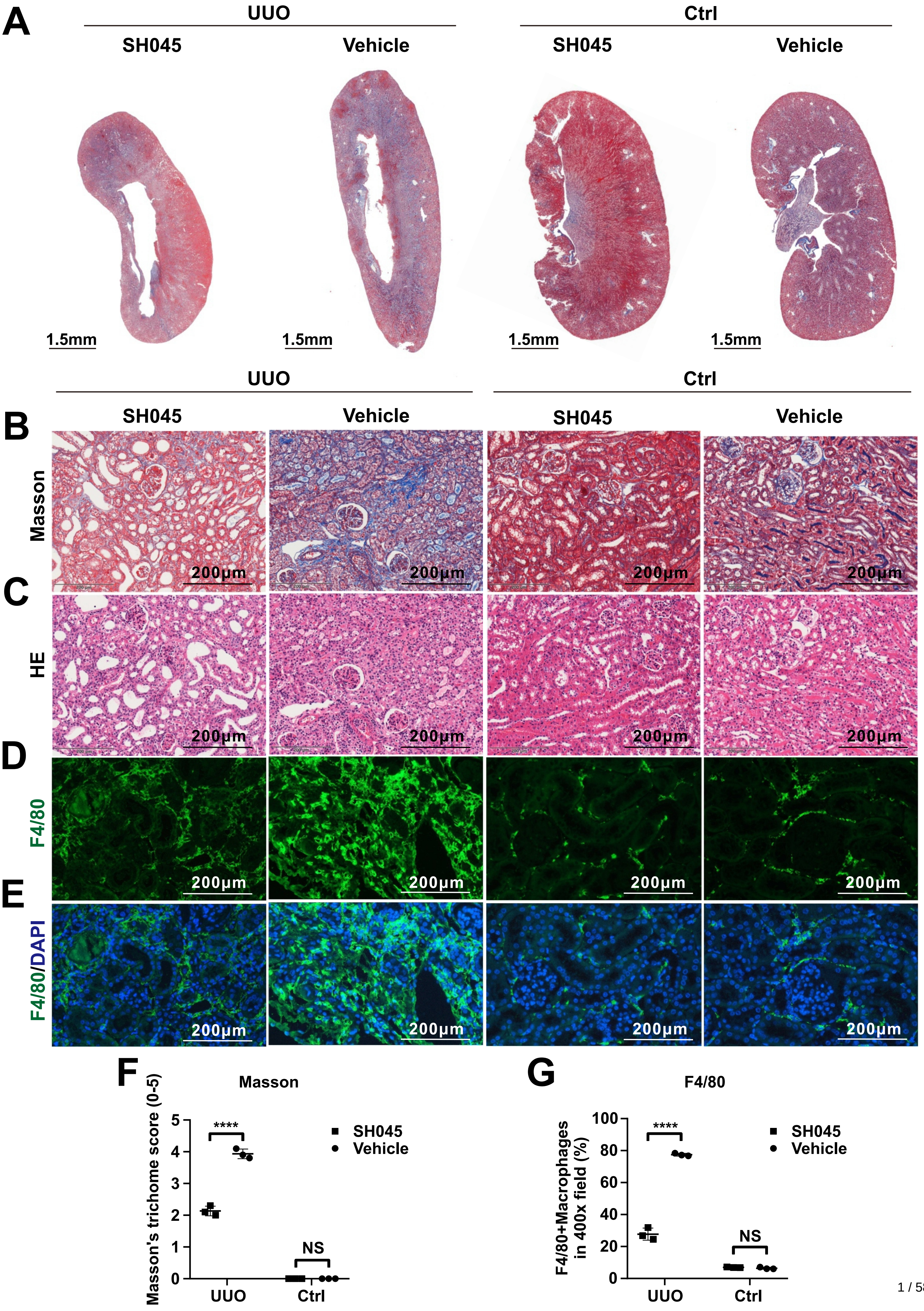

Supplementary Figure 1. SH045 impact on kidney after UUO.

(A) Representative images of longitudinal kidney sections UUO (left panel) and Ctrl (right panel) groups (scale bar: 1.5mm). Representative images of UUO and Ctrl kidneys stained with Masson' trichrome **(B)**, hematoxylin-eosin (HE) **(C)** and F4/80+ cells **(D)** **(E)** (magnification: 400x, scale bar: 200μm). Quantification of Masson's trichome score **(F)** and renal F4/80+cell infiltration **(G)**. (UUO n=3, Ctrl n=3). Data expressed as mean ± SEM. Two-way ANOVA followed by Sidak's multiple comparisons post hoc test was used. * $p < 0.05$, **** $p < 0.0001$, NS, not statistically significant.

Expression of α-SMA and TRPC6 in patients and mice

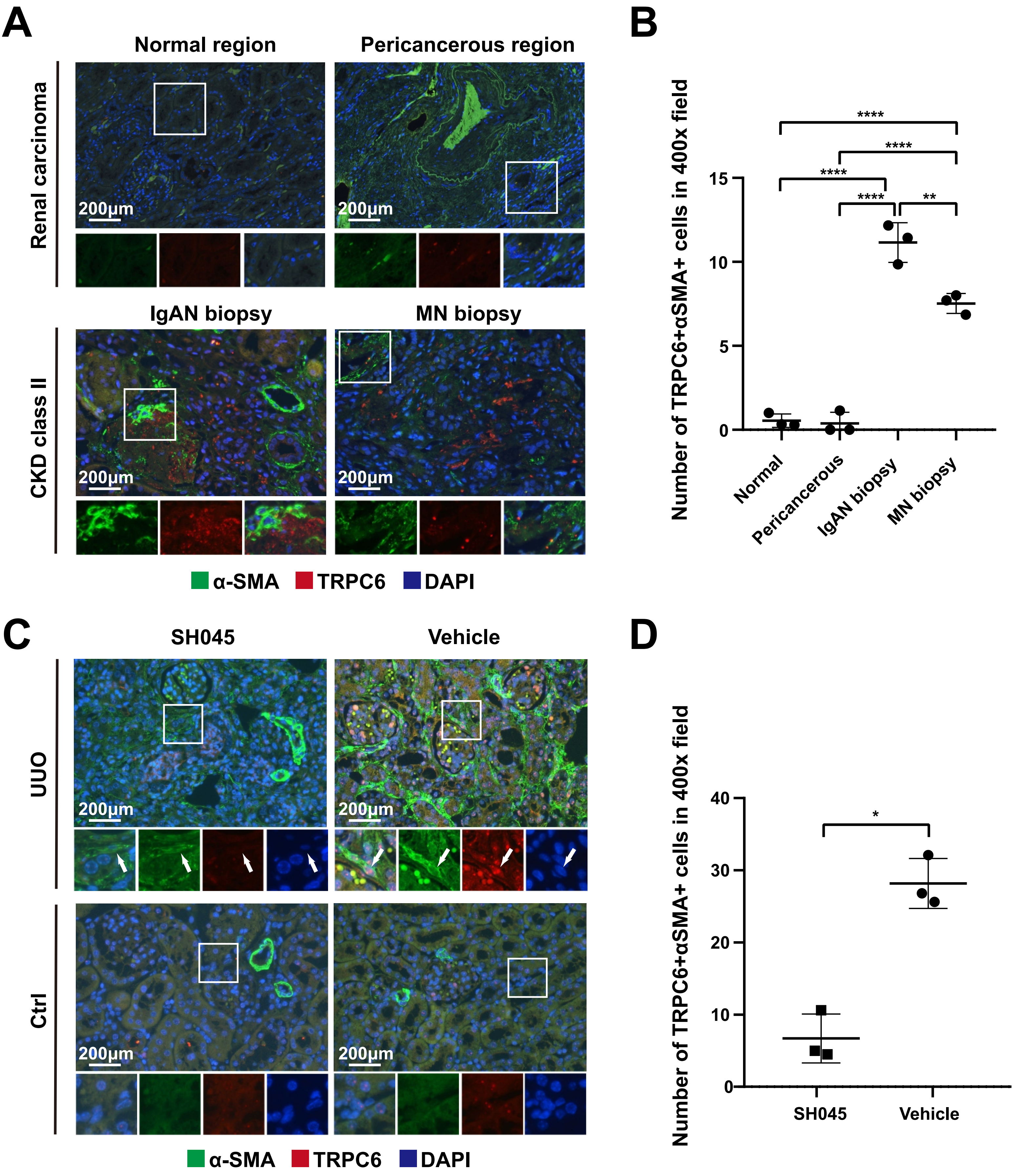

Supplementary Figure 2. Expression of α-SMA and TRPC6 in patients and mice.

(A) Representative immunofluorescence staining images of α-SMA and TRPC6 colocalization in kidneys from renal cell carcinoma (upper panel) and CKD (lower panel) patients. (magnification: 400x, scale bar: 200μm). **(B)** Quantification of α-SMA+TRPC6+ cells in kidney sections from renal cell carcinoma and CKD patients. (Normal n=3, Pericancerous n=3, IgAN n=3, MN n=3). **(C)** Representative immunofluorescence staining images of α-SMA and TRPC6 colocalization of kidneys from UUO (upper panel) and Ctrl (lower panel) mice. (magnification: 400x, scale bar: 200μm). (B) Quantification of α-SMA+TRPC6+ cells in kidney sections from SH045 (left panel) and Vehicle (right panel) treated mice. (SH045 n=3, Vehicle n=3). Data expressed as mean ± SEM. One-way ANOVA followed by Tukey's multiple comparisons post hoc test was used in the panel B. Mann-Whitney U-test was used in panel D, ** $p < 0.01$, **** $p < 0.0001$. CKD: chronic kidney diseases, IgAN: IgA nephropathy, MN: membranous nephropathy.

Expression of α-SMA and TRPC6 in ischemia-reperfusion 2m post I/R mice

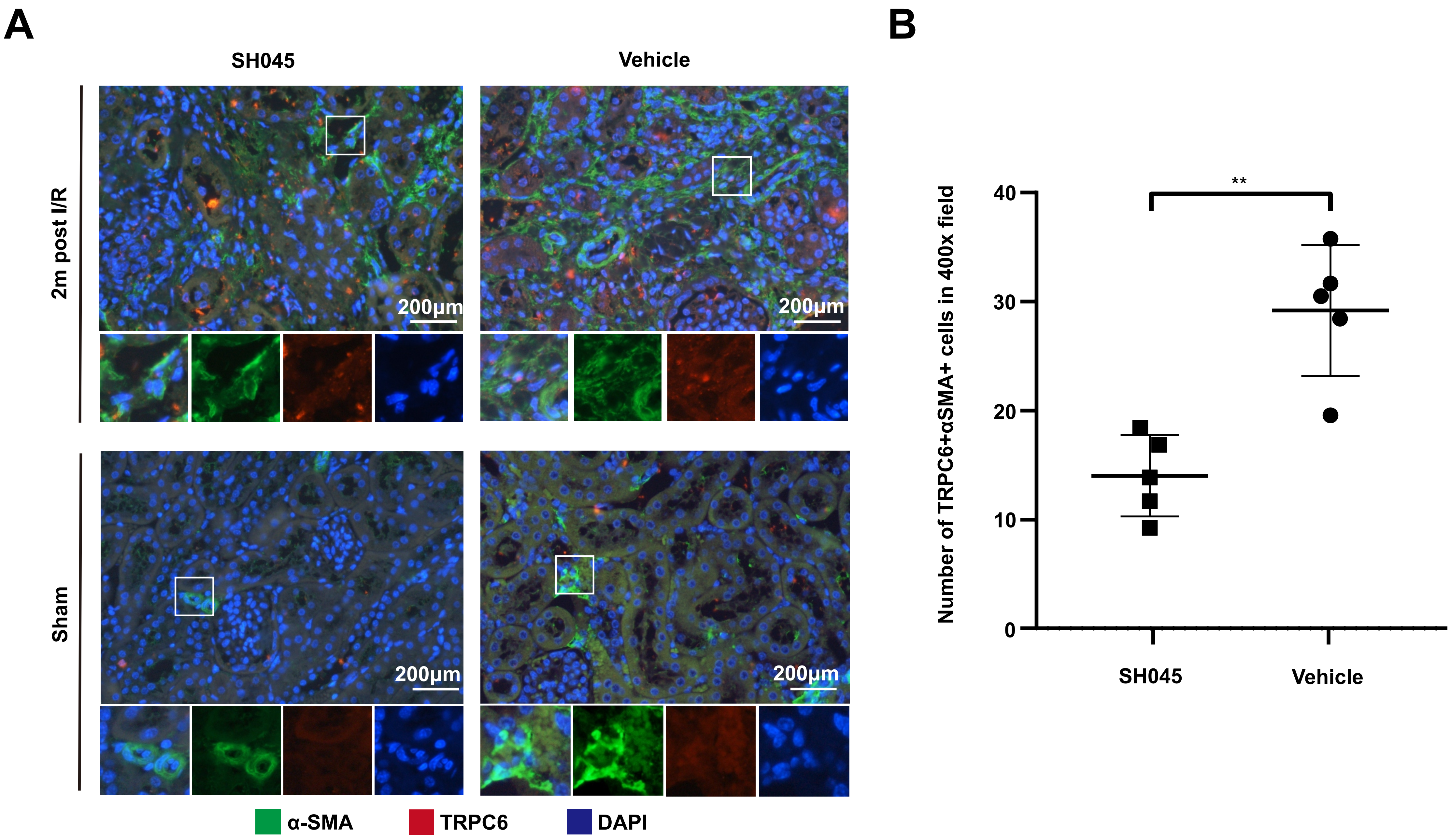

Supplementary Figure 3. Expression of α-SMA and TRPC6 in 2m post I/R model.
(A) Representative immunofluorescence staining images of α-SMA and TRPC6 colocalization of kidneys from 2m post I/R (upper panel) and Sham (lower panel) mice. (magnification: 400x, scale bar: 200μm). (B) Quantification of α-SMA+TRPC6+ cells in kidney sections from SH045 (left panel) and Vehicle (right panel) treated mice. (SH045 n=5, Vehicle n=5). Data expressed as mean ± SEM. Mann-Whitney U-test was used in panel B, ** $p < 0.01$.

Quality control summary of scRNA-seq from mouse UUO kidney samples

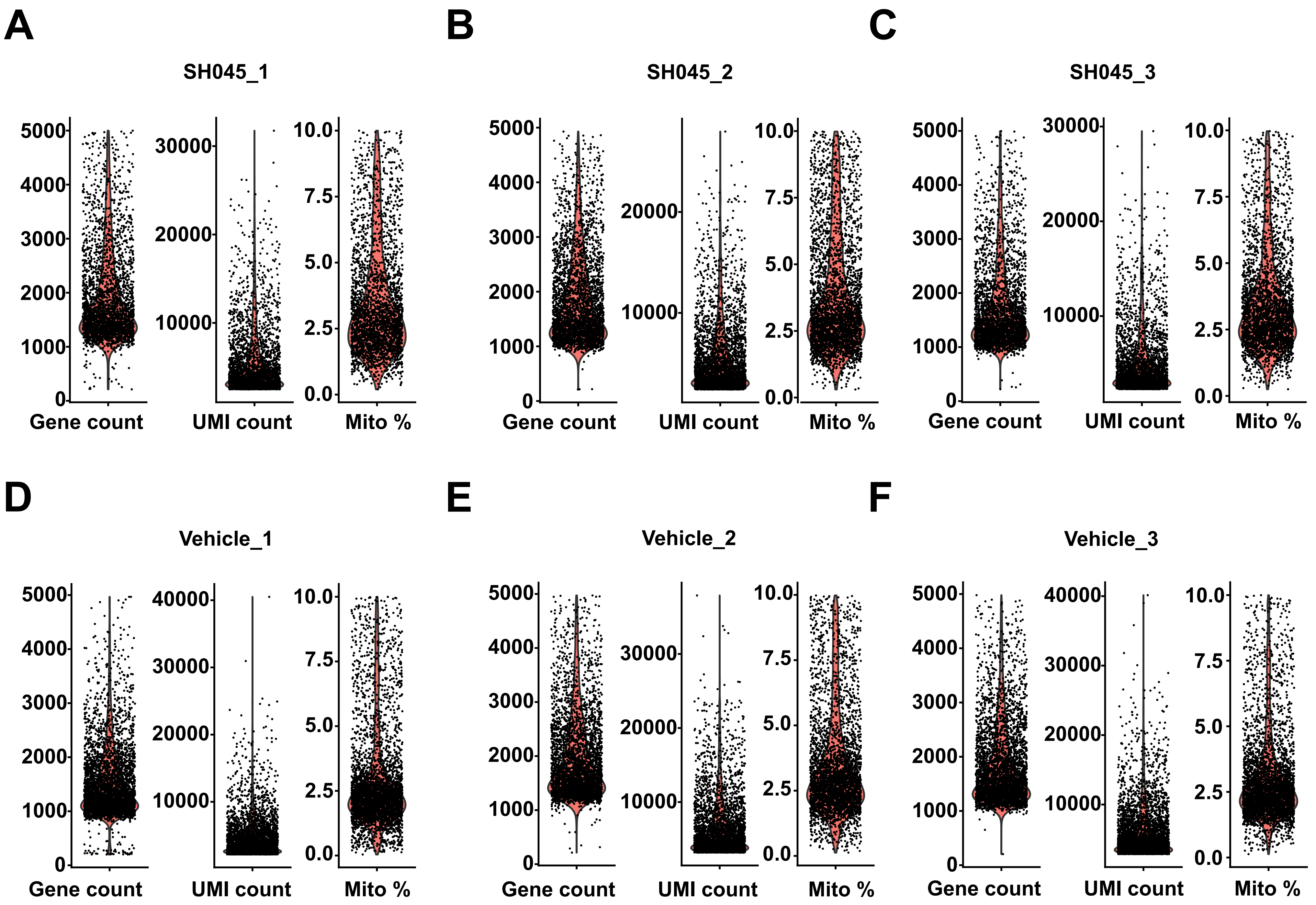

Supplementary Figure 4. Quality control summary of scRNA-seq from mouse UUO kidney samples.

Scatter plots show the distribution of the number of detected genes per cell (left), the total number of RNA molecules per cell (middle), and the proportion of mitochondrial gene expression (right) in SH045 **(A) (B) (C)** and Vehicle **(D) (E) (F)** group samples. Cells with fewer than 200 genes or with mitochondrial gene expression greater than 10% were filtered out.

Expression levels of representative marker genes across major cell types

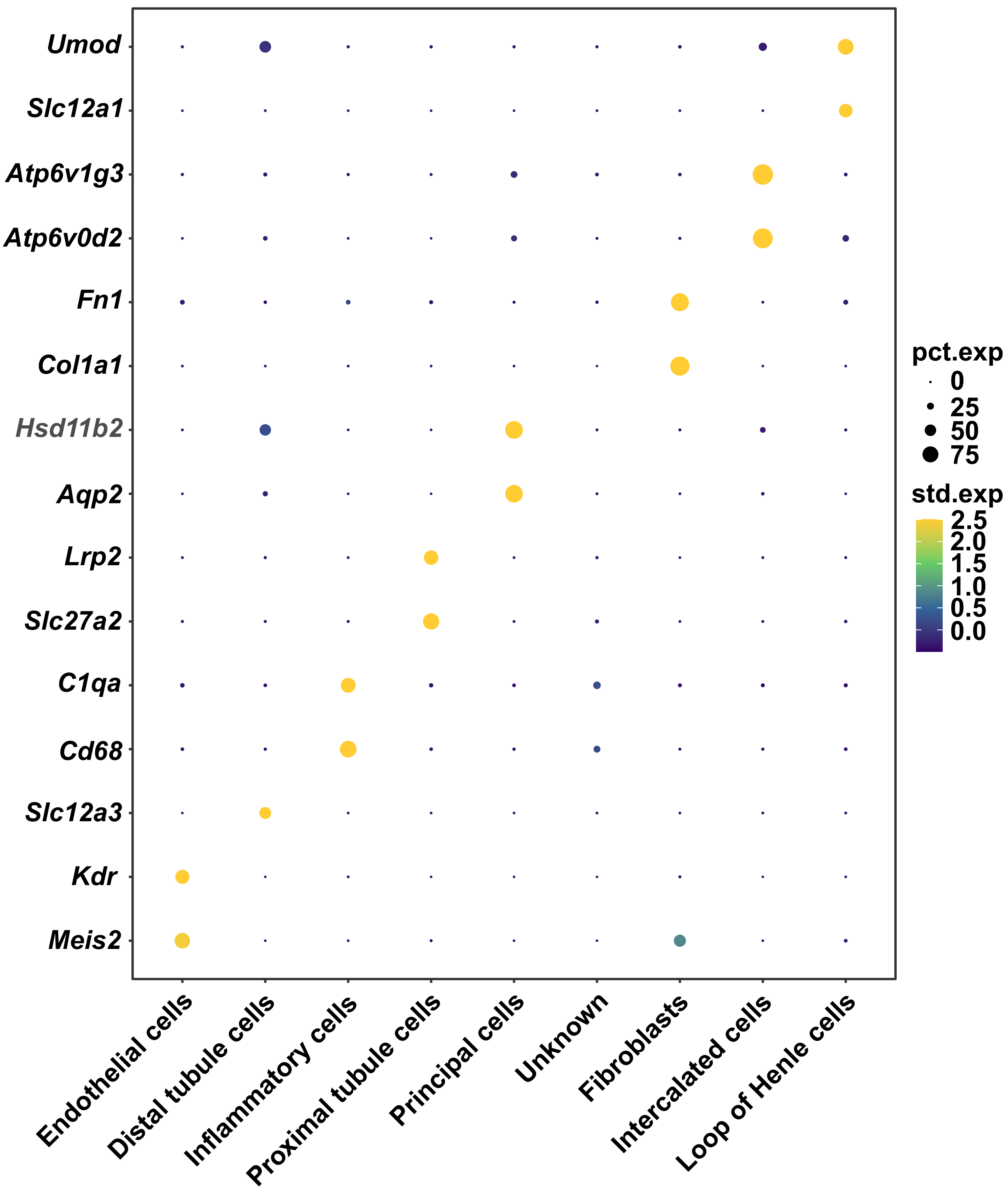

Supplementary Figure 5. Dot plot showing the expression levels of representative marker genes across major cell types in SH045 and Vehicle group samples. The size of the dots indicates the percentage of positive cells, and the color indicates the average expression. pct.exp, percentage expression; std.exp, standardized expression.

Number of assigned cell types in kidneys isolated from SH045 and Vehicle treated mice

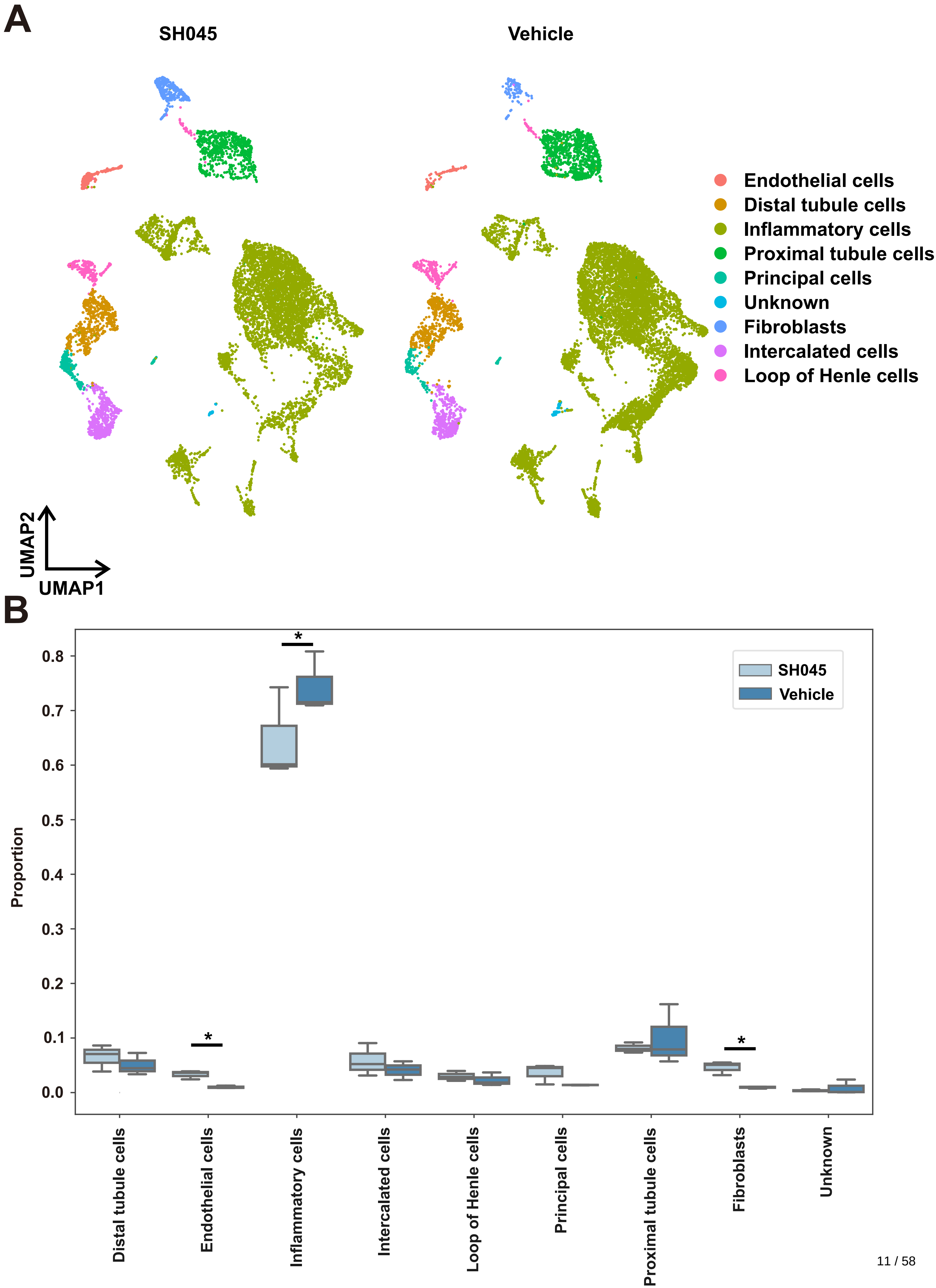

Supplementary Figure 6. Number of assigned cell types in kidneys isolated from SH045 and Vehicle treated mice.
(A) Uniform manifold approximation and projection (UMAP) and **(B)** Boxplots of compositional changes from SH045 (light blue) to Vehicle (dark blue) group mice. * $p < 0.05$ using a Logit-normal spike-and-slab prior with a log-link function in scCODA.

Number of inflammatory cell types in kidneys isolated from SH045 and Vehicle treated mice

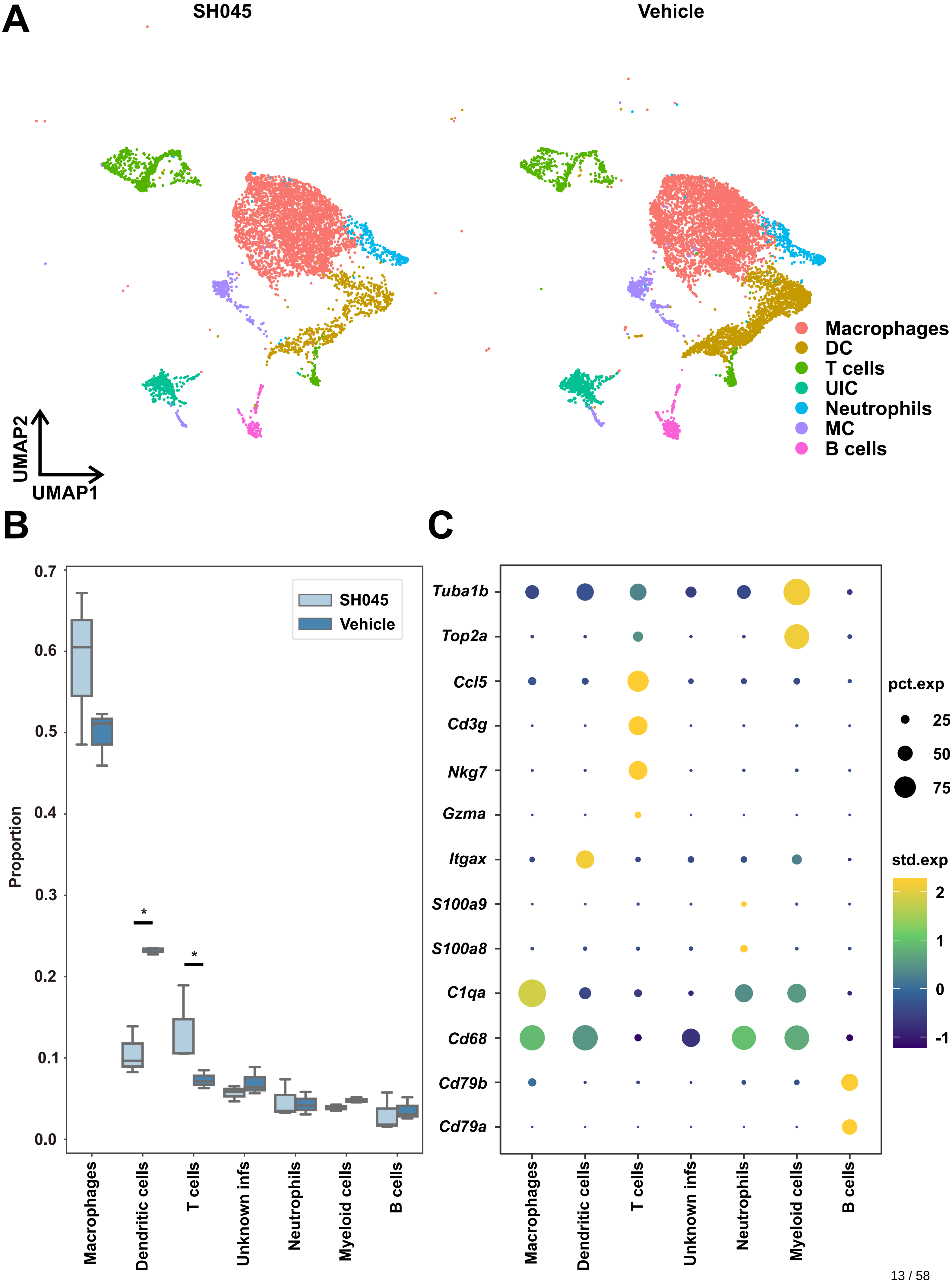

Supplementary Figure 7. Number of inflammatory cell types in kidneys isolated from SH045 and Vehicle treated mice.

(A) UMAP and **(B)** Boxplots of compositional changes in inflammatory cells from SH045 (light blue) to Vehicle (dark blue) group mice. DC, Dendritic cells; UIC, Unknown inflammatory cells; MC, Myeloid cells. * $p < 0.05$ using a Logit-normal spike-and-slab prior with a log-link function in scCODA. **(C)** Dot plot showing the expression levels of representative marker genes across inflammatory subtypes in SH045 and Vehicle group samples. The size of the dot indicates the percentage of positive cells, and the color indicates the standardized expression. pct.exp, percentage expression; std.exp, standardized expression.

Enrichment analysis of differentially expressed genes (DEGs) between SH045 and vehicle groups

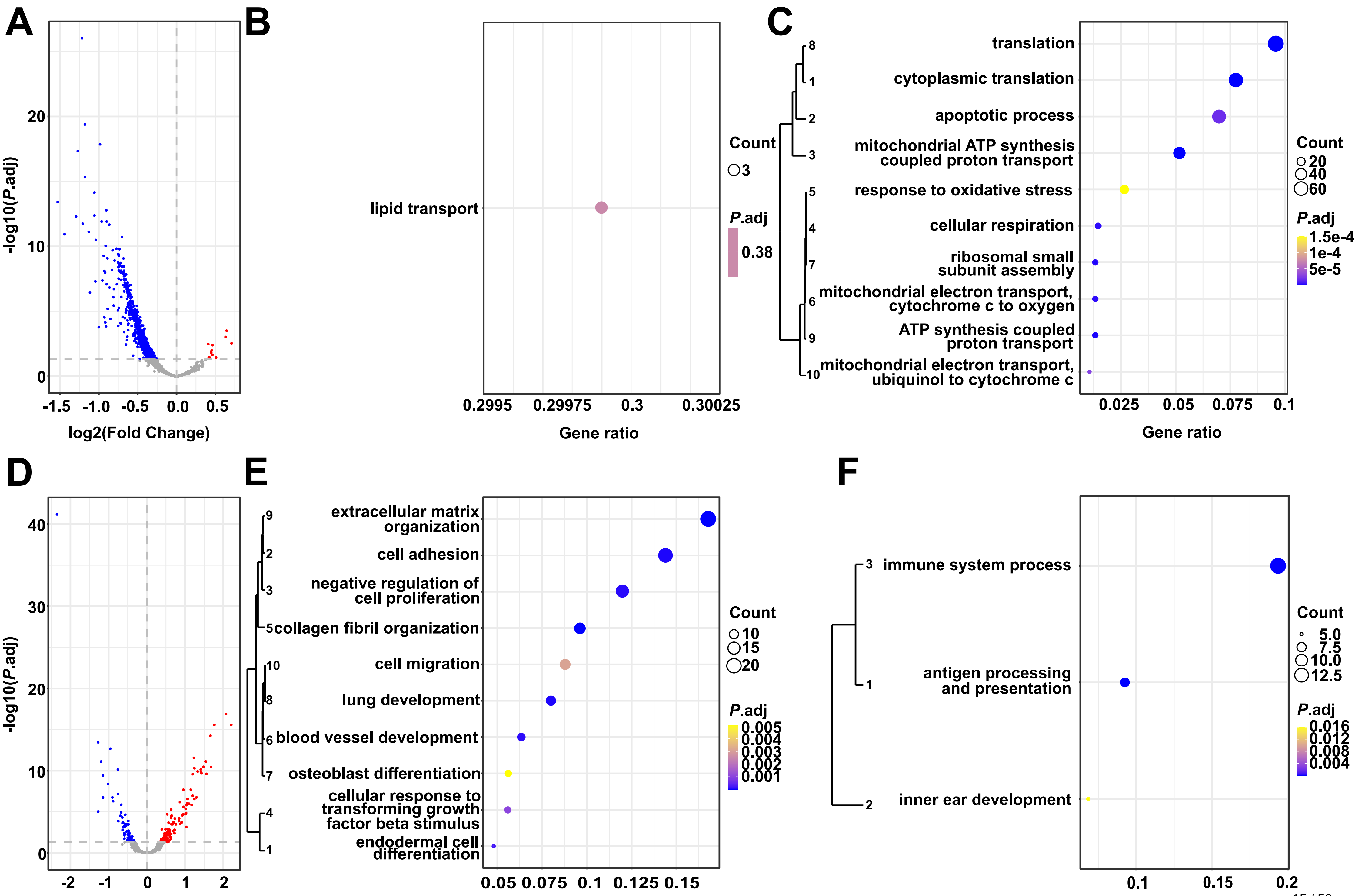

Supplementary Figure 8. Enrichment analysis of differentially expressed genes (DEGs) between SH045 and vehicle groups using pseudobulk RNA-Seq.

(**A**) DEGs between SH045 and vehicle groups in renal endothelial cells. Top upregulated **(B)** or downregulated **(C)** Gene ontology (GO) terms in renal endothelial cells. (**D**) DEGs between SH045 and vehicle groups in renal fibroblasts. Top upregulated **(E)** or downregulated **(F)** GO terms in renal fibroblasts.

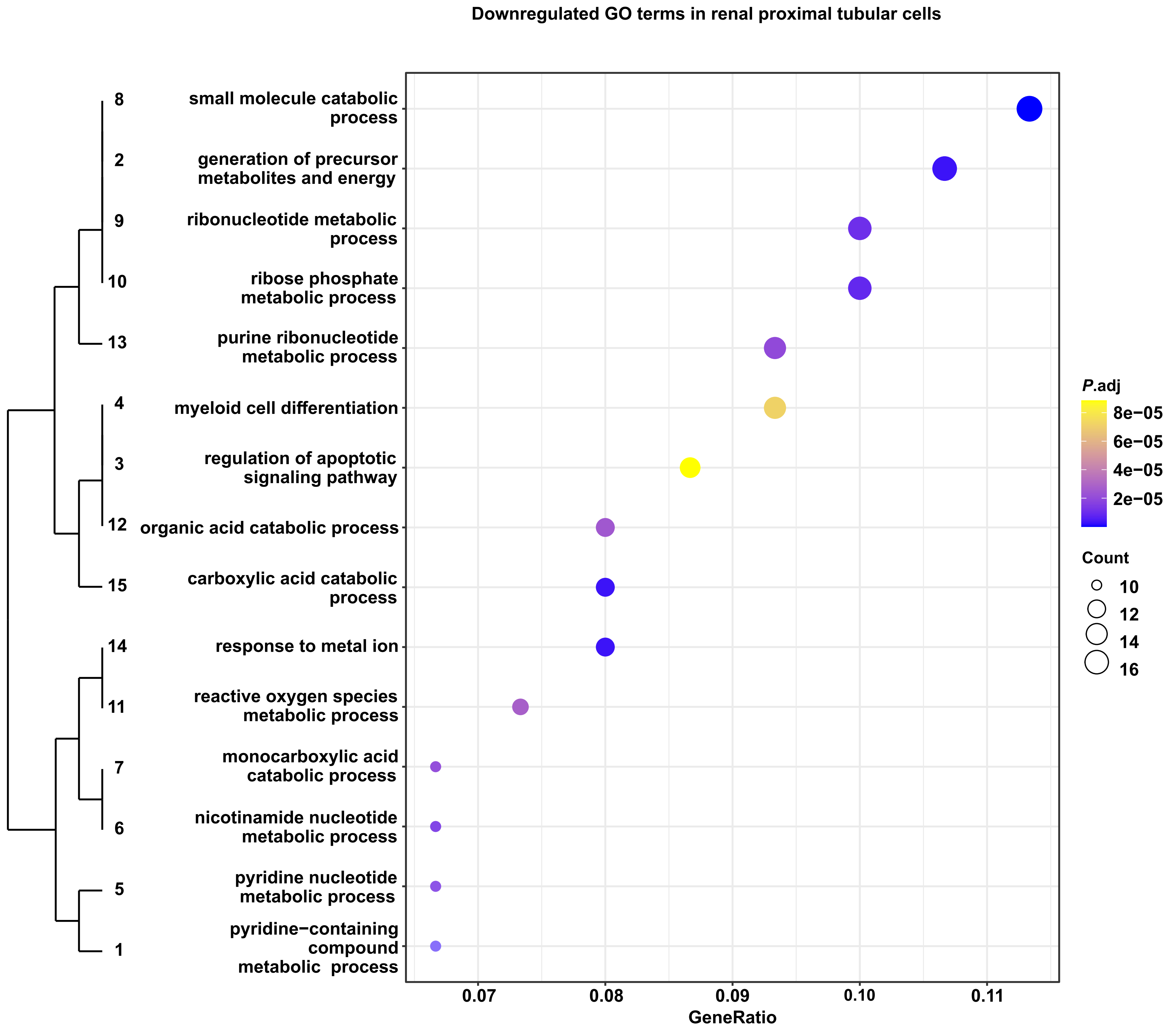
Downregulated GO terms in renal proximal tubular cells
8
2
9
10
13
4
3
12
15
14
11
7
6
5
1
small molecule catabolic process
generation of precursor metabolites and energy
ribonucleotide metabolic process
ribose phosphate metabolic process
purine ribonucleotide metabolic process
myeloid cell differentiation
regulation of apoptotic signaling pathway
organic acid catabolic process
carboxylic acid catabolic process
response to metal ion
reactive oxygen species metabolic process
monocarboxylic acid catabolic process
nicotinamide nucleotide metabolic process
pyridine nucleotide metabolic process
pyridine−containing compound metabolic process
0.07
0.08
0.09
0.10
0.11
GeneRatio
P.adj
8e−05
6e−05
4e−05
2e−05
Count
10
12
14
16

Supplementary Figure 9. Downregulated GO terms in renal proximal tubular cells.

The number of DEGs in each cell type

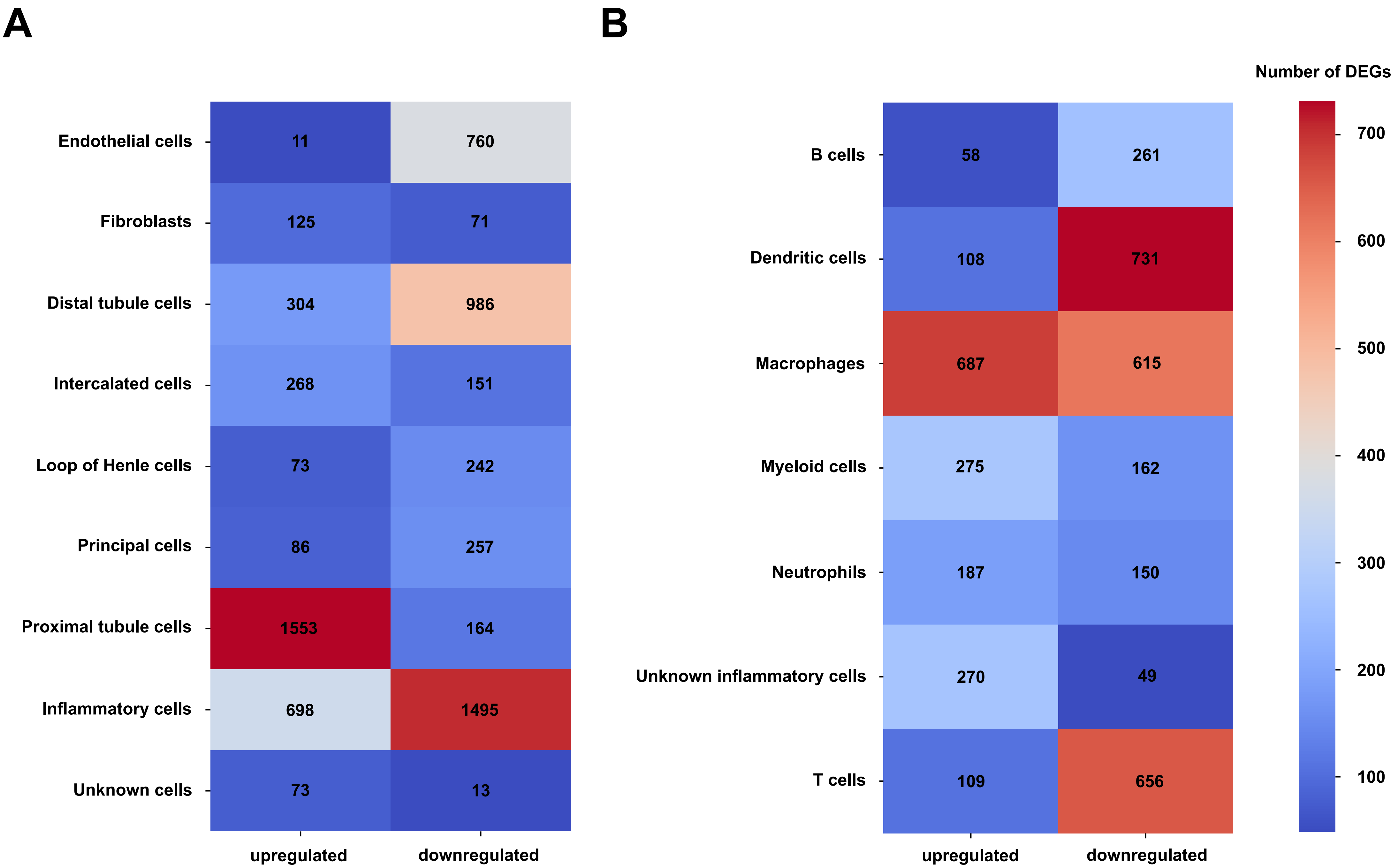

Supplementary Figure 10. Heatmap showing the number of DEGs in major cell types **(A)** and inflammatory cell subsets **(B)**.

Presence of novel endothelial cells in kidneys from patients with CKD

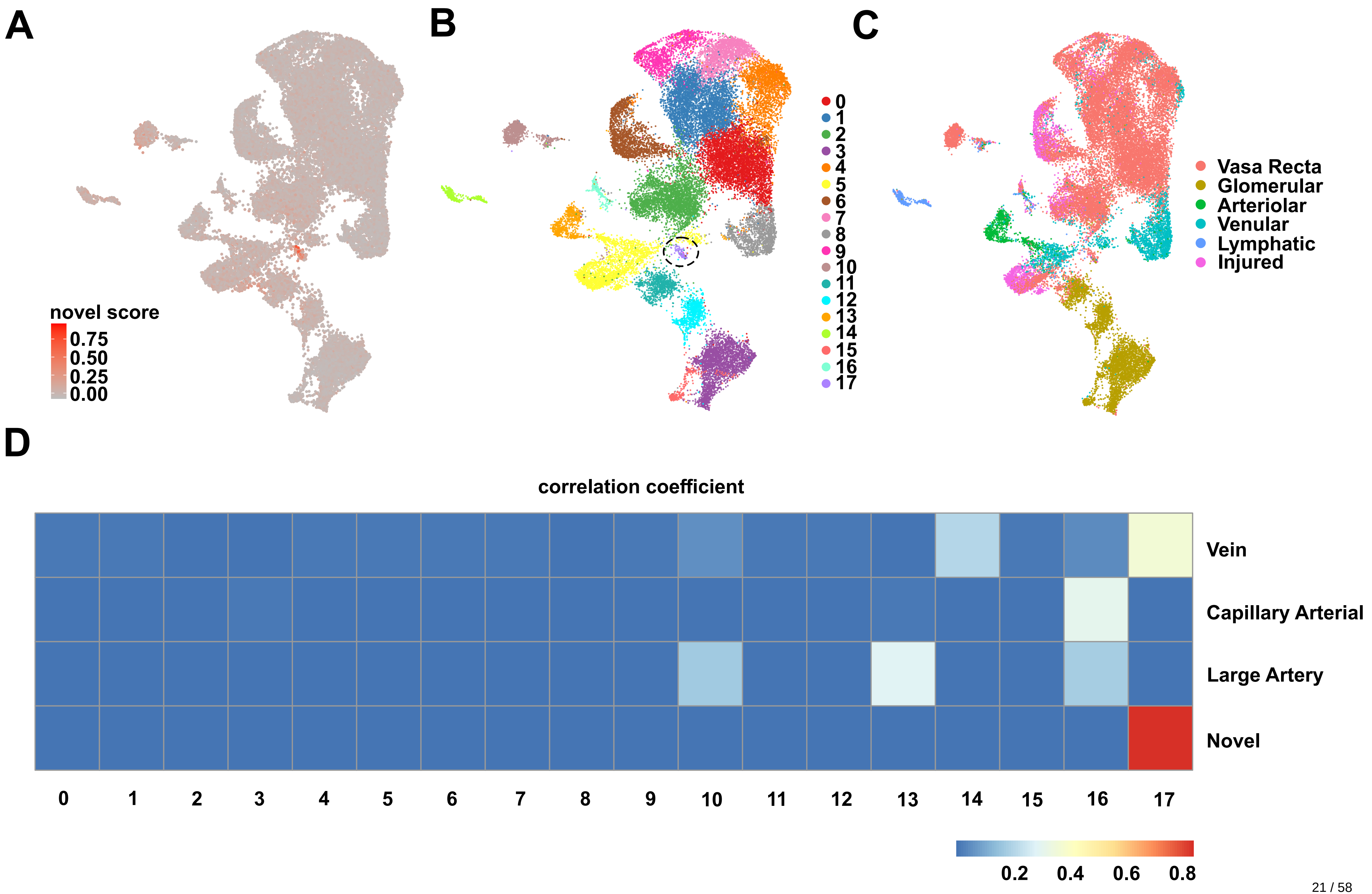

Supplementary Figure 11. Presence of novel endothelial cells in kidneys from patients (n=15) with CKD.

UMAP plot showing novel endothelial cell signature score (novel score) **(A)**, cell clustering **(B)**, and cell annotation for endothelial cells **(C)**. Non-negative least squares regression (NNLS) shows correlation coefficient between mouse and human endothelial cell subpopulations **(D)**. Dashed circle represents human endothelial cell cluster 17, which has a high novel score.

Presence of novel endothelial cells in kidneys from patients with unaffected kindey function and patients with CKD and DKD

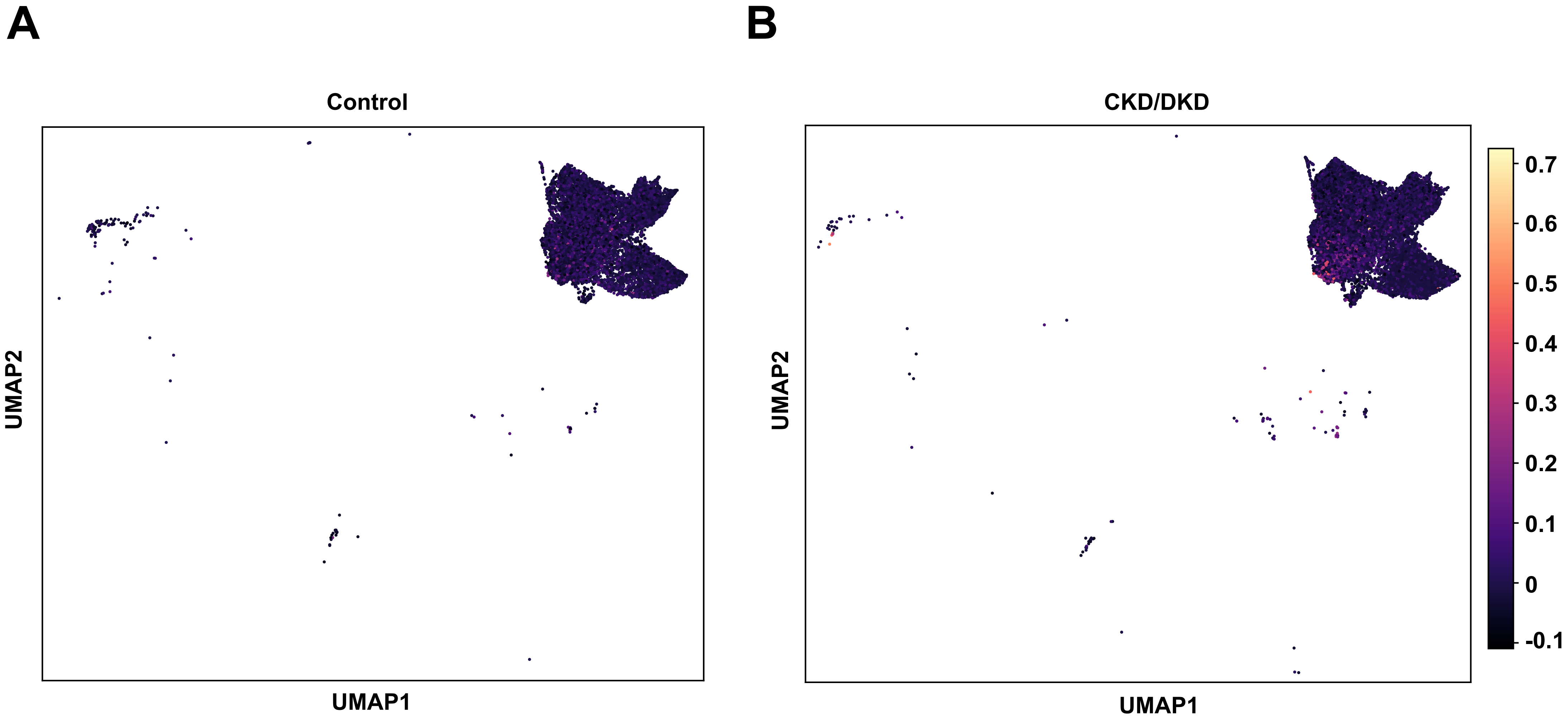

Supplementary Figure 12. Presence of novel endothelial cells in kidneys from patients with unaffected kindey function (n=26) and patients with CKD (n=20) and diabetic kidney disease (DKD) (n=12).
UMAP plot showing novel endothelial cell signature score (novel score) in Control **(A)** and CKD/DKD group **(B)**.

Expression levels of representative marker genes across fibroblast subtypes

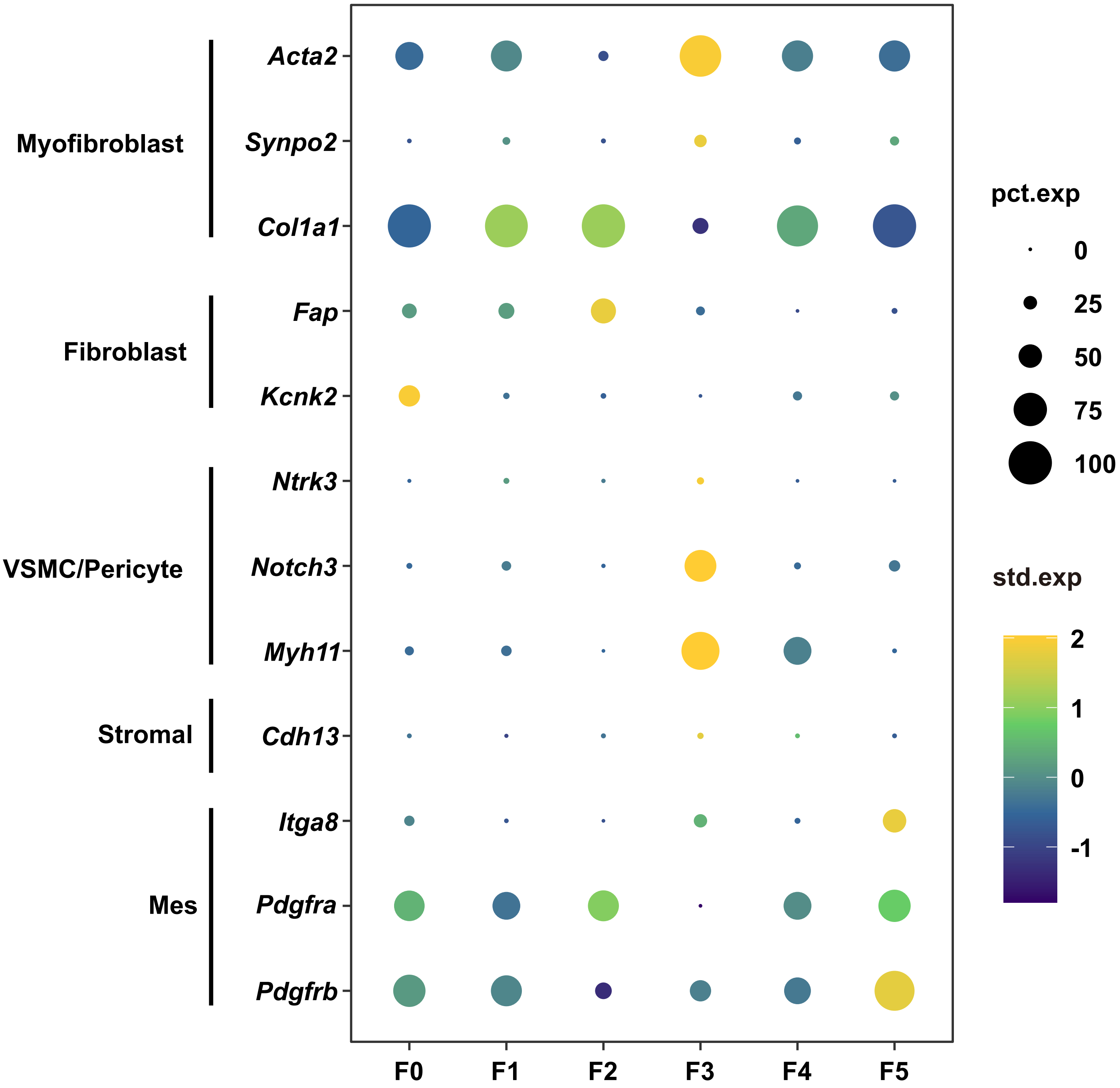

Supplementary Figure 13. Dot plot showing the expression levels of representative marker genes across fibroblast subtypes in SH045 and Vehicle group samples. The size of the dot indicates the percentage of positive cells, and the color indicates the standardized expression. VSMC: vascular smooth muscle cells, Mes: mesangial cells. pct.exp, percentage expression; std.exp, standardized expression.

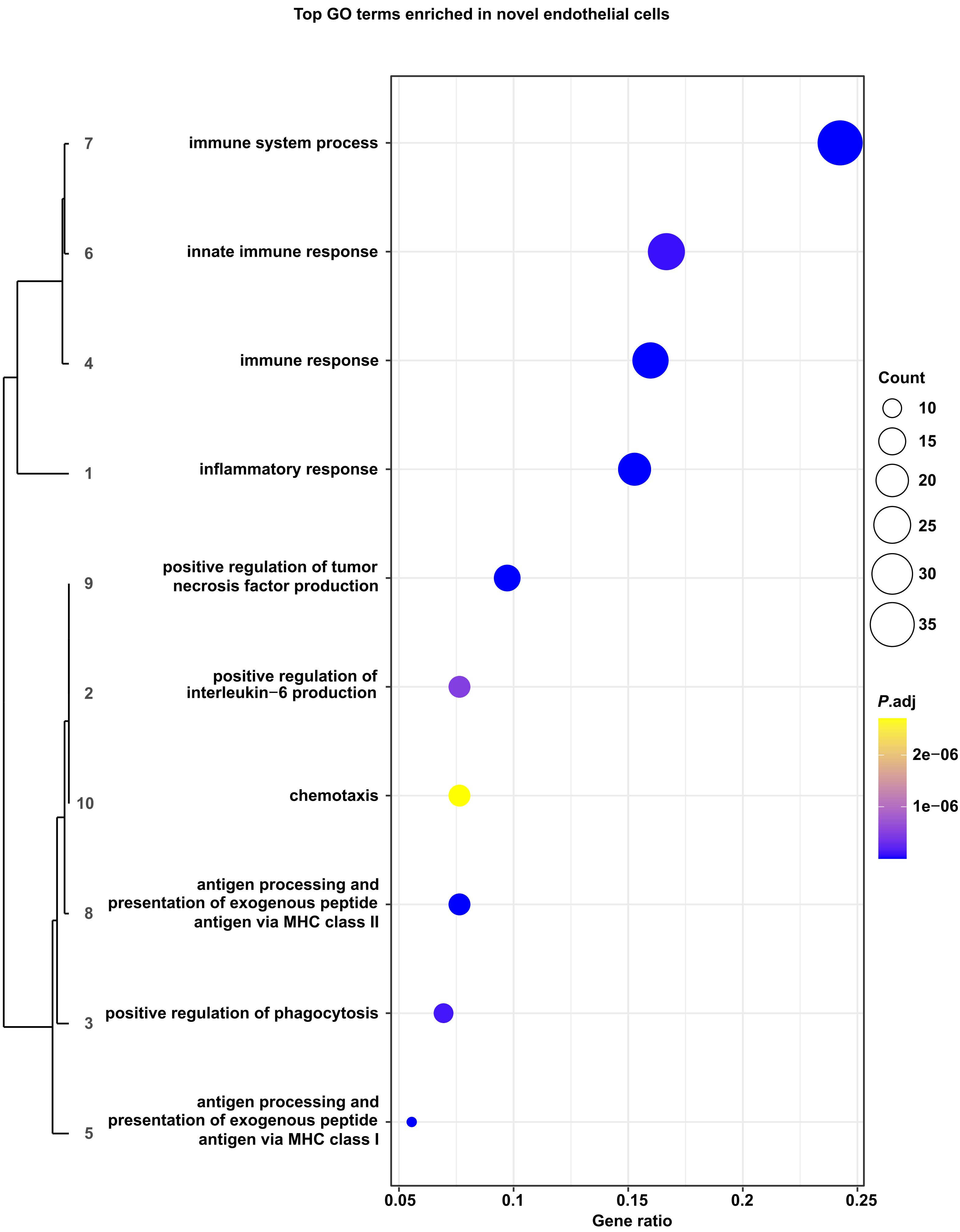
Top GO terms enriched in novel endothelial cells
7
6
4
1
9
2
10
8
3
5
immune system process
innate immune response
immune response
inflammatory response
positive regulation of tumor necrosis factor production
positive regulation of interleukin−6 production
chemotaxis
antigen processing and presentation of exogenous peptide antigen via MHC class II
positive regulation of phagocytosis
antigen processing and presentation of exogenous peptide antigen via MHC class I
0.05
0.1
0.15
0.2
0.25
Gene ratio
Count
10
15
20
25
30
35
P.adj
2e−06
1e−06

Supplementary Figure 14. Top GO terms enriched in novel endothelial cells.

Comparison of glycolysis and fatty acid oxidation (FAO) score among endothelial cell subpopulations

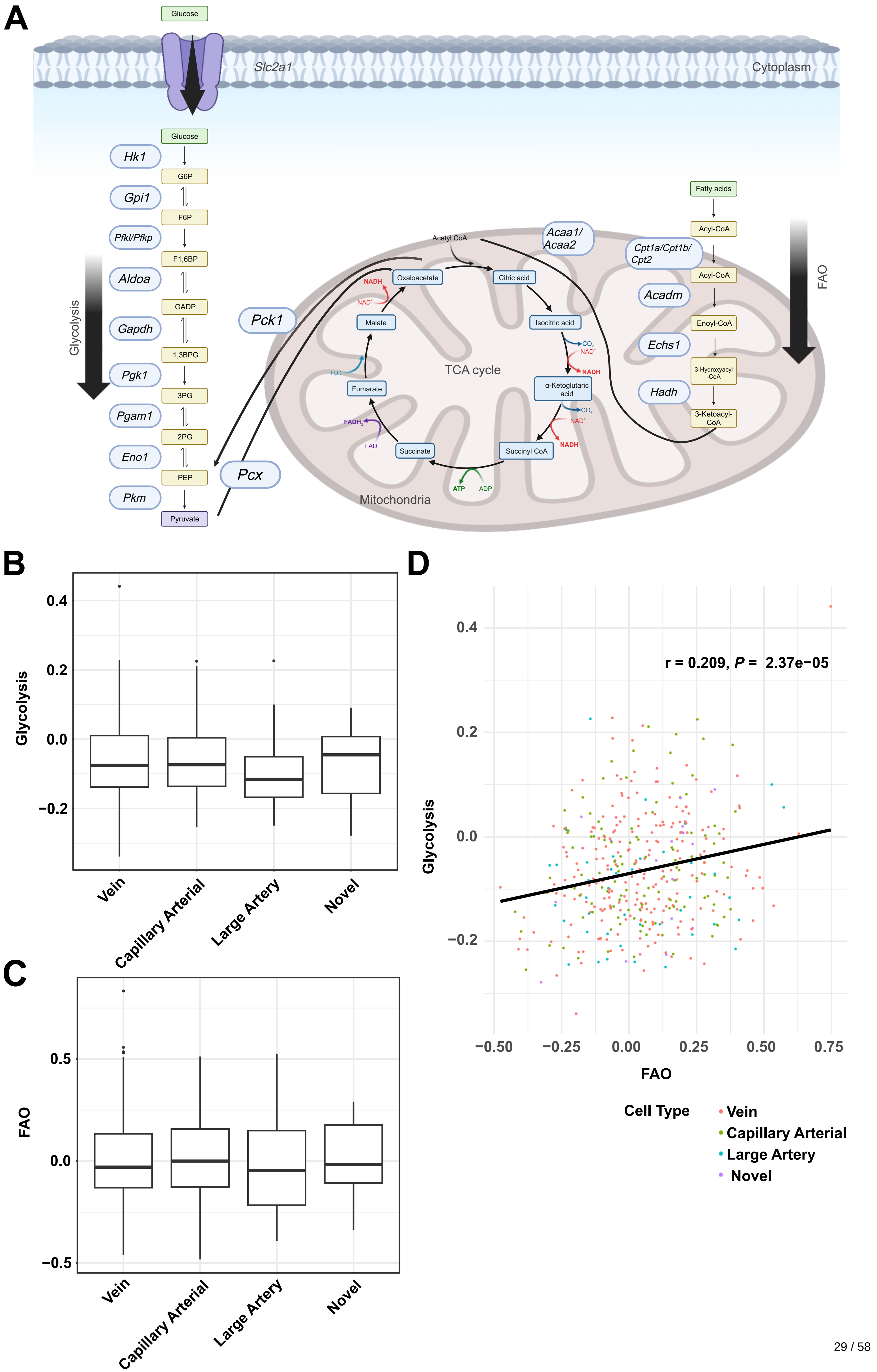

Supplementary Figure 15. Comparison of glycolysis and fatty acid oxidation (FAO) score among endothelial cell subpopulations.

(A) Schematic illustration of glycolysis and FAO pathways. Boxplots showing glycolysis **(B)** and FAO **(C)** score of endothelial cell subpopulations. **(D)** Scatter plot showing the correlation between glycolysis and FAO score.

Pathways and transcription factor activity (Prnp) in F2 fibroblasts

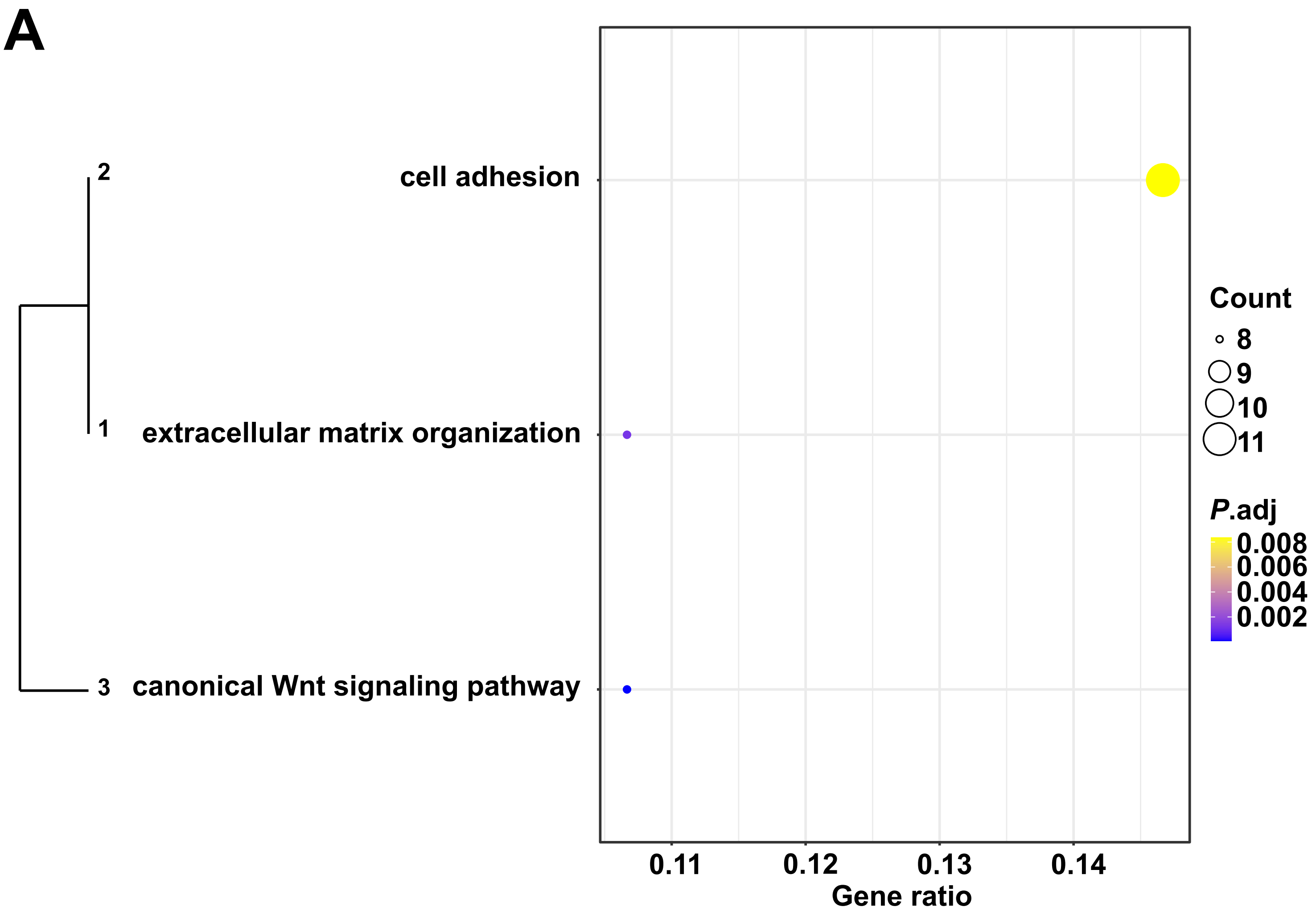

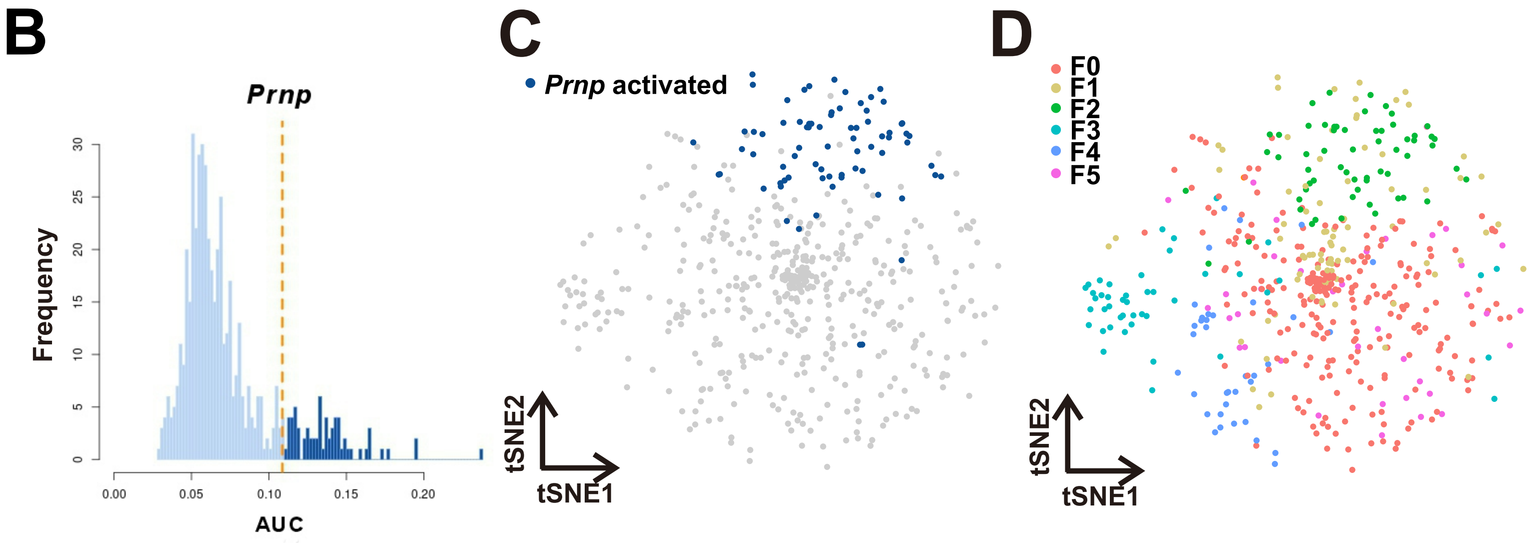

Supplementary Figure 16. Pathways and transcription factor activity (*Prnp*) in F2 fibroblasts. **(A)** Top GO terms enriched in F2 fibroblasts. **(B)** Histogram showing AUC thresholds defining *Prnp* activation. **(C)** t-distributed stochastic neighbor embedding (tSNE) plots based on the binary activity matrix after applying SCENIC: computational method for simultaneous gene regulatory network reconstruction and cell-state identification. **(D)** t-SNE plot based on the expression matrices, colored by fibroblast subpopulations.

Quality control summary of spatial transcriptomics data from mouse UUO kidney samples

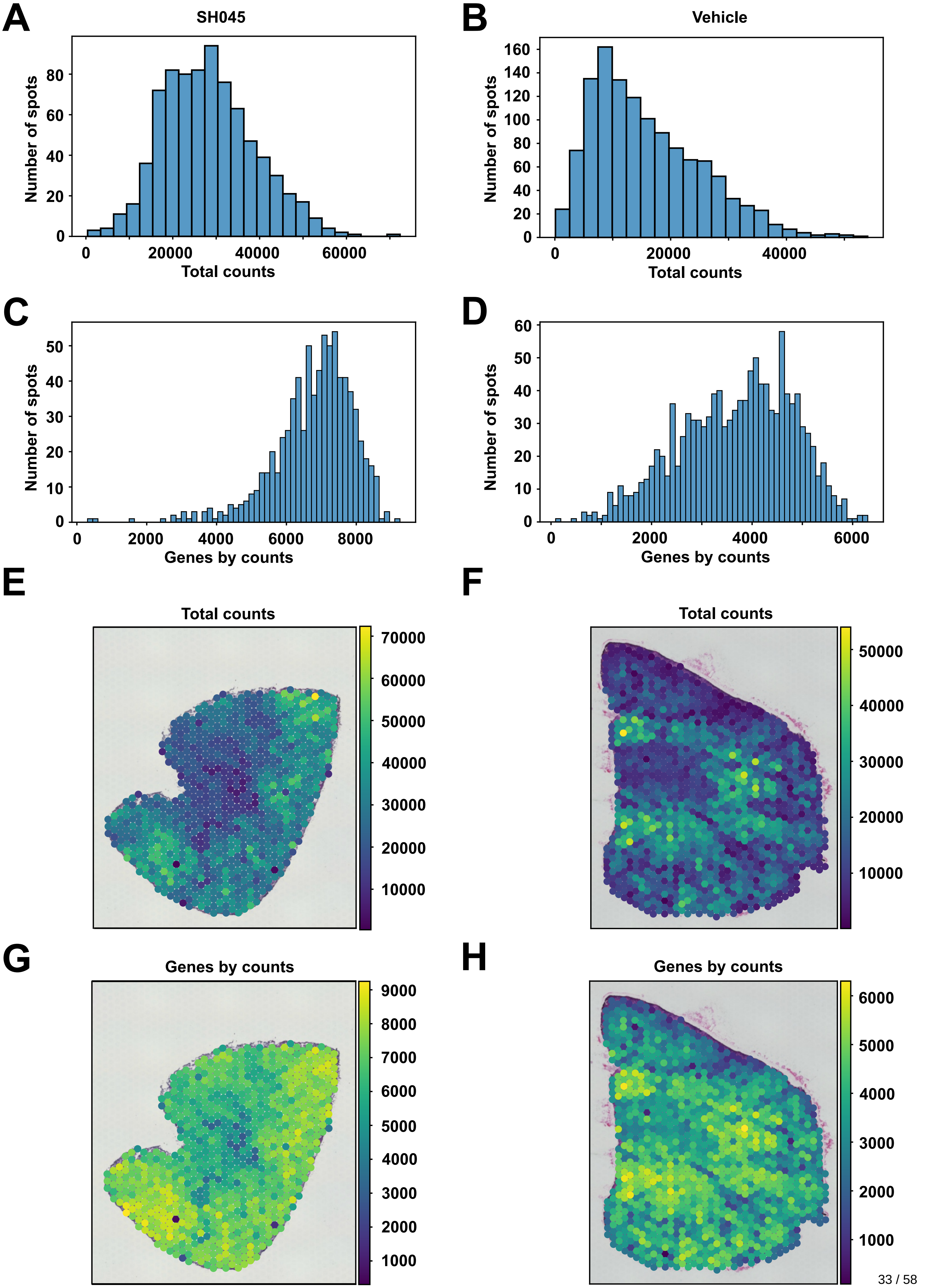

Supplementary Figure 17. Quality control summary of spatial transcriptomics data from mouse UUO kidney samples.

Histograms showing the distribution of total RNA molecules per spot in SH045 **(A)** and Vehicle **(B)** samples. Histograms showing the distribution of number of detected genes per spot in SH045 **(C)** and Vehicle **(D)** samples. Spatial plots visualize the spatial distribution of total RNA molecules per spot in SH045 **(E)** and Vehicle **(F)** samples. Spatial plots visualize the spatial distribution of number of detected genes per spot in SH045 **(G)** and Vehicle **(H)** samples.

Characterization of spatial transcriptomics data using cell-type deconvolution

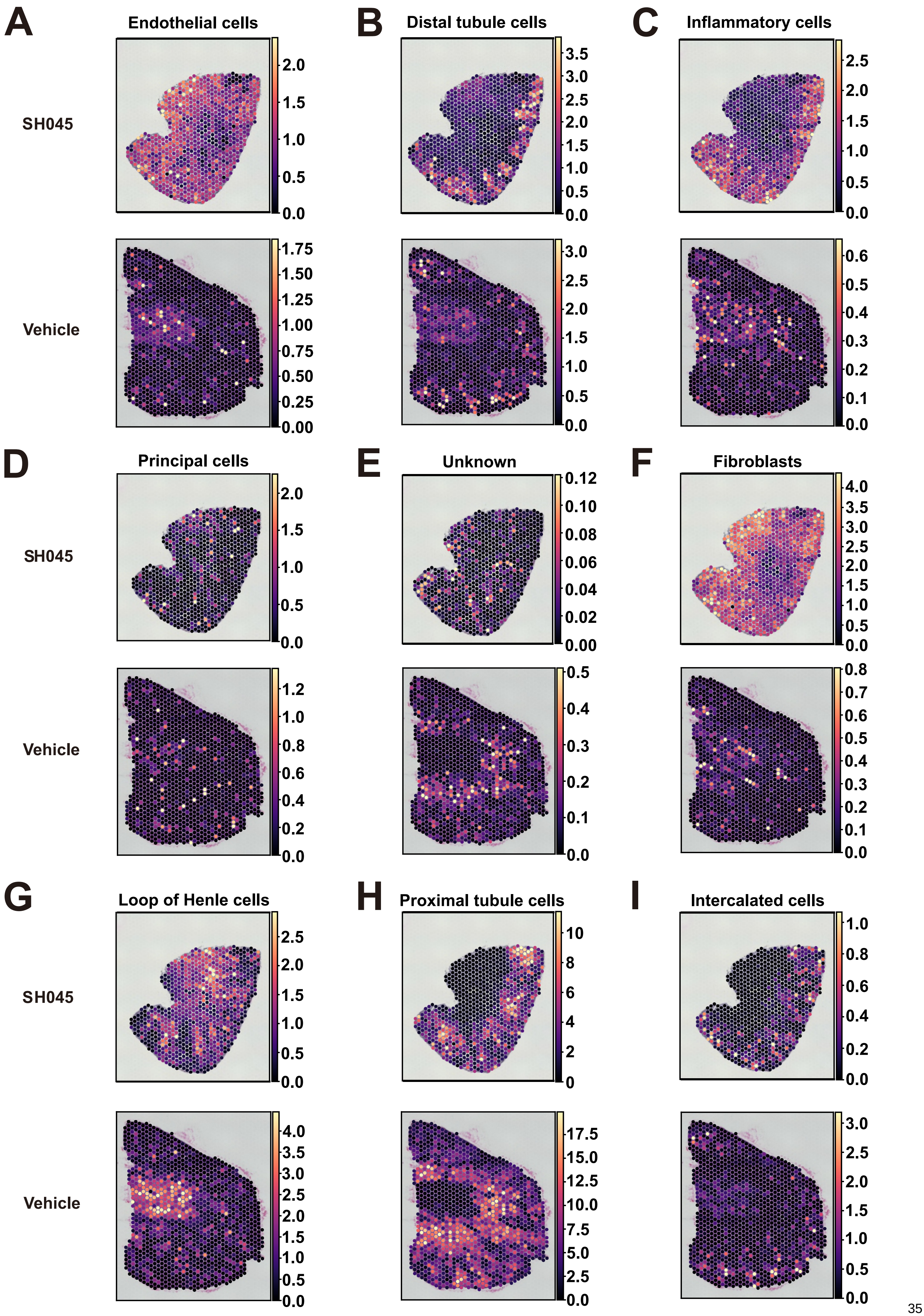

Supplementary Figure 18. Characterization of spatial transcriptomics data using cell-type deconvolution.

Spatial plots depicting estimated cell type proportions (color intensity) for all major cell types including endothelial cells **(A)**, distal tubule cells **(B)**, infammatory cells **(C)**, principal cells **(D)**, unknown **(E)**, fibroblasts **(F)**, loop of Henle cells **(G)**, proximal tubule cells **(H)** and intercalated cells **(I)**.

CD31 and Ki67 colocalization in 2m post I/R mouse kidneys

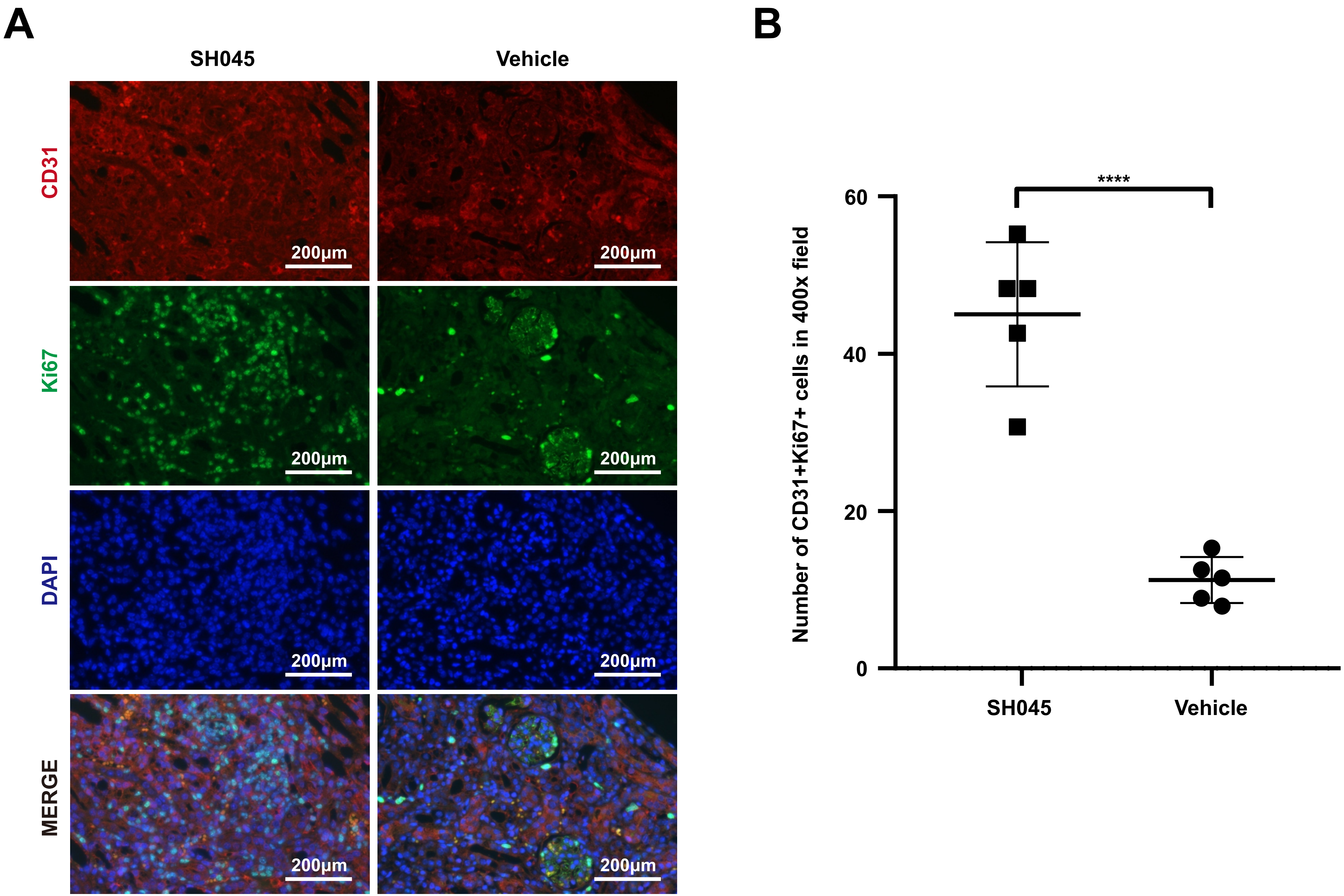

Supplementary Figure 19. CD31 and Ki67 colocalization in 2m post I/R mouse kidneys.
(A) Representative immunofluorescence staining images of CD31 and Ki67 colocalization in SH045 (left panel) and Vehicle (right panel) treated 2m post I/R mouse kidneys (magnification: 400x, scale bar: 200μm). **(B)** Quantification of CD31+Ki67+ cells in kidney sections from SH045 and Vehicle treated 2m post I/R mice. (SH045 n=5, Vehicle n=5). Data expressed as mean ± SEM. Mann-Whitney U-test was used. **** $p < 0.0001$.

Representative immunofluorescence staining images of CD31 and F4/80 colocalization in kidneys from UUO and 2m post I/R mice

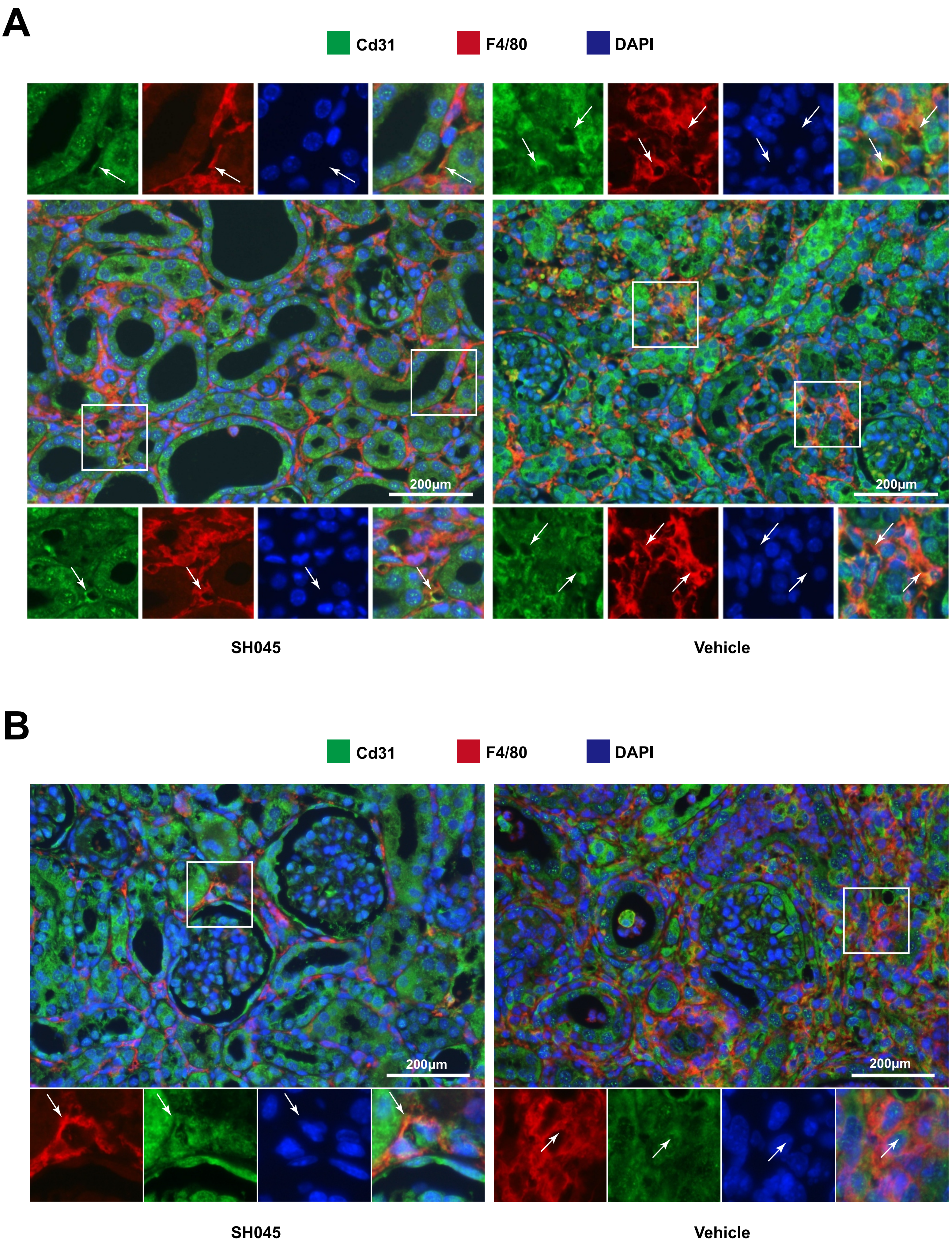

Supplementary Figure 20. Representative immunofluorescence staining images of CD31 and F4/80 colocalization in kidneys from UUO and 2m post I/R mice.

Representative immunofluorescence staining images of CD31 and F4/80 colocalization in kidneys from SH045 (left panel) and Vehicle (right panel) treated UUO **(A)** and 2m post I/R **(B)** mice. (magnification: 400x, scale bar: 200μm).

Resolving spatial distribution of novel endothelial cells and ECM activity in a mouse UUO model

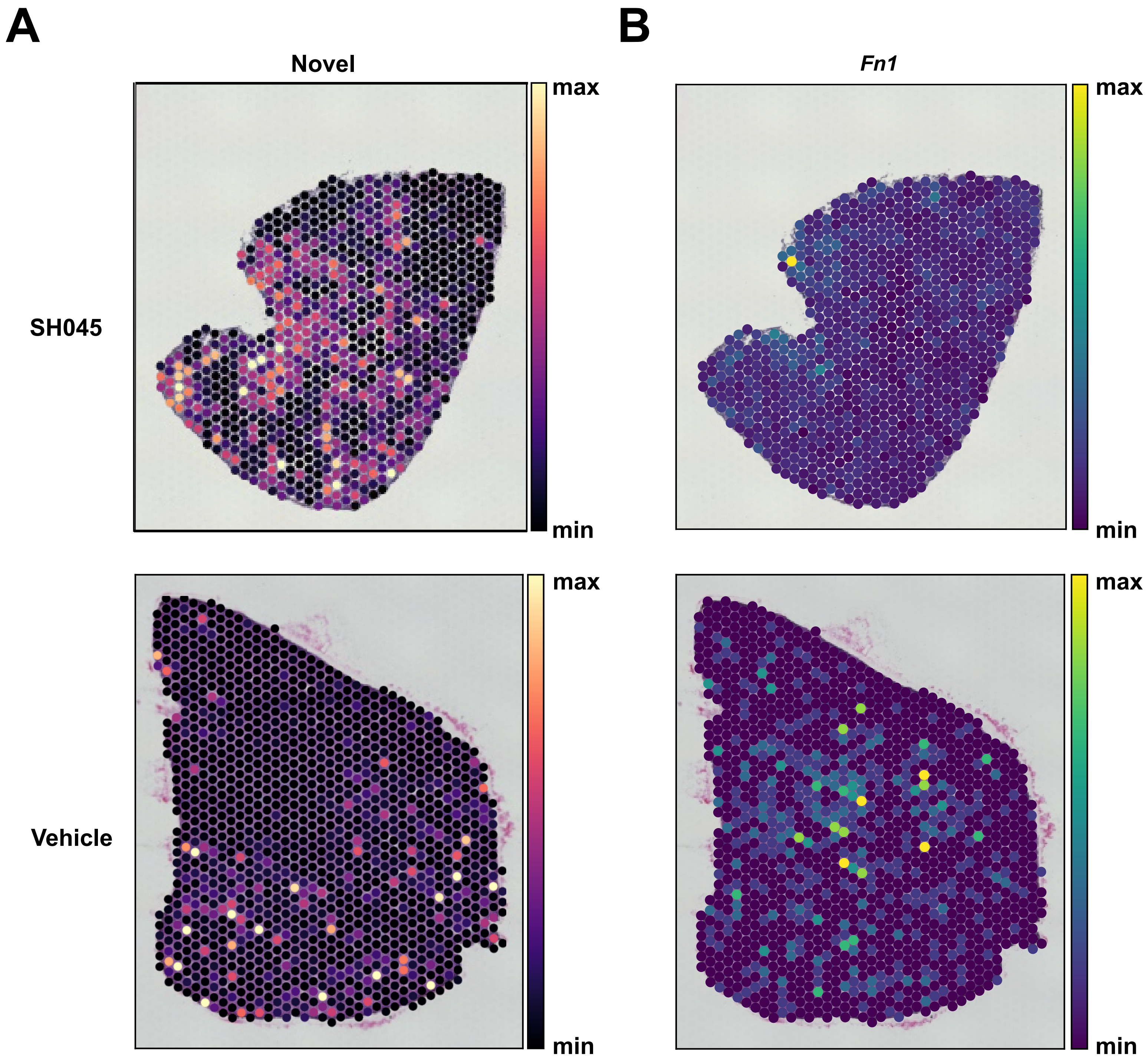

Supplementary Figure 21. Resolving spatial distribution of novel endothelial cells and ECM activity in a mouse UUO model.

(A) Distribution of novel endothelial cells in samples from SH045 (upper panel) and Vehicle (lower panel) group. **(B)** Activity of *Fn1* in SH045 (upper panel) and Vehicle (lower panel) group.

Expression of *Scara5* in all cell types

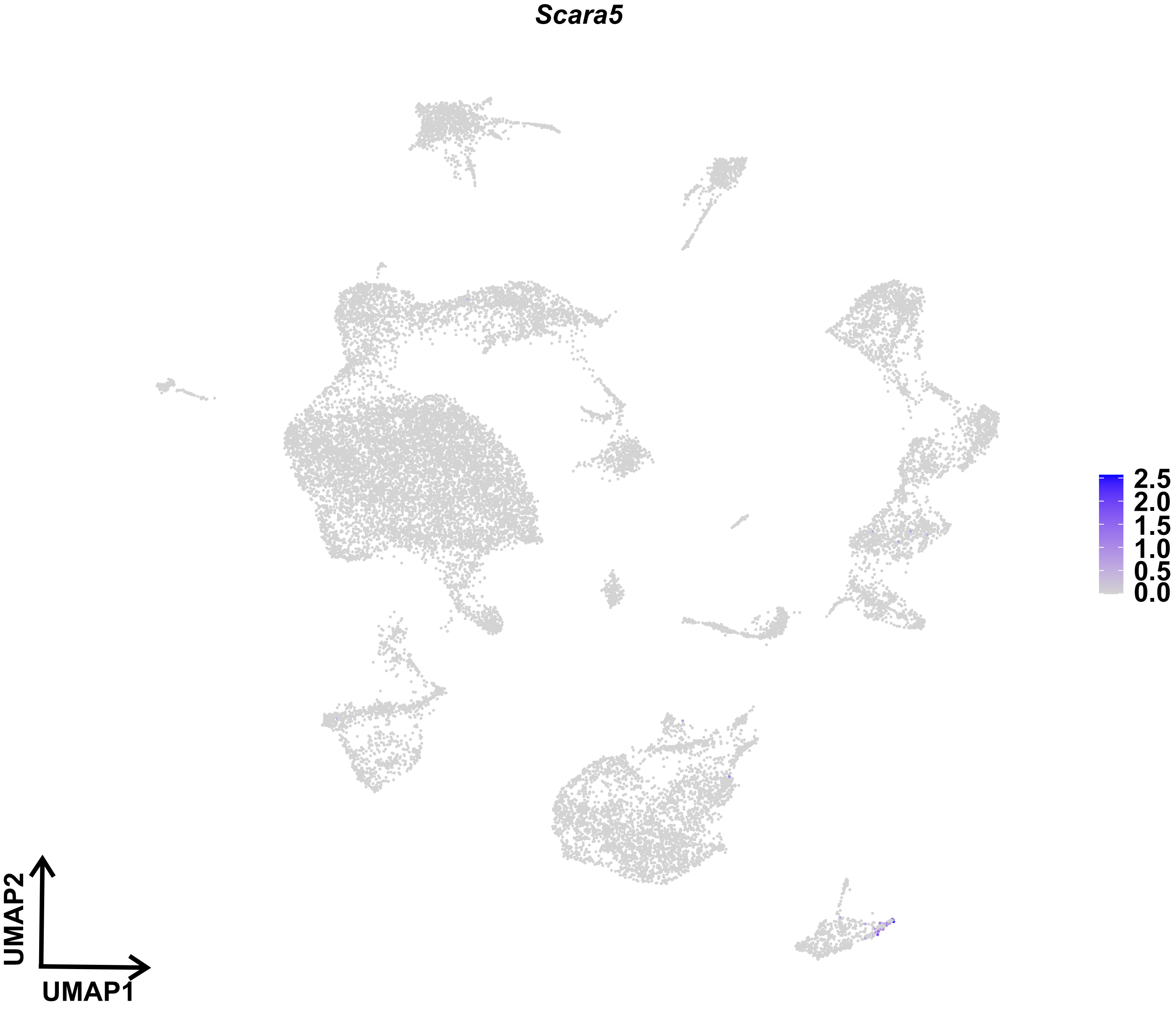

Supplementary Figure 22. UMAP plots showing expression of *Scara5* in all cell types.

Expression of α-SMA and SCARA5 in CKD-patients and 2m post I/R mice

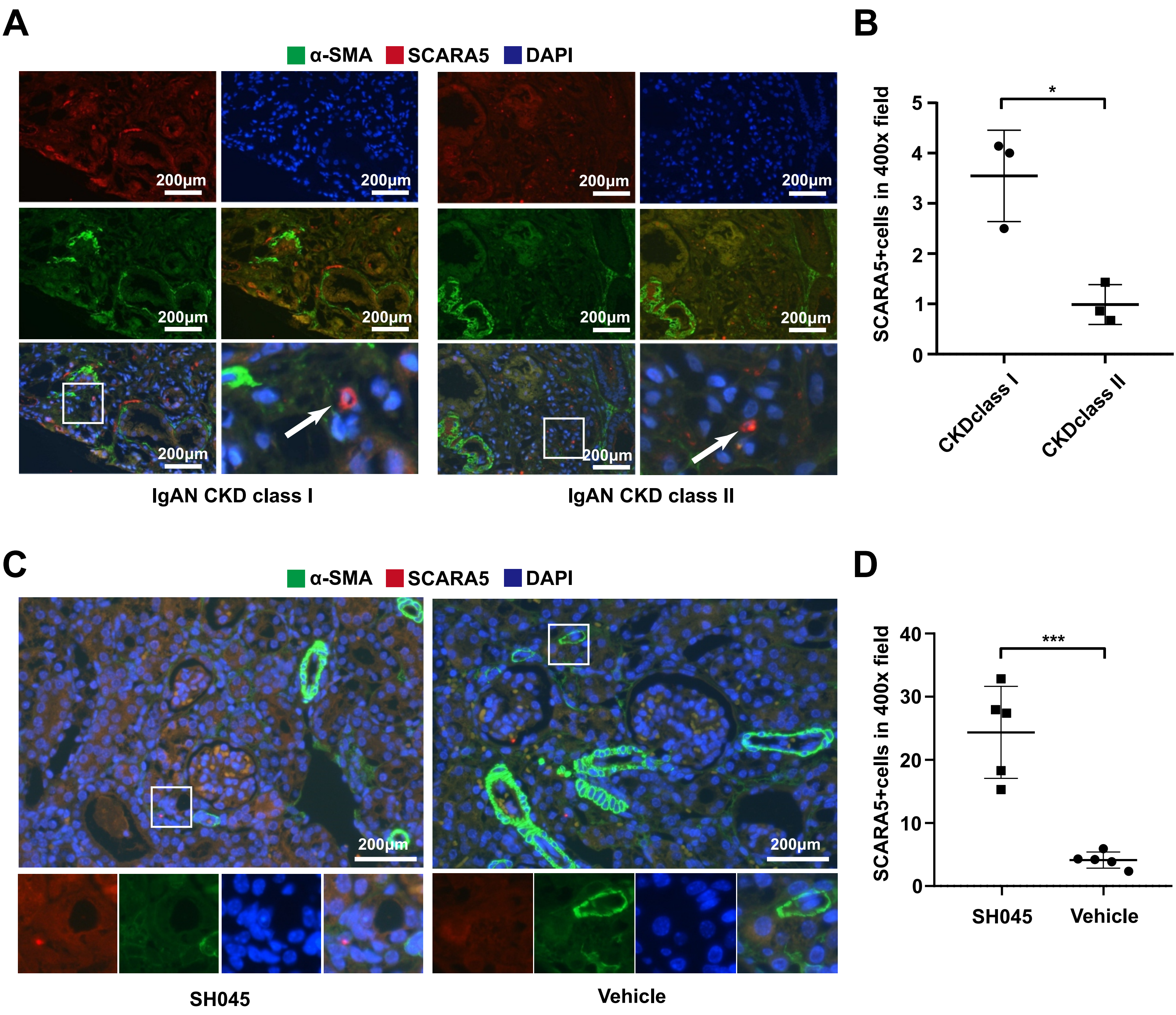

Supplementary Figure 23. Expression of α-SMA and SCARA5 in CKD-patients and 2m post I/R mice.

(A) Representative immunofluorescence staining images of α-SMA and SCARA5 colocalization of kidneys from IgAN CKD class I (left panel) and IgAN CKD class II (right panel) patients. (magnification: 400x, scale bar: 200μm). **(B)** Quantification of SCARA5+ cells in kidney sections from patients with IgAN. (IgAN CKD class I, n=3; IgAN CKD class II, n=3). **(C)** Representative immunofluorescence staining images of α-SMA and SCARA5 colocalization of kidneys from SH045 (left panel) and Vehicle (right panel) treated 2m post I/R mice (magnification: 400x, scale bar: 200μm). **(D)** Quantification of SCARA5+ cells in kidney sections from SH045 and Vehicle treated 2m post I/R mice. (SH045 n=5, Vehicle n=5). Data expressed as mean ± SEM. Mann-Whitney U-test was used. * $p < 0.05$, *** $p < 0.001$. IgAN: IgA nephropathy, CKD: chronic kidney diseases, CKD class I: GFR ≥ 90ml/min·1.73m^2, CKD class II: 60 ≤ GFR ≤ 89ml/min·1.73m^2.

PrP and Collagen III colocalization of kidneys from 2m post I/R mice

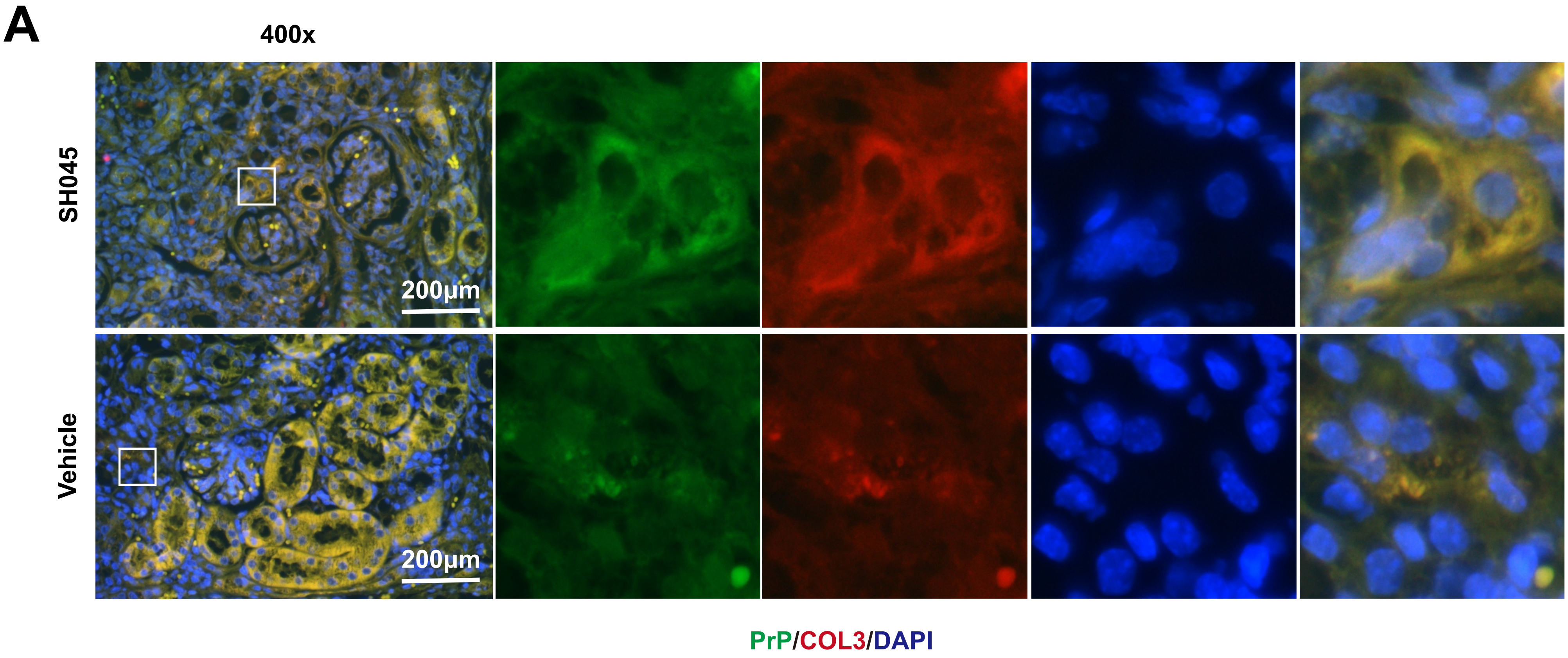

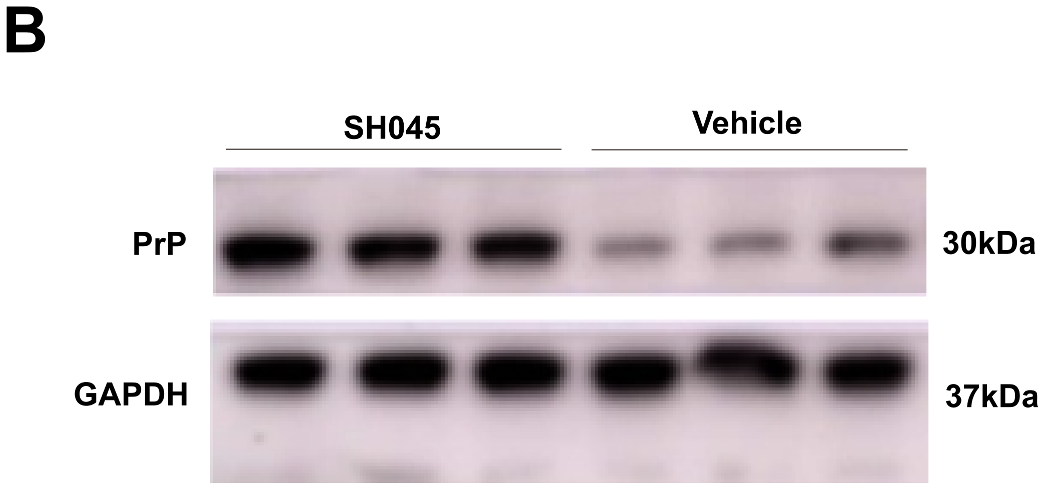

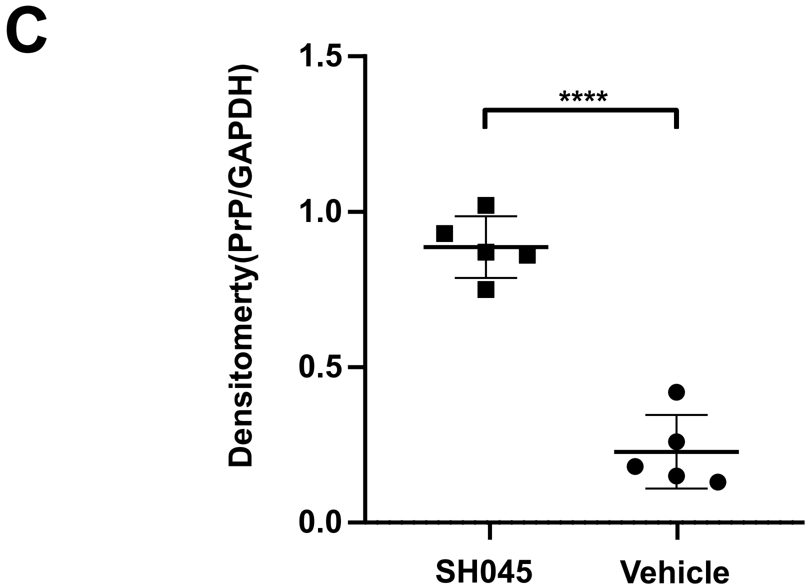

Supplementary Figure 24. PrP and Collagen III colocalization of kidneys from 2m post I/R mice.

(A) Representative immunofluorescence staining images of PrP and Collagen III colocalization of kidneys from SH045 (upper panel) and Vehicle (lower panel) treated 2m post I/R mice (magnification: 400x, scale bar: 200 μm). **(B)** Representative Western blot of and relative densitometric graphs of GAPDH, PrP in SH045 and Vehicle treated 2m post I/R mice. **(C)** Quantification of PrP expression normalized to GAPDH levels. (SH045 n=5, Vehicle n=5). Mann-Whitney U-test was used. **** $p < 0.0001$.

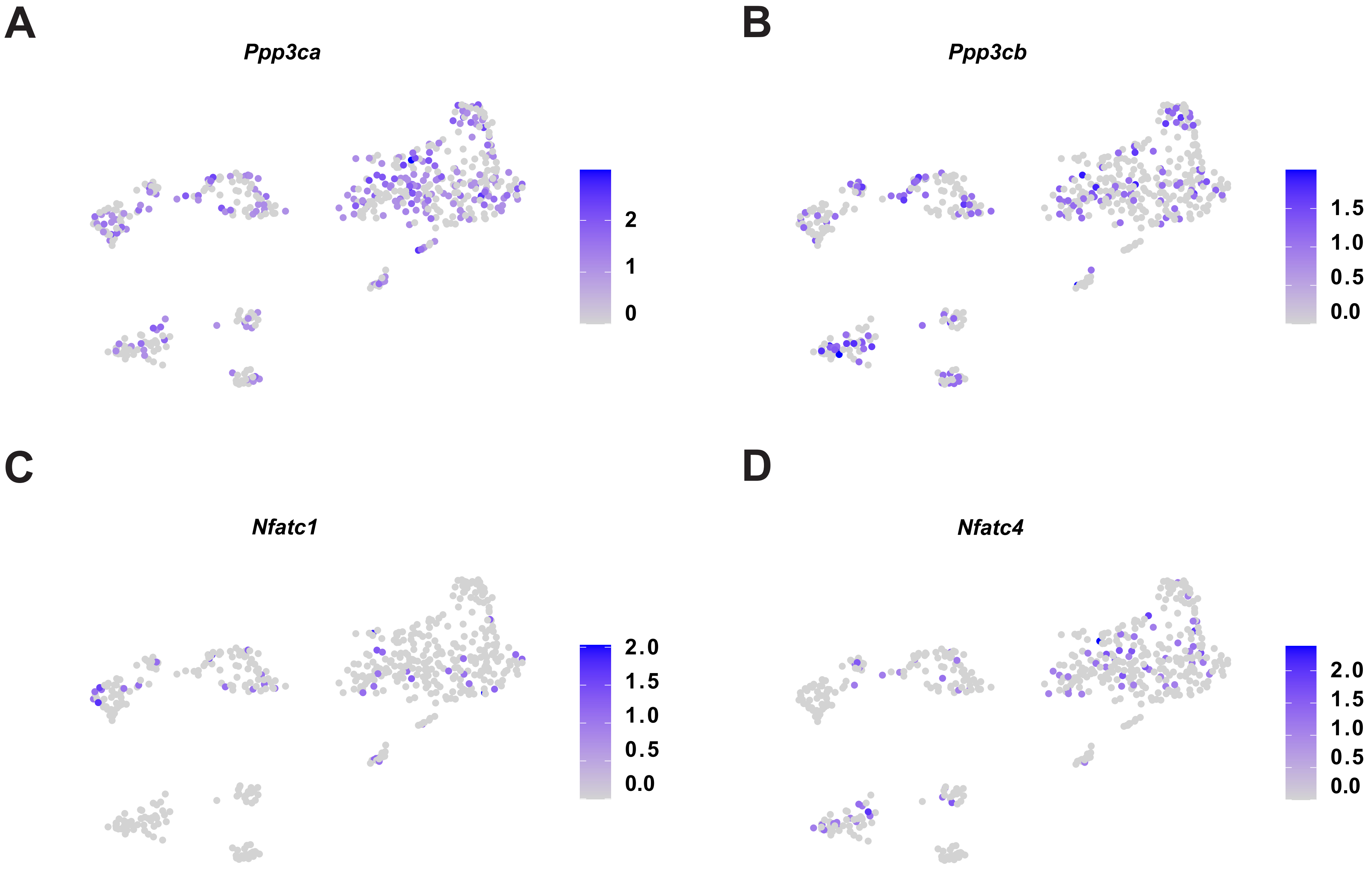

A
Ppp3ca
2
1
0
B
Ppp3cb
1.5
1.0
0.5
0.0
C
Nfatc1
2.0
1.5
1.0
0.5
0.0
D
Nfatc4
2.0
1.5
1.0
0.5
0.0

Supplementary Figure 25. UMAP plots showing expression of Calcineurin/ Nuclear factor of activated T cells (NFAT) related genes in fibroblasts.
Expression of *Ppp3a* **(A)**, *Ppp3b* **(B)**, *Nfatc1* **(C)**, *Nfatc4* **(D)**.

Cell-cell interactions between different cell types taking in into account spatial information

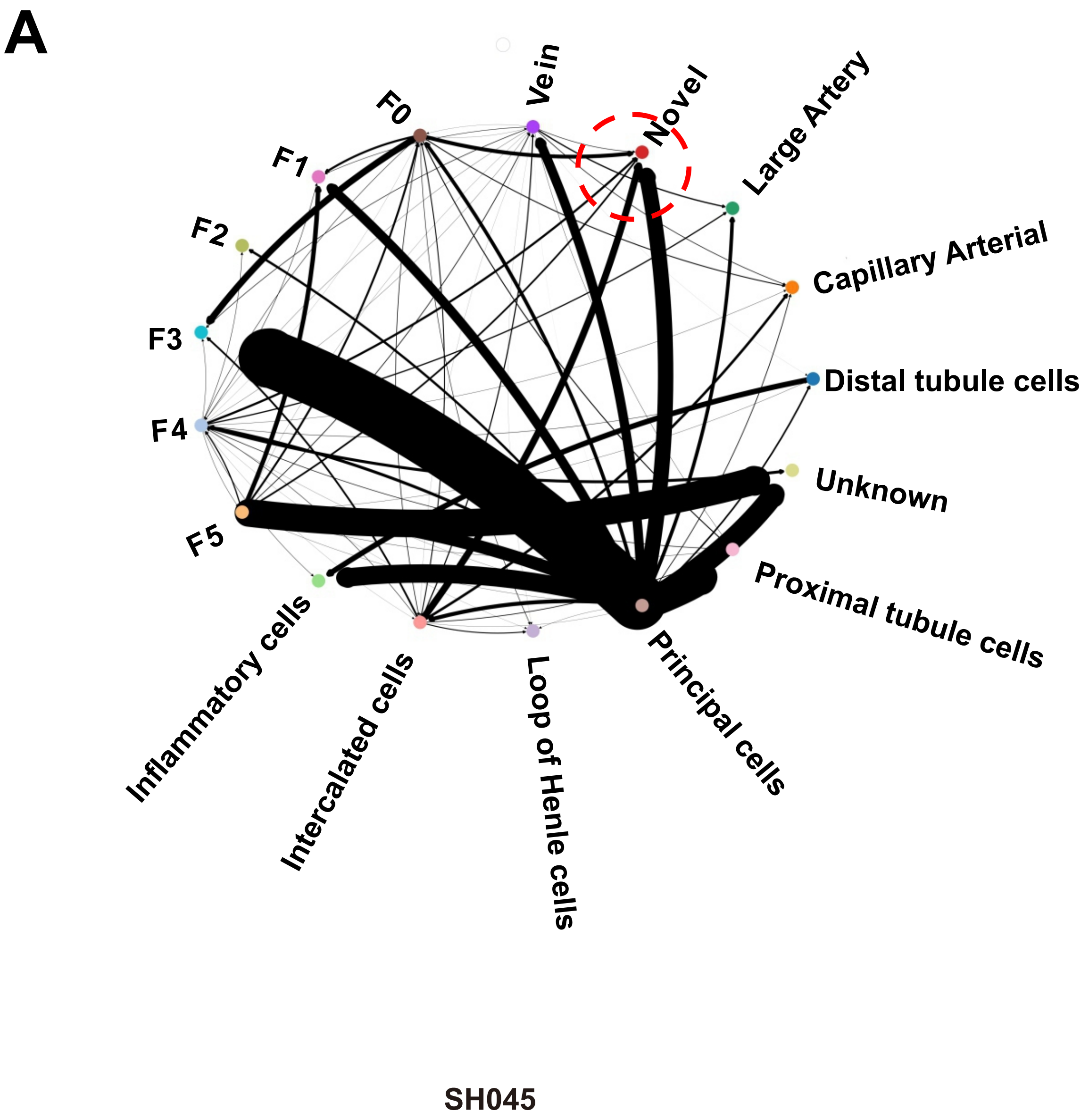

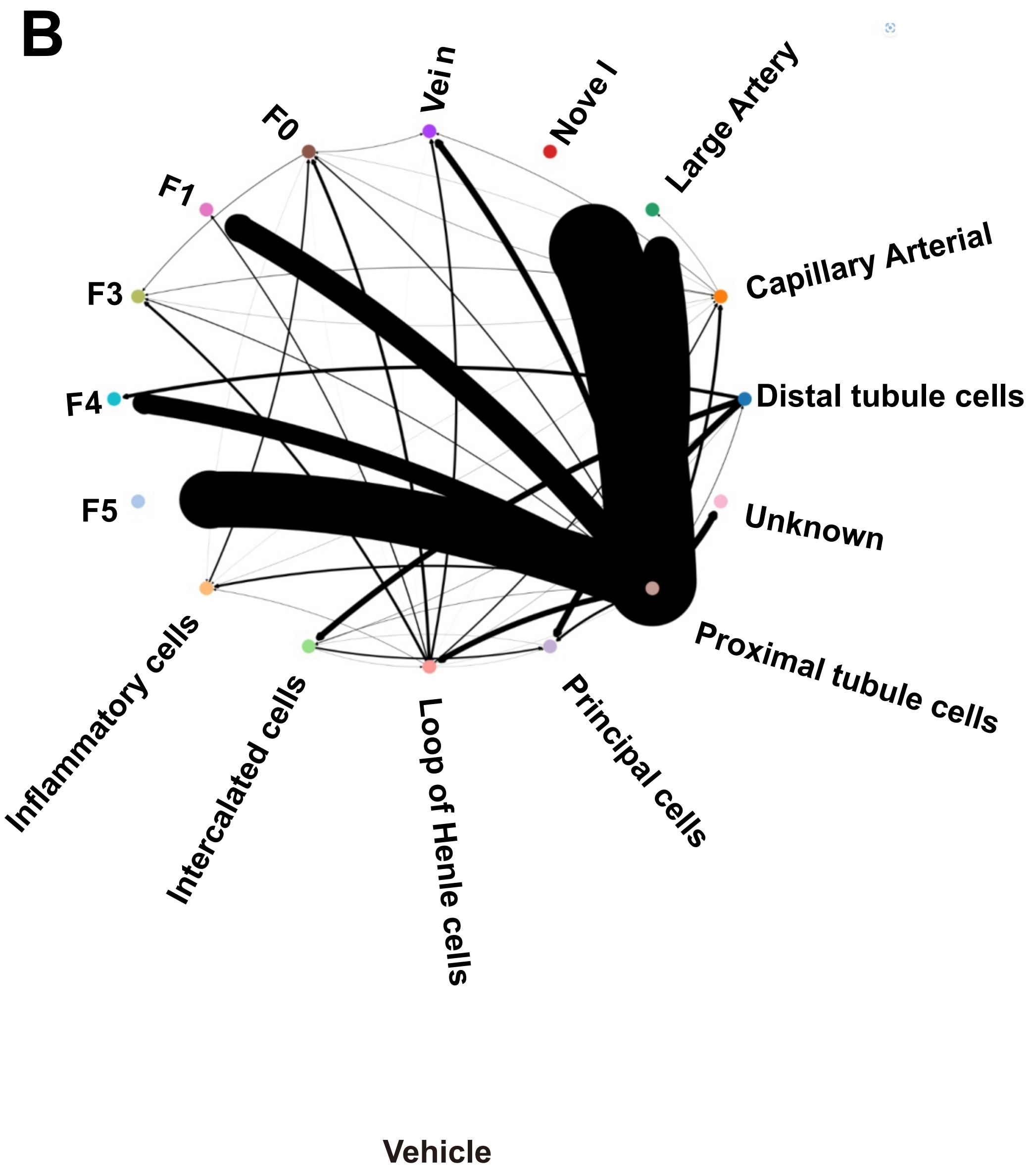

Supplementary Figure 26. Cell-cell interactions between different cell types taking in into account spatial information.

Type coupling heatmap of the SH045 **(A)** and Vehicle **(B)** group samples, with edge width proportional to the number of differentially expressed genes at a false-discovery-rate-corrected p-value threshold of 0.05 for each pair of sender and receiver cell types. Only edges with at least 200 genes are shown.

Alterations in network structure and signaling strength of putative cell-cell communications in all cell subpopulations

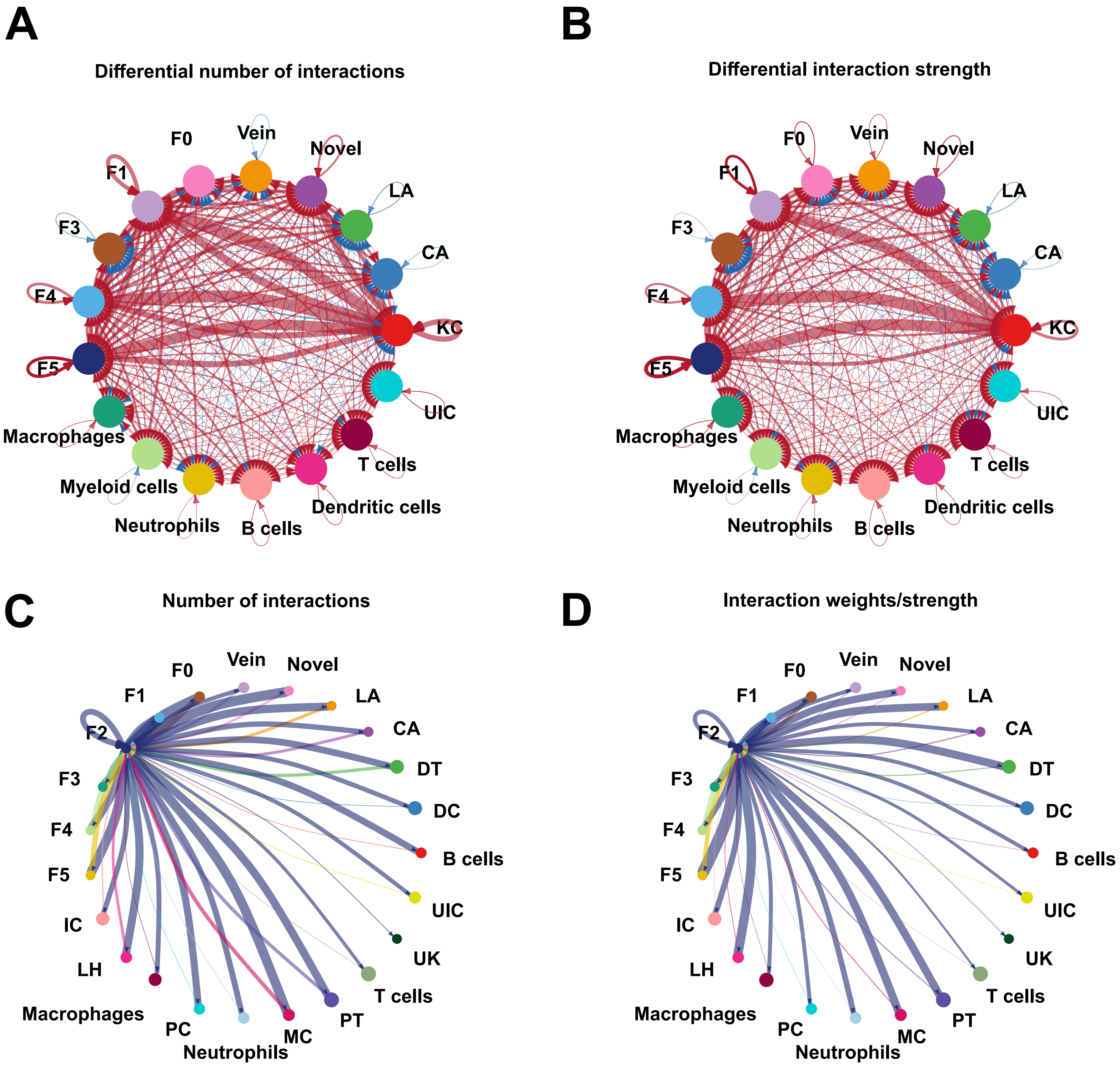

Supplementary Figure 27. Alterations in network structure and signaling strength of putative cell-cell communications in all cell subpopulations.

Differential number **(A)** and strength **(B)** of possible interactions among all cell subtypes. Red and blue lines indicate higher or lower number of predicted interactions in SH045 and Vehicle group, respectively. Circle diagrams showing all signals in F2 fibroblasts. The thickness of the lines indicates relative number **(C)** and strength **(D)** of cell-cell interactions.

Alterations in network structure and signaling strength of putative cell-cell communications in kidneys isolated from Ctrl and Vehicle group mice

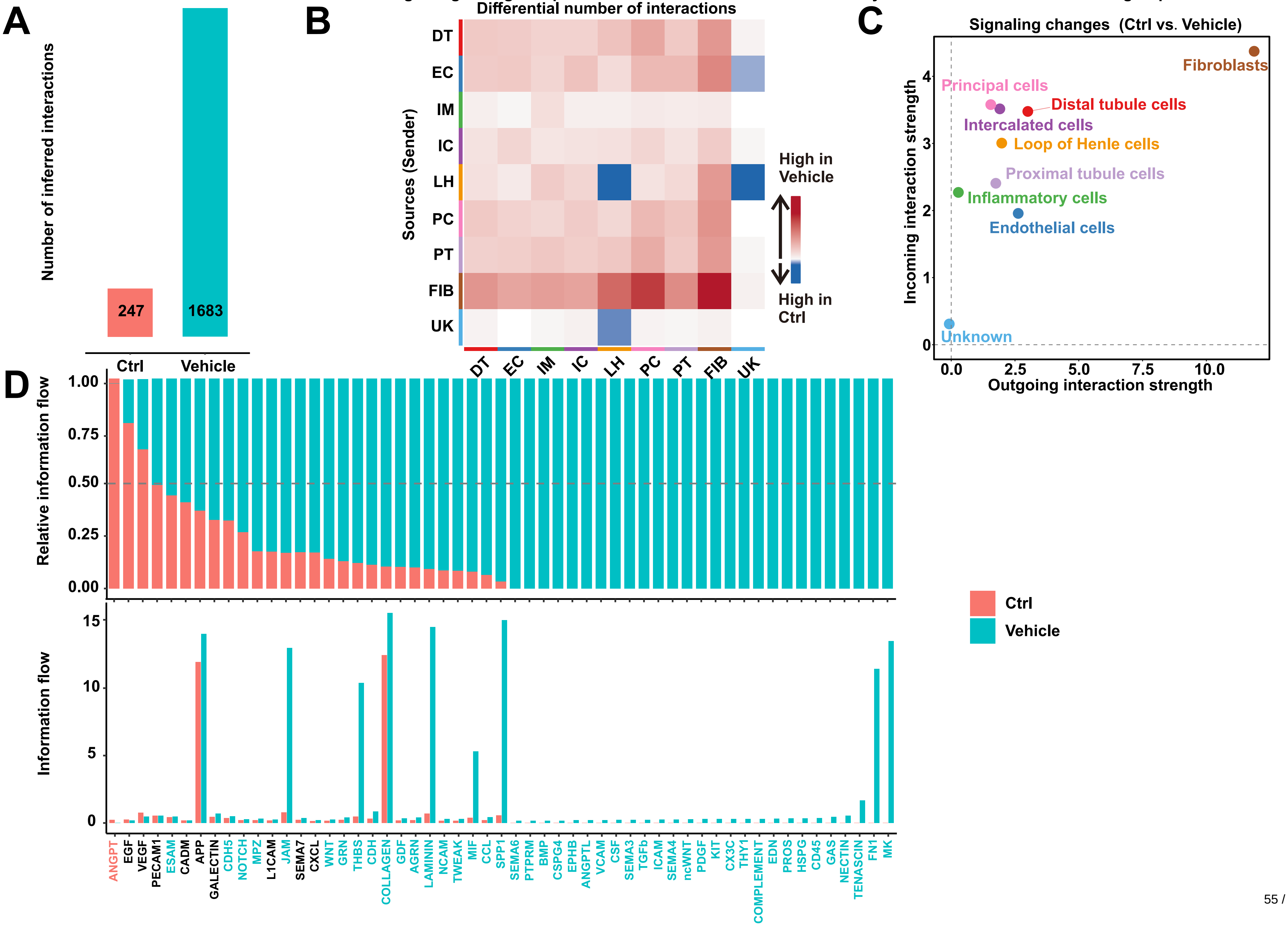

Supplementary Figure 28. Alterations in network structure and signaling strength of putative cell-cell communications in kidneys isolated from Ctrl and Vehicle group mice.

(A) Total number of possible interactions. **(B)** Differential number of possible interactions between cell populations. Red (positive values) and blue (negative values) in the color bar indicate higher number of predicted interactions in Vehicle versus Ctrl group, respectively. **(C)** Differential interaction analysis identifying prominently altered signaling sources and targets. **(D)** Significant signaling pathways were ranked based on their differences in relative information flow (upper panel) and absolute information flow (lower panel). Differences were calculated by summarizing all communication probabilities in each inferred network. DT, Distal tubule cells; IC, Intercalated cells; LH, Loop of Henle cells; PC, Principal cells; PT, Proximal tubule cells; UK, Unknown; IM, Inflammatory cells; FIB, Fibroblasts.

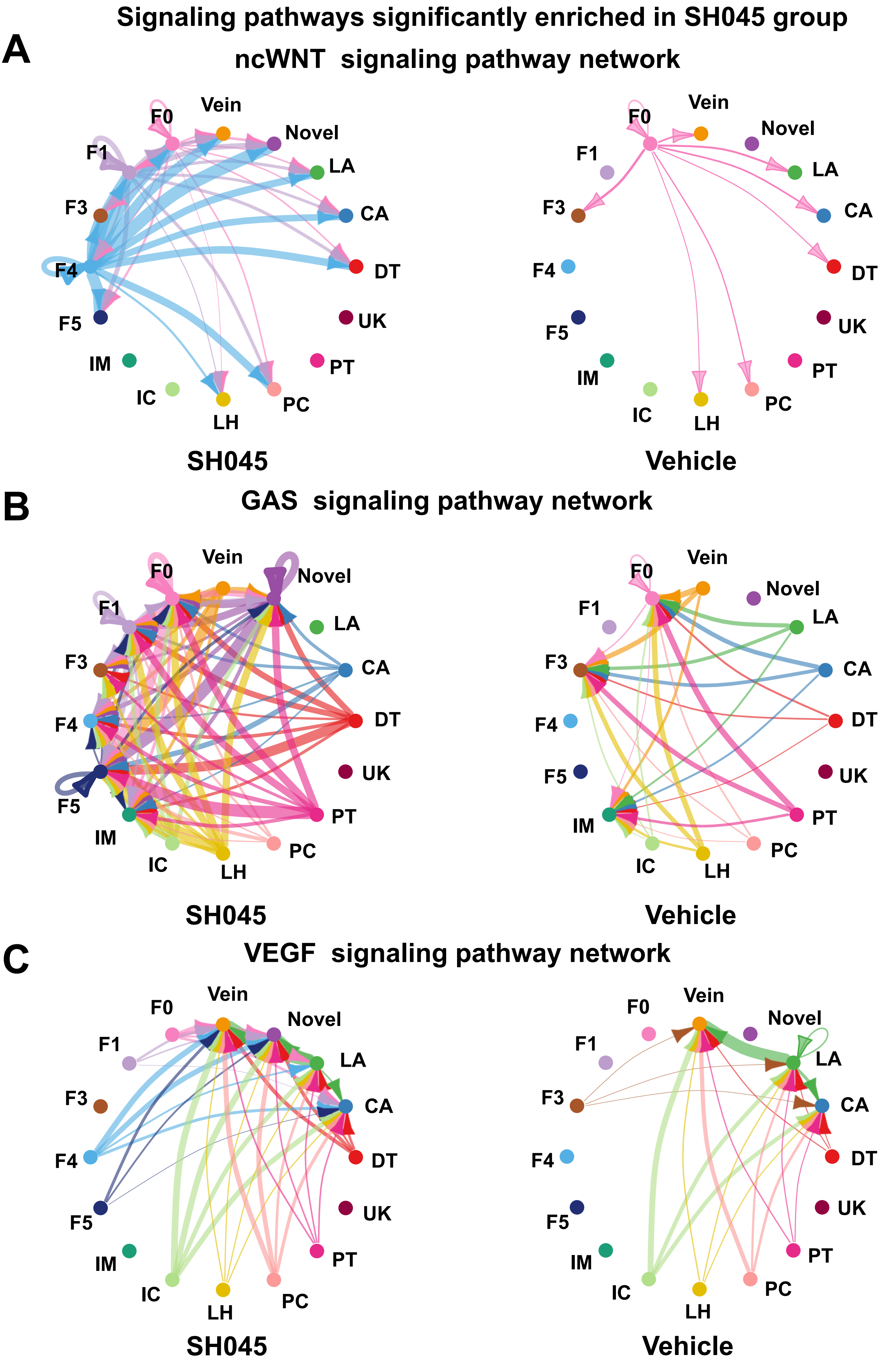
Signaling pathways significantly enriched in SH045 group
A
ncWNT signaling pathway network
F0
Vein
Novel
F1
LA
F3
CA
F4
DT
F5
UK
IM
PT
IC
PC
LH
SH045
Vehicle
B
GAS signaling pathway network
SH045
Vehicle
C
VEGF signaling pathway network
SH045
Vehicle

Supplementary Figure 29. Signaling pathways significantly enriched in SH045 group. Circle diagrams showing all signals in noncanonical (nc)WNT signaling pathway network **(A)**, GAS signaling pathway network **(B)**, and VEGF signaling pathway network **(C)**. The thickness of the lines indicates relative number of cell-cell interactions.
