# Supplementary material for: Single‐Cell RNA Sequencing Delineates Renal Anti‐Fibrotic Mechanisms Mediated by TRPC6 Inhibition
Source: Adv Sci (Weinh). 2025 Jun 17;12(33):e01175. doi: 10.1002/advs.202501175 (PMC12412467; doi:10.1002/advs.202501175)
Supplement: Supplementary file 2 — Supporting Information [file ADVS-12-e01175-s003.docx]

**Supplementary Table 1.** Differentially expressed genes (DEGs) in all major cell types and inflammatory cell subsets.

Significantly upregulated **(Sheet 1)** and downregulated **(Sheet 2)** genes in endothelial cells in the SH045 group. Significantly upregulated **(Sheet 3)** and downregulated **(Sheet 4)** genes in fibroblasts in the SH045 group. Significantly upregulated **(Sheet 3)** and downregulated **(Sheet 4)** genes in distal tubule cells in the SH045 group. Significantly upregulated **(Sheet 5)** and downregulated **(Sheet 6)** genes in inflammatory cells in the SH045 group. Significantly upregulated **(Sheet 7)** and downregulated **(Sheet 8)** genes in intercalated cells in the SH045 group. Significantly upregulated **(Sheet 9)** and downregulated **(Sheet 10)** genes in loop of Henle cells in the SH045 group. Significantly upregulated **(Sheet 11)** and downregulated **(Sheet 12)** genes in principal cells in the SH045 group. Significantly upregulated **(Sheet 13)** and downregulated **(Sheet 14)** genes in proximal tubule cells in the SH045 group. Significantly upregulated **(Sheet 15)** and downregulated **(Sheet 16)** genes in unknown in the SH045 group. Significantly upregulated **(Sheet 17)** and downregulated **(Sheet 18)** genes in B cells in the SH045 group. Significantly upregulated **(Sheet 19)** and downregulated **(Sheet 20)** genes in dendritic cells in the SH045 group. Significantly upregulated **(Sheet 21)** and downregulated **(Sheet 22)** genes in macrophages in the SH045 group. Significantly upregulated **(Sheet 23)** and downregulated **(Sheet 24)** genes in myeloid cells in the SH045 group. Significantly upregulated **(Sheet 25)** and downregulated **(Sheet 26)** genes in neutrophils in the SH045 group. Significantly upregulated **(Sheet 27)** and downregulated **(Sheet 28)** genes in unknown inflammatory cells in the SH045 group. Significantly upregulated **(Sheet 29)** and downregulated **(Sheet 30)** genes in T cells in the SH045 group.

**Supplementary Table 2.** Gene set overrepresentation analysis results for endothelial cells and fibroblasts.

Upregulated **(Sheet 1)**, the functional annotation clustering **(Sheet** **2)** and unclustered **(Sheet** **3)** downregulated GO terms as determined by DAVID in endothelial cells in the SH045 group. Upregulated **(Sheet 4)**, the functional annotation clustering **(Sheet** **5)** and unclustered **(Sheet** **6)** downregulated GO terms as determined by DAVID in fibroblasts in the SH045 group.

**Supplementary Table 3.** Similarity between endothelial cell subpopulations detected in UUO mouse model and in the endothelial cell atlas.

**Supplementary Table 4.** Marker genes and gene set overrepresentation analysis results for novel endothelial cells and F2 fibroblasts.

Marker genes in novel endothelial cells **(Sheet 1)**. The functional annotation clustering **(Sheet** **2)** and unclustered **(Sheet** **3)** GO terms as determined by DAVID in novel endothelial cells. Marker genes in F2 fibroblasts **(Sheet 4)**. The functional annotation clustering **(Sheet** **5)** and unclustered **(Sheet** **6)** GO terms as determined by DAVID in F2 fibroblasts.
